# Supplementary material for: The relationship between patient experience and real-world digital health access in primary care: A population-based cross-sectional study
Source: PLoS One. 2024 May 7;19(5):e0299005. doi: 10.1371/journal.pone.0299005 (PMC11075820; doi:10.1371/journal.pone.0299005)
Supplement: S1 Appendix — (DOCX) [file pone.0299005.s001.docx]

### S1 Appendix: Summary of coding done on dependent and independent variables.

| **Database** | **Variable** | **Additional coding** | **Responses** |
| --- | --- | --- | --- |
| **Dependant Variables** | | | |
| HCES | Summed patient experience score | Coded ordinal to interval scale (“Always” = 5, “”Often” = 4, “Sometimes” = 3, “Rarely” = 2, “Never” = 1). Coded responses of “Volunteers it depends who they see/what they are there for”, “Not Applicable”, “Don’t know”, and “Refused” as NA:   - When you see your provider or someone else in their office, how often do they know important information about your medical history? - When you see your provider or someone else in their office, how often do they give you an opportunity to ask questions about recommended treatment? - When you see your provider or someone else in their office, how often do they spend enough time with you? - When you see your provider or someone else in their office, how often do they involve you as much as you want to be in decisions about your care and treatment? - When you see your provider or someone else in their office, how often do they explain things in a way that is easy to understand?   Summed interval values across all items to provide summed score. Categorized “poor experience” as 1^st^ quintile (score ≤ 21) and “positive experience” as remaining quintiles. | Poor experience, Positive experience |
| HCES | Doctor/office: Knows important information about medical history | Combined “Sometimes”, “Rarely”, and “Never” into category: “Poor experience”  Combined “Always”, and “Often”, into category: “Positive experience” | Poor experience, Positive experience |
| HCES | Doctor/office: Opportunity to ask questions | Combined “Sometimes”, “Rarely”, and “Never” into category: “Poor experience”  Combined “Always”, and “Often”, into category: “Positive experience” | Poor experience, Positive experience |
| HCES | Doctor/office: Spend enough time with you | Combined “Sometimes”, “Rarely”, and “Never” into category: “Poor experience”  Combined “Always”, and “Often”, into category: “Positive experience” | Poor experience, Positive experience |
| HCES | Doctor/office: Involve you in decisions about your care and treatment | Combined “Sometimes”, “Rarely”, and “Never” into category: “Poor experience”  Combined “Always”, and “Often”, into category: “Positive experience” | Poor experience, Positive experience |
| HCES | Doctor/office: Explain things in easy to understand terms | Combined “Sometimes”, “Rarely”, and “Never” into category: “Poor experience”  Combined “Always”, and “Often”, into category: “Positive experience” | Poor experience, Positive experience |
| HCES | Same day/next day | Combined values ≤ 1: “Same-/Next-day visit”  Combined values > 1: “Later visit” | Same/next-day, Later |
| **Independent Variables** | | | |
| HCES | Telehealth access | If *yes* to any of the following questions:   - In the last 12 months have you emailed your provider with a medical question? - In the last 12 months have you asked your provider a medical question online, such as through a website or portal? - In the last 12 months have you asked your provider a medical using video, for example a telemedicine appointment through OTN? - In the last 12 months have you communicated with your provider about your medical care using electronic messaging, such as text or instant messaging? - Other than the methods we have already asked you about, in the last 12 months, have you communicated with your provider using any other online or digital tools? - Not including visits that we have already asked about, in the last 12 months have you received medical care online or through a digital tool from a physician other than your own without having to make an in-person visit? Interviewer if required: Examples of this type of care include Appletree, Akira, DermaGo, GOeVisit and Maple. | Yes, No |
| HCES | Digital health record access | If *yes* to any of the following questions:   - In the last 12 months, have you looked at your medical records using an online system or digital tool? - In the last 12 months, have you looked at your medical records using online systems or digital tools that are designed for people with specific health conditions? An example of this type of tool is NED or Medly. - In the last 12 months, have you used any online system or digital tool that keeps track of ALL your health records in one place? This would include records from your fd_type, any specialists you have seen, lab results, immunizations, etc.? Examples of this type of tool are Dot Health or MedChart. | Yes, No |
| HCES | Online booking access | In the last 12 months have you emailed or visited a website to set up an appointment with your provider? | Yes, No |
| RPDB | Age | Recoded continuous variable into 3 categories | 16-44, 45-64, 65+ |
| RPDB | Sex | No additional coding | Female, Male |
| HCES | Financial situation | Combined “Tight”, “Poor”, and “Very Poor” into a single category: “Tight/Poor/Very Poor” | Very Comfortable, Comfortable, Tight/Poor/Very Poor |
| HCES | Primary language spoken | Combined all languages other than English into a single category: “Other” | English, Other |
| HCES | Educational attainment | Combined “Less than high school”, “Some high school”, and “High school graduate or equivalent” into category: “High school or less”.  Combined “Some community college, technical, trade, or vocational college” and “Some university but no degree” into category: “Some college/university”.  Combined “Completed community college, technical, trade, or vocational college” and “Completed bachelor’s degree (Arts, Science, Eng, etc.)” into category: “Completed college/university”.  Combined “Post graduate training: MA, MSc, MLS, MSW, MBA, etc.”, “Post graduate training: PhD, ‘Doctorate’”, and Professional degree (Law, Medicine, Dentistry)” into category: “Post-graduate/professional degree”. | High school or less, Some college/university, completed college/university, Post-graduate/professional degree |
| RPDB | Rurality categories | Categorized Rurality Index of Ontario (RIO) scores into ordinal categories.   - RIO scores equal to 0: “Large urban” - RIO scores ≥ 1 and less than 10: “Urban” - RIO scores ≥ 10 and less than 40: “Small urban” - RIO scores ≥ 40: “Rural”   Combined | Large urban, Urban, Small urban, Rural |
| HCES | Self-reported health | No additional coding | Poor, Fair, Good, Very good, Excellent |
| Johns Hopkins ACG® Version 10 | ADG Scores | Combined into categories: < 3, 3 – 4, 5 – 6, 7 – 8, ≥ 9 | < 3, 3 – 4, 5 – 6, 7 – 8, ≥ 9 |
| OHIP | Primary care encounters over past 12 months | Divided numeric variable into two categorized by median (3 encounters). | ≤ 3 encounters, > 3 encounters |
| ONMARG | Dependency | Used quintiles of the DA score. | 1^st^ quintile (least marginalized), 2^nd^ quintile, 3^rd^ quintile, 4^th^ quintile, 5^th^ quintile (most marginilized) |
| ONMARG | Material deprivation | Used quintiles of the DA score. | 1^st^ quintile (least marginalized), 2^nd^ quintile, 3^rd^ quintile, 4^th^ quintile, 5^th^ quintile (most marginilized) |
| ONMARG | Ethnic concentration | Used quintiles of the DA score. | 1^st^ quintile (least marginalized), 2^nd^ quintile, 3^rd^ quintile, 4^th^ quintile, 5^th^ quintile (most marginilized) |
| ONMARG | Residential instability | Used quintiles of the DA score. | 1^st^ quintile (least marginalized), 2^nd^ quintile, 3^rd^ quintile, 4^th^ quintile, 5^th^ quintile (most marginilized) |
| CAPE, CPDB, IPDB | Program type | Combined primary care payment models provided by the Ontario Ministry of Health  Combined “Comprehensive Care Model (CCM)” and Family Health Group (FHG) responses into category: “Enhanced Fee-for-Service”  Combined “Family Health Network (FHN)” and “Family Health Organization (FHO)” into category: “Capitation”  Combined all other responses into category: “Other” | Enhanced FFS, Capitation, Other |
| HCES | Number of years with provider | Combined into 4 categories: < 3, 4 – 9, 10 – 19, ≥ 20 | Less than 3, 4-9, 10-19, 20 or more |
| OHIP | Number of encounters/past 12 months | Combined into 2 categories: ≤ 3 encounters, > 3 encounters | ≤ 3 encounters, > 3 encounters |
