# Supplementary figures and images for: The relationship between patient experience and real-world digital health access in primary care: A population-based cross-sectional study
Source: PLoS One. 2024 May 7;19(5):e0299005. doi: 10.1371/journal.pone.0299005 (PMC11075820; doi:10.1371/journal.pone.0299005)

### S2 Appendix: Histogram of patient experience scores with binary groupings.

**
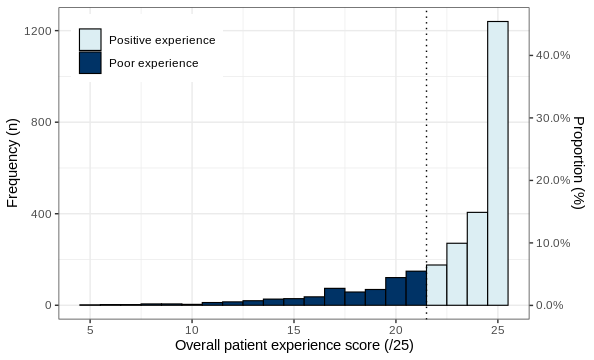
**

Supplement: S2 Appendix — (DOCX) [file pone.0299005.s002.docx]
