# Supplementary material for: The relationship between patient experience and real-world digital health access in primary care: A population-based cross-sectional study
Source: PLoS One. 2024 May 7;19(5):e0299005. doi: 10.1371/journal.pone.0299005 (PMC11075820; doi:10.1371/journal.pone.0299005)
Supplement: S3 Appendix — (DOCX) [file pone.0299005.s003.docx]

### S3 Appendix: Odds ratios and confidence intervals for single-item experience models

Associations between factors and primary care provider sometimes, rarely, or never knowing medical history, stratified by patient age.

| **Predictors** | **Full Model, N = 2,677** | |  | **Age ≤ 60 years, N = 1,378** | |  | **Age > 60 years, N = 1,308** | |  |
| --- | --- | --- | --- | --- | --- | --- | --- | --- | --- |
|  | **Odds Ratios** | **CI (95%)** | **p** | **Odds Ratios** | **CI (95%)** | **p** | **Odds Ratios** | **CI (95%)** | **p** |
| **Telehealth access** | 0.91 | 0.71 – 1.17 | 0.469 | 0.98 | 0.71 – 1.36 | 0.920 | 0.73 | 0.48 – 1.13 | 0.159 |
| **Health record access** | 0.35 | 0.11 – 0.88 | **0.048** | 0.31 | 0.07 – 0.91 | 0.060 | 0.31 | 0.02 – 1.66 | 0.271 |
| **Online booking access** | 1.33 | 0.69 – 2.38 | 0.363 | 1.13 | 0.55 – 2.19 | 0.720 | 1.84 | 0.40 – 6.05 | 0.363 |
| **Sex** |  |  |  |  |  |  |  |  |  |
| Female | Reference | |  |  |  |  |  |  |  |
| Male | 0.85 | 0.66 – 1.09 | 0.194 | 0.98 | 0.71 – 1.36 | 0.907 | 0.76 | 0.49 – 1.18 | 0.227 |
| **Financial Situation** |  |  |  |  |  |  |  |  |  |
| Very comfortable | Reference | |  |  |  |  |  |  |  |
| Comfortable | 1.32 | 0.93 – 1.90 | 0.128 | 1.17 | 0.74 – 1.89 | 0.513 | 1.32 | 0.75 – 2.43 | 0.356 |
| Tight/Very tight/Poor | 1.50 | 0.99 – 2.29 | 0.059 | 1.35 | 0.80 – 2.34 | 0.271 | 1.45 | 0.70 – 3.03 | 0.315 |
| **Educational Attainment** |  |  |  |  |  |  |  |  |  |
| High school or less | Reference | |  |  |  |  |  |  |  |
| Some college/university | 0.88 | 0.51 – 1.46 | 0.622 | 0.86 | 0.40 – 1.77 | 0.689 | 0.95 | 0.41 – 2.01 | 0.895 |
| Completed college/university | 1.41 | 1.04 – 1.93 | **0.028** | 1.39 | 0.90 – 2.22 | 0.151 | 1.25 | 0.78 – 2.02 | 0.353 |
| Post-graduate/professional degree | 1.30 | 0.85 – 2.00 | 0.225 | 1.29 | 0.73 – 2.29 | 0.387 | 1.13 | 0.50 – 2.37 | 0.755 |
| **Primary language spoken** |  |  |  |  |  |  |  |  |  |
| English | Reference | |  |  |  |  |  |  |  |
| Other | 1.55 | 1.10 – 2.16 | **0.011** | 1.08 | 0.71 – 1.61 | 0.724 | 2.99 | 1.57 – 5.52 | **0.001** |
| **Self-perceived health** |  |  |  |  |  |  |  |  |  |
| Poor | Reference | |  |  |  |  |  |  |  |
| Fair | 0.77 | 0.43 – 1.43 | 0.392 | 0.62 | 0.26 – 1.58 | 0.296 | 0.89 | 0.39 – 2.19 | 0.796 |
| Good | 0.82 | 0.47 – 1.48 | 0.485 | 0.70 | 0.32 – 1.66 | 0.390 | 0.90 | 0.41 – 2.16 | 0.802 |
| Very good | 0.70 | 0.40 – 1.27 | 0.219 | 0.63 | 0.29 – 1.52 | 0.278 | 0.66 | 0.29 – 1.65 | 0.353 |
| Excellent | 0.53 | 0.28 – 1.03 | 0.056 | 0.44 | 0.19 – 1.13 | 0.075 | 0.47 | 0.14 – 1.48 | 0.202 |
| **ADG Score** |  |  |  |  |  |  |  |  |  |
| < 3 | Reference | |  |  |  |  |  |  |  |
| 3 – 4 | 0.88 | 0.57 – 1.38 | 0.572 | 1.10 | 0.66 – 1.87 | 0.725 | 0.49 | 0.20 – 1.26 | 0.123 |
| 5 – 6 | 0.70 | 0.45 – 1.11 | 0.128 | 0.84 | 0.49 – 1.46 | 0.520 | 0.54 | 0.23 – 1.35 | 0.168 |
| 7 – 8 | 0.81 | 0.52 – 1.29 | 0.363 | 1.24 | 0.71 – 2.19 | 0.450 | 0.47 | 0.20 – 1.18 | 0.094 |
| ≥ 9 | 0.73 | 0.46 – 1.17 | 0.183 | 0.82 | 0.45 – 1.50 | 0.523 | 0.72 | 0.32 – 1.76 | 0.442 |
| **Program type** |  |  |  |  |  |  |  |  |  |
| Enhanced FFS | Reference | |  |  |  |  |  |  |  |
| Capitation | 0.76 | 0.57 – 1.00 | 0.051 | 0.66 | 0.47 – 0.95 | **0.023** | 1.01 | 0.60 – 1.75 | 0.969 |
| Other | 0.80 | 0.26 – 1.98 | 0.654 | 0.84 | 0.18 – 2.76 | 0.793 | 0.92 | 0.13 – 3.82 | 0.916 |
| **Years with provider** |  |  |  |  |  |  |  |  |  |
| < 4 | Reference | |  |  |  |  |  |  |  |
| 4 – 9 | 0.61 | 0.43 – 0.84 | **0.003** | 0.66 | 0.44 – 0.99 | **0.044** | 0.45 | 0.23 – 0.83 | **0.013** |
| 10 – 19 | 0.61 | 0.43 – 0.84 | **0.003** | 0.53 | 0.34 – 0.82 | **0.004** | 0.76 | 0.43 – 1.31 | 0.324 |
| ≥ 20 | 0.45 | 0.32 – 0.63 | **<0.001** | 0.48 | 0.31 – 0.75 | **0.001** | 0.40 | 0.23 – 0.70 | **0.001** |
| **RIO category** |  |  |  |  |  |  |  |  |  |
| Large urban | Reference | |  |  |  |  |  |  |  |
| Urban | 0.91 | 0.67 – 1.23 | 0.531 | 0.85 | 0.59 – 1.23 | 0.391 | 0.87 | 0.48 – 1.54 | 0.630 |
| Small urban | 0.92 | 0.62 – 1.37 | 0.695 | 0.78 | 0.46 – 1.29 | 0.333 | 1.27 | 0.66 – 2.46 | 0.477 |
| Rural | 0.89 | 0.50 – 1.56 | 0.697 | 0.54 | 0.23 – 1.18 | 0.136 | 1.71 | 0.71 – 4.04 | 0.221 |
| **Dependency** |  |  |  |  |  |  |  |  |  |
| 1^st^ quintile *(least marginalized)* | Reference | |  |  |  |  |  |  |  |
| 2^nd^ quintile | 1.00 | 0.69 – 1.45 | 0.995 | 0.98 | 0.63 – 1.51 | 0.921 | 1.52 | 0.69 – 3.52 | 0.315 |
| 3^rd^ quintile | 0.85 | 0.57 – 1.26 | 0.411 | 0.78 | 0.48 – 1.26 | 0.320 | 1.32 | 0.58 – 3.13 | 0.513 |
| 4^th^ quintile | 1.06 | 0.71 – 1.58 | 0.780 | 1.12 | 0.68 – 1.82 | 0.654 | 1.44 | 0.64 – 3.39 | 0.391 |
| 5^th^ quintile *(most marginalized)* | 0.73 | 0.47 – 1.12 | 0.145 | 0.60 | 0.32 – 1.09 | 0.102 | 1.51 | 0.69 – 3.51 | 0.312 |
| **Material Deprivation** |  |  |  |  |  |  |  |  |  |
| 1^st^ quintile *(least marginalized)* | Reference | |  |  |  |  |  |  |  |
| 2^nd^ quintile | 1.22 | 0.85 – 1.75 | 0.288 | 1.19 | 0.76 – 1.86 | 0.453 | 1.45 | 0.77 – 2.74 | 0.253 |
| 3^rd^ quintile | 1.18 | 0.80 – 1.74 | 0.404 | 1.14 | 0.69 – 1.86 | 0.615 | 1.29 | 0.67 – 2.50 | 0.449 |
| 4^th^ quintile | 1.14 | 0.75 – 1.75 | 0.532 | 1.29 | 0.74 – 2.21 | 0.362 | 1.14 | 0.56 – 2.32 | 0.712 |
| 5^th^ quintile *(most marginalized)* | 1.49 | 0.96 – 2.32 | 0.077 | 2.03 | 1.17 – 3.55 | **0.012** | 0.93 | 0.41 – 2.05 | 0.852 |
| **Ethnic Concentration** |  |  |  |  |  |  |  |  |  |
| 1^st^ quintile *(least marginalized)* | Reference | |  |  |  |  |  |  |  |
| 2^nd^ quintile | 1.34 | 0.87 – 2.06 | 0.185 | 1.64 | 0.92 – 2.96 | 0.094 | 1.32 | 0.67 – 2.59 | 0.418 |
| 3^rd^ quintile | 1.21 | 0.75 – 1.93 | 0.435 | 1.17 | 0.62 – 2.22 | 0.624 | 1.48 | 0.72 – 3.07 | 0.288 |
| 4^th^ quintile | 1.26 | 0.77 – 2.09 | 0.367 | 0.98 | 0.51 – 1.91 | 0.957 | 2.13 | 0.93 – 4.97 | 0.076 |
| 5^th^ quintile *(most marginalized)* | 1.61 | 0.96 – 2.73 | 0.076 | 1.27 | 0.65 – 2.52 | 0.488 | 2.29 | 0.92 – 5.77 | 0.077 |
| **Residential Instability** |  |  |  |  |  |  |  |  |  |
| 1^st^ quintile *(least marginalized)* | Reference | |  |  |  |  |  |  |  |
| 2^nd^ quintile | 1.41 | 0.96 – 2.09 | 0.084 | 1.13 | 0.69 – 1.84 | 0.619 | 2.11 | 1.04 – 4.43 | **0.042** |
| 3^rd^ quintile | 1.13 | 0.74 – 1.72 | 0.573 | 1.39 | 0.84 – 2.30 | 0.197 | 0.75 | 0.32 – 1.72 | 0.492 |
| 4^th^ quintile | 1.45 | 0.95 – 2.21 | 0.082 | 0.96 | 0.56 – 1.65 | 0.889 | 2.46 | 1.21 – 5.21 | **0.015** |
| 5^th^ quintile *(most marginalized)* | 1.09 | 0.71 – 1.67 | 0.696 | 1.17 | 0.70 – 1.96 | 0.541 | 0.97 | 0.43 – 2.20 | 0.937 |

Associations between factors and primary care provider sometimes, rarely, or never knowing medical history, stratified by number of encounters over past 12 months.

| **Predictors** | **Full Model, N = 2,677** | |  | **≤ 3 encounters, N = 1,648** | |  | **> 3 encounters, N = 1,039** | |  |
| --- | --- | --- | --- | --- | --- | --- | --- | --- | --- |
|  | **Odds Ratios** | **CI (95%)** | **p** | **Odds Ratios** | **CI (95%)** | **p** | **Odds Ratios** | **CI (95%)** | **p** |
| **Telehealth access** | 0.91 | 0.71 – 1.17 | 0.469 | 1.01 | 0.74 – 1.39 | 0.961 | 0.82 | 0.53 – 1.27 | 0.372 |
| **Health record access** | 0.35 | 0.11 – 0.88 | **0.048** | 0.29 | 0.05 – 1.01 | 0.099 | 0.42 | 0.07 – 1.49 | 0.251 |
| **Online booking access** | 1.33 | 0.69 – 2.38 | 0.363 | 1.36 | 0.63 – 2.70 | 0.401 | 0.91 | 0.20 – 2.88 | 0.888 |
| **Sex** |  |  |  |  |  |  |  |  |  |
| Female | Reference | |  |  |  |  |  |  |  |
| Male | 0.85 | 0.66 – 1.09 | 0.194 | 0.80 | 0.58 – 1.09 | 0.160 | 0.97 | 0.62 – 1.49 | 0.873 |
| **Financial Situation** |  |  |  |  |  |  |  |  |  |
| Very comfortable | Reference | |  |  |  |  |  |  |  |
| Comfortable | 1.32 | 0.93 – 1.90 | 0.128 | 1.58 | 1.01 – 2.56 | 0.055 | 1.01 | 0.57 – 1.84 | 0.978 |
| Tight/Very tight/Poor | 1.50 | 0.99 – 2.29 | 0.059 | 1.89 | 1.10 – 3.30 | **0.024** | 0.97 | 0.49 – 1.96 | 0.938 |
| **Educational Attainment** |  |  |  |  |  |  |  |  |  |
| High school or less | Reference | |  |  |  |  |  |  |  |
| Some college/university | 0.88 | 0.51 – 1.46 | 0.622 | 0.76 | 0.36 – 1.50 | 0.451 | 0.98 | 0.40 – 2.20 | 0.967 |
| Completed college/university | 1.41 | 1.04 – 1.93 | **0.028** | 1.63 | 1.10 – 2.46 | **0.016** | 1.18 | 0.71 – 1.98 | 0.530 |
| Post-graduate/professional degree | 1.30 | 0.85 – 2.00 | 0.225 | 1.30 | 0.74 – 2.28 | 0.360 | 1.38 | 0.67 – 2.79 | 0.370 |
| **Primary language spoken** |  |  |  |  |  |  |  |  |  |
| English | Reference | |  |  |  |  |  |  |  |
| Other | 1.55 | 1.10 – 2.16 | **0.011** | 1.19 | 0.74 – 1.87 | 0.450 | 2.20 | 1.28 – 3.72 | **0.004** |
| **Self-perceived health** |  |  |  |  |  |  |  |  |  |
| Poor | Reference | |  |  |  |  |  |  |  |
| Fair | 0.77 | 0.43 – 1.43 | 0.392 | 0.55 | 0.23 – 1.40 | 0.191 | 1.01 | 0.44 – 2.49 | 0.977 |
| Good | 0.82 | 0.47 – 1.48 | 0.485 | 0.65 | 0.30 – 1.56 | 0.307 | 0.90 | 0.40 – 2.16 | 0.798 |
| Very good | 0.70 | 0.40 – 1.27 | 0.219 | 0.55 | 0.25 – 1.31 | 0.153 | 0.63 | 0.26 – 1.61 | 0.312 |
| Excellent | 0.53 | 0.28 – 1.03 | 0.056 | 0.30 | 0.12 – 0.76 | **0.008** | 1.17 | 0.42 – 3.37 | 0.764 |
| **ADG Score** |  |  |  |  |  |  |  |  |  |
| < 3 | Reference | |  |  |  |  |  |  |  |
| 3 – 4 | 0.88 | 0.57 – 1.38 | 0.572 | 1.06 | 0.66 – 1.73 | 0.831 | 0.19 | 0.05 – 0.70 | **0.012** |
| 5 – 6 | 0.70 | 0.45 – 1.11 | 0.128 | 0.79 | 0.47 – 1.33 | 0.814 | 0.30 | 0.10 – 0.96 | **0.032** |
| 7 – 8 | 0.81 | 0.52 – 1.29 | 0.363 | 0.99 | 0.57 – 1.72 | 0.362 | 0.37 | 0.13 – 1.16 | 0.071 |
| ≥ 9 | 0.73 | 0.46 – 1.17 | 0.183 | 1.07 | 0.58 – 1.94 | 0.973 | 0.32 | 0.12 – 1.00 | **0.037** |
| **Program type** |  |  |  |  |  |  |  |  |  |
| Enhanced FFS | Reference | |  |  |  |  |  |  |  |
| Capitation | 0.76 | 0.57 – 1.00 | 0.051 | 0.74 | 0.51 – 1.10 | 0.133 | 0.63 | 0.40 – 1.00 | 0.050 |
| Other | 0.80 | 0.26 – 1.98 | 0.654 | 0.61 | 0.13 – 1.99 | 0.458 | 1.19 | 0.17 – 5.01 | 0.833 |
| **Years with provider** |  |  |  |  |  |  |  |  |  |
| < 4 | Reference | |  |  |  |  |  |  |  |
| 4 – 9 | 0.61 | 0.43 – 0.84 | **0.003** | 0.66 | 0.44 – 0.99 | **0.044** | 0.47 | 0.25 – 0.86 | **0.015** |
| 10 – 19 | 0.61 | 0.43 – 0.84 | **0.003** | 0.56 | 0.37 – 0.86 | **0.008** | 0.69 | 0.39 – 1.23 | 0.208 |
| ≥ 20 | 0.45 | 0.32 – 0.63 | **<0.001** | 0.42 | 0.28 – 0.65 | **<0.001** | 0.49 | 0.27 – 0.86 | **0.014** |
| **RIO category** |  |  |  |  |  |  |  |  |  |
| Large urban | Reference | |  |  |  |  |  |  |  |
| Urban | 0.91 | 0.67 – 1.23 | 0.531 | 0.94 | 0.64 – 1.37 | 0.755 | 0.83 | 0.49 – 1.38 | 0.481 |
| Small urban | 0.92 | 0.62 – 1.37 | 0.695 | 0.92 | 0.57 – 1.47 | 0.718 | 0.88 | 0.42 – 1.79 | 0.728 |
| Rural | 0.89 | 0.50 – 1.56 | 0.697 | 0.95 | 0.48 – 1.82 | 0.872 | 0.71 | 0.22 – 2.04 | 0.542 |
| **Dependency** |  |  |  |  |  |  |  |  |  |
| 1^st^ quintile *(least marginalized)* | Reference | |  |  |  |  |  |  |  |
| 2^nd^ quintile | 1.00 | 0.69 – 1.45 | 0.995 | 1.45 | 0.92 – 2.31 | 0.110 | 0.58 | 0.29 – 1.13 | 0.112 |
| 3^rd^ quintile | 0.85 | 0.57 – 1.26 | 0.411 | 0.81 | 0.49 – 1.34 | 0.411 | 0.98 | 0.50 – 1.90 | 0.949 |
| 4^th^ quintile | 1.06 | 0.71 – 1.58 | 0.780 | 1.17 | 0.70 – 1.96 | 0.537 | 0.96 | 0.48 – 1.89 | 0.900 |
| 5^th^ quintile *(most marginalized)* | 0.73 | 0.47 – 1.12 | 0.145 | 0.83 | 0.47 – 1.43 | 0.495 | 0.65 | 0.32 – 1.32 | 0.236 |
| **Material Deprivation** |  |  |  |  |  |  |  |  |  |
| 1^st^ quintile *(least marginalized)* | Reference | |  |  |  |  |  |  |  |
| 2^nd^ quintile | 1.22 | 0.85 – 1.75 | 0.288 | 1.23 | 0.79 – 1.91 | 0.354 | 1.32 | 0.69 – 2.56 | 0.407 |
| 3^rd^ quintile | 1.18 | 0.80 – 1.74 | 0.404 | 1.19 | 0.74 – 1.92 | 0.473 | 1.16 | 0.57 – 2.36 | 0.681 |
| 4^th^ quintile | 1.14 | 0.75 – 1.75 | 0.532 | 1.13 | 0.66 – 1.92 | 0.650 | 1.12 | 0.54 – 2.32 | 0.763 |
| 5^th^ quintile *(most marginalized)* | 1.49 | 0.96 – 2.32 | 0.077 | 1.50 | 0.85 – 2.63 | 0.160 | 1.76 | 0.84 – 3.74 | 0.139 |
| **Ethnic Concentration** |  |  |  |  |  |  |  |  |  |
| 1^st^ quintile *(least marginalized)* | Reference | |  |  |  |  |  |  |  |
| 2^nd^ quintile | 1.34 | 0.87 – 2.06 | 0.185 | 1.63 | 0.98 – 2.73 | 0.063 | 0.91 | 0.40 – 2.06 | 0.816 |
| 3^rd^ quintile | 1.21 | 0.75 – 1.93 | 0.435 | 1.46 | 0.83 – 2.59 | 0.186 | 0.92 | 0.40 – 2.14 | 0.837 |
| 4^th^ quintile | 1.26 | 0.77 – 2.09 | 0.367 | 1.15 | 0.61 – 2.17 | 0.662 | 1.33 | 0.57 – 3.20 | 0.518 |
| 5^th^ quintile *(most marginalized)* | 1.61 | 0.96 – 2.73 | 0.076 | 1.94 | 1.02 – 3.73 | **0.046** | 1.15 | 0.47 – 2.94 | 0.764 |
| **Residential Instability** |  |  |  |  |  |  |  |  |  |
| 1^st^ quintile *(least marginalized)* | Reference | |  |  |  |  |  |  |  |
| 2^nd^ quintile | 1.41 | 0.96 – 2.09 | 0.084 | 1.19 | 0.73 – 1.93 | 0.481 | 1.96 | 0.98 – 3.99 | 0.061 |
| 3^rd^ quintile | 1.13 | 0.74 – 1.72 | 0.573 | 1.06 | 0.63 – 1.76 | 0.835 | 1.32 | 0.62 – 2.81 | 0.474 |
| 4^th^ quintile | 1.45 | 0.95 – 2.21 | 0.082 | 1.41 | 0.84 – 2.39 | 0.195 | 1.72 | 0.84 – 3.59 | 0.141 |
| 5^th^ quintile *(most marginalized)* | 1.09 | 0.71 – 1.67 | 0.696 | 1.02 | 0.60 – 1.75 | 0.934 | 1.20 | 0.59 – 2.51 | 0.614 |

Associations between factors and primary care provider sometimes, rarely, or never giving the patient opportunity to ask questions, stratified by patient age.

| **Predictors** | **Full Model, N = 2,677** | |  | **Age ≤ 60 years, N = 1,378** | |  | **Age > 60 years, N = 1,308** | |  |
| --- | --- | --- | --- | --- | --- | --- | --- | --- | --- |
|  | **Odds Ratios** | **CI (95%)** | **p** | **Odds Ratios** | **CI (95%)** | **p** | **Odds Ratios** | **CI (95%)** | **p** |
| **Telehealth access** | 0.88 | 0.68 – 1.14 | 0.322 | 0.91 | 0.65 – 1.28 | 0.589 | 0.72 | 0.48 – 1.10 | 0.124 |
| **Health record access** | 0.41 | 0.12 – 1.02 | 0.091 | 0.42 | 0.10 – 1.24 | 0.170 | 0.42 | 0.02 – 2.20 | 0.411 |
| **Online booking access** | 1.07 | 0.50 – 2.03 | 0.855 | 1.05 | 0.46 – 2.16 | 0.894 | 0.52 | 0.03 – 2.75 | 0.537 |
| **Sex** |  |  |  |  |  |  |  |  |  |
| Female | Reference | |  |  |  |  |  |  |  |
| Male | 0.85 | 0.65 – 1.09 | 0.200 | 1.01 | 0.72 – 1.42 | 0.935 | 0.70 | 0.45 – 1.07 | 0.101 |
| **Financial Situation** |  |  |  |  |  |  |  |  |  |
| Very comfortable | Reference | |  |  |  |  |  |  |  |
| Comfortable | 1.00 | 0.72 – 1.42 | 0.997 | 0.83 | 0.53 – 1.33 | 0.418 | 0.96 | 0.57 – 1.68 | 0.885 |
| Tight/Very tight/Poor | 1.38 | 0.93 – 2.08 | 0.114 | 1.31 | 0.78 – 2.25 | 0.313 | 1.21 | 0.62 – 2.39 | 0.580 |
| **Educational Attainment** |  |  |  |  |  |  |  |  |  |
| High school or less | Reference | |  |  |  |  |  |  |  |
| Some college/university | 0.75 | 0.44 – 1.22 | 0.261 | 0.47 | 0.18 – 1.06 | 0.087 | 1.17 | 0.60 – 2.18 | 0.622 |
| Completed college/university | 0.92 | 0.69 – 1.24 | 0.596 | 1.23 | 0.79 – 1.96 | 0.366 | 0.64 | 0.40 – 1.01 | 0.055 |
| Post-graduate/professional degree | 0.78 | 0.50 – 1.20 | 0.265 | 1.07 | 0.60 – 1.93 | 0.812 | 0.55 | 0.23 – 1.19 | 0.154 |
| **Primary language spoken** |  |  |  |  |  |  |  |  |  |
| English | Reference | |  |  |  |  |  |  |  |
| Other | 1.54 | 1.08 – 2.15 | **0.014** | 1.29 | 0.85 – 1.95 | 0.225 | 1.80 | 0.90 – 3.42 | 0.081 |
| **Self-perceived health** |  |  |  |  |  |  |  |  |  |
| Poor | Reference | |  |  |  |  |  |  |  |
| Fair | 1.36 | 0.74 – 2.62 | 0.343 | 1.81 | 0.71 – 5.32 | 0.241 | 0.95 | 0.42 – 2.33 | 0.909 |
| Good | 1.21 | 0.68 – 2.29 | 0.530 | 0.90 | 0.37 – 2.54 | 0.825 | 1.50 | 0.70 – 3.55 | 0.325 |
| Very good | 1.17 | 0.65 – 2.23 | 0.617 | 1.16 | 0.48 – 3.28 | 0.758 | 0.85 | 0.36 – 2.11 | 0.706 |
| Excellent | 1.01 | 0.52 – 2.05 | 0.975 | 0.78 | 0.30 – 2.30 | 0.621 | 1.11 | 0.37 – 3.32 | 0.853 |
| **ADG Score** |  |  |  |  |  |  |  |  |  |
| < 3 | Reference | |  |  |  |  |  |  |  |
| 3 – 4 | 0.94 | 0.60 – 1.52 | 0.807 | 1.04 | 0.62 – 1.81 | 0.872 | 0.67 | 0.26 – 1.84 | 0.405 |
| 5 – 6 | 0.77 | 0.48 – 1.24 | 0.269 | 0.89 | 0.51 – 1.56 | 0.671 | 0.56 | 0.22 – 1.53 | 0.230 |
| 7 – 8 | 0.81 | 0.50 – 1.33 | 0.402 | 0.75 | 0.41 – 1.39 | 0.359 | 0.88 | 0.36 – 2.35 | 0.777 |
| ≥ 9 | 1.09 | 0.69 – 1.77 | 0.709 | 0.97 | 0.53 – 1.78 | 0.911 | 1.33 | 0.58 – 3.51 | 0.527 |
| **Program type** |  |  |  |  |  |  |  |  |  |
| Enhanced FFS | Reference | |  |  |  |  |  |  |  |
| Capitation | 0.78 | 0.59 – 1.03 | 0.081 | 0.76 | 0.53 – 1.09 | 0.131 | 0.84 | 0.52 – 1.38 | 0.483 |
| Other | 0.64 | 0.18 – 1.67 | 0.410 | 0.25 | 0.01 – 1.38 | 0.199 | 0.86 | 0.18 – 2.98 | 0.832 |
| **Years with provider** |  |  |  |  |  |  |  |  |  |
| < 4 | Reference | |  |  |  |  |  |  |  |
| 4 – 9 | 1.06 | 0.74 – 1.52 | 0.766 | 1.19 | 0.76 – 1.89 | 0.448 | 0.77 | 0.40 – 1.44 | 0.411 |
| 10 – 19 | 1.06 | 0.74 – 1.53 | 0.738 | 1.12 | 0.70 – 1.81 | 0.634 | 0.90 | 0.49 – 1.62 | 0.715 |
| ≥ 20 | 1.05 | 0.75 – 1.49 | 0.772 | 1.29 | 0.81 – 2.07 | 0.290 | 0.91 | 0.53 – 1.56 | 0.718 |
| **RIO category** |  |  |  |  |  |  |  |  |  |
| Large urban | Reference | |  |  |  |  |  |  |  |
| Urban | 1.28 | 0.95 – 1.73 | 0.109 | 1.13 | 0.76 – 1.65 | 0.548 | 1.37 | 0.80 – 2.32 | 0.247 |
| Small urban | 1.04 | 0.70 – 1.55 | 0.841 | 1.23 | 0.71 – 2.09 | 0.456 | 0.93 | 0.49 – 1.77 | 0.834 |
| Rural | 0.86 | 0.47 – 1.52 | 0.612 | 0.64 | 0.25 – 1.50 | 0.331 | 1.11 | 0.46 – 2.59 | 0.806 |
| **Dependency** |  |  |  |  |  |  |  |  |  |
| 1^st^ quintile *(least marginalized)* | Reference | |  |  |  |  |  |  |  |
| 2^nd^ quintile | 0.97 | 0.66 – 1.42 | 0.889 | 1.00 | 0.64 – 1.57 | 0.994 | 1.55 | 0.67 – 3.80 | 0.317 |
| 3^rd^ quintile | 1.07 | 0.73 – 1.58 | 0.730 | 0.89 | 0.54 – 1.43 | 0.622 | 2.15 | 0.97 – 5.21 | 0.072 |
| 4^th^ quintile | 0.78 | 0.50 – 1.19 | 0.254 | 0.69 | 0.40 – 1.19 | 0.189 | 1.45 | 0.63 – 3.61 | 0.401 |
| 5^th^ quintile *(most marginalized)* | 0.62 | 0.40 – 0.96 | **0.032** | 0.48 | 0.25 – 0.90 | **0.026** | 1.35 | 0.60 – 3.29 | 0.482 |
| **Material Deprivation** |  |  |  |  |  |  |  |  |  |
| 1^st^ quintile *(least marginalized)* | Reference | |  |  |  |  |  |  |  |
| 2^nd^ quintile | 1.19 | 0.82 – 1.73 | 0.354 | 1.03 | 0.64 – 1.64 | 0.907 | 1.49 | 0.79 – 2.86 | 0.218 |
| 3^rd^ quintile | 1.18 | 0.80 – 1.76 | 0.406 | 0.87 | 0.51 – 1.47 | 0.609 | 1.75 | 0.92 – 3.38 | 0.091 |
| 4^th^ quintile | 1.34 | 0.88 – 2.04 | 0.171 | 1.59 | 0.93 – 2.72 | 0.091 | 1.17 | 0.57 – 2.40 | 0.671 |
| 5^th^ quintile *(most marginalized)* | 1.05 | 0.66 – 1.67 | 0.831 | 1.13 | 0.62 – 2.05 | 0.686 | 1.19 | 0.54 – 2.61 | 0.655 |
| **Ethnic Concentration** |  |  |  |  |  |  |  |  |  |
| 1^st^ quintile *(least marginalized)* | Reference | |  |  |  |  |  |  |  |
| 2^nd^ quintile | 1.01 | 0.66 – 1.53 | 0.973 | 1.24 | 0.69 – 2.26 | 0.475 | 0.89 | 0.48 – 1.67 | 0.725 |
| 3^rd^ quintile | 0.77 | 0.48 – 1.22 | 0.265 | 0.63 | 0.32 – 1.24 | 0.183 | 0.98 | 0.51 – 1.90 | 0.959 |
| 4^th^ quintile | 0.88 | 0.54 – 1.45 | 0.624 | 0.91 | 0.47 – 1.79 | 0.786 | 0.72 | 0.32 – 1.60 | 0.421 |
| 5^th^ quintile *(most marginalized)* | 1.19 | 0.72 – 1.99 | 0.509 | 1.18 | 0.60 – 2.37 | 0.645 | 1.05 | 0.44 – 2.46 | 0.920 |
| **Residential Instability** |  |  |  |  |  |  |  |  |  |
| 1^st^ quintile *(least marginalized)* | Reference | |  |  |  |  |  |  |  |
| 2^nd^ quintile | 1.13 | 0.75 – 1.70 | 0.547 | 1.08 | 0.65 – 1.77 | 0.775 | 1.27 | 0.61 – 2.74 | 0.527 |
| 3^rd^ quintile | 1.11 | 0.73 – 1.69 | 0.610 | 1.05 | 0.61 – 1.77 | 0.869 | 1.25 | 0.60 – 2.68 | 0.553 |
| 4^th^ quintile | 1.26 | 0.82 – 1.93 | 0.293 | 1.02 | 0.58 – 1.78 | 0.946 | 1.64 | 0.80 – 3.49 | 0.183 |
| 5^th^ quintile *(most marginalized)* | 1.35 | 0.88 – 2.06 | 0.166 | 1.47 | 0.87 – 2.50 | 0.146 | 1.20 | 0.56 – 2.67 | 0.646 |

Associations between factors and primary care provider sometimes, rarely, or never giving the patient opportunity to ask questions, stratified by number of encounters over past 12 months.

| **Predictors** | **Full Model, N = 2,677** | |  | **≤ 3 encounters, N = 1,648** | |  | **> 3 encounters, N = 1,039** | |  |
| --- | --- | --- | --- | --- | --- | --- | --- | --- | --- |
|  | **Odds Ratios** | **CI (95%)** | **p** | **Odds Ratios** | **CI (95%)** | **p** | **Odds Ratios** | **CI (95%)** | **p** |
| **Telehealth access** | 0.88 | 0.68 – 1.14 | 0.322 | 1.15 | 0.83 – 1.61 | 0.412 | 0.63 | 0.41 – 0.96* | 0.033 |
| **Health record access** | 0.41 | 0.12 – 1.02 | 0.091 | 0.20 | 0.01 – 0.99 | 0.123 | 0.83 | 0.19 – 2.56 | 0.775 |
| **Online booking access** | 1.07 | 0.50 – 2.03 | 0.855 | 1.25 | 0.52 – 2.65 | 0.588 | 0.53 | 0.08 – 1.92 | 0.404 |
| **Sex** |  |  |  |  |  |  |  |  |  |
| Female | Reference | |  |  |  |  |  |  |  |
| Male | 0.85 | 0.65 – 1.09 | 0.200 | 0.96 | 0.69 – 1.33 | 0.803 | 0.74 | 0.47 – 1.15 | 0.184 |
| **Financial Situation** |  |  |  |  |  |  |  |  |  |
| Very comfortable | Reference | |  |  |  |  |  |  |  |
| Comfortable | 1.00 | 0.72 – 1.42 | 0.997 | 1.01 | 0.66 – 1.59 | 0.962 | 1.11 | 0.63 – 2.01 | 0.733 |
| Tight/Very tight/Poor | 1.38 | 0.93 – 2.08 | 0.114 | 1.47 | 0.87 – 2.51 | 0.154 | 1.55 | 0.80 – 3.09 | 0.198 |
| **Educational Attainment** |  |  |  |  |  |  |  |  |  |
| High school or less | Reference | |  |  |  |  |  |  |  |
| Some college/university | 0.75 | 0.44 – 1.22 | 0.261 | 0.54 | 0.24 – 1.08 | 0.097 | 1.03 | 0.47 – 2.12 | 0.944 |
| Completed college/university | 0.92 | 0.69 – 1.24 | 0.596 | 1.06 | 0.72 – 1.57 | 0.787 | 0.75 | 0.46 – 1.21 | 0.239 |
| Post-graduate/professional degree | 0.78 | 0.50 – 1.20 | 0.265 | 0.72 | 0.39 – 1.29 | 0.281 | 1.10 | 0.53 – 2.18 | 0.799 |
| **Primary language spoken** |  |  |  |  |  |  |  |  |  |
| English | Reference | |  |  |  |  |  |  |  |
| Other | 1.54 | 1.08 – 2.15 | **0.014** | 1.60 | 1.00 – 2.51 | **0.046** | 1.60 | 0.91 – 2.76 | 0.099 |
| **Self-perceived health** |  |  |  |  |  |  |  |  |  |
| Poor | Reference | |  |  |  |  |  |  |  |
| Fair | 1.36 | 0.74 – 2.62 | 0.343 | 1.38 | 0.54 – 4.01 | 0.525 | 1.21 | 0.53 – 2.97 | 0.658 |
| Good | 1.21 | 0.68 – 2.29 | 0.530 | 0.85 | 0.35 – 2.39 | 0.732 | 1.59 | 0.73 – 3.79 | 0.262 |
| Very good | 1.17 | 0.65 – 2.23 | 0.617 | 1.00 | 0.42 – 2.80 | 0.998 | 1.07 | 0.45 – 2.70 | 0.888 |
| Excellent | 1.01 | 0.52 – 2.05 | 0.975 | 0.76 | 0.29 – 2.24 | 0.590 | 1.23 | 0.40 – 3.73 | 0.711 |
| **ADG Score** |  |  |  |  |  |  |  |  |  |
| < 3 | Reference | |  |  |  |  |  |  |  |
| 3 – 4 | 0.94 | 0.60 – 1.52 | 0.807 | 1.17 | 0.71 – 1.97 | 0.545 | 0.25 | 0.06 – 1.01 | **0.046** |
| 5 – 6 | 0.77 | 0.48 – 1.24 | 0.269 | 0.82 | 0.48 – 1.43 | 0.481 | 0.52 | 0.17 – 1.82 | 0.270 |
| 7 – 8 | 0.81 | 0.50 – 1.33 | 0.402 | 1.12 | 0.63 – 2.02 | 0.696 | 0.40 | 0.13 – 1.38 | 0.118 |
| ≥ 9 | 1.09 | 0.69 – 1.77 | 0.709 | 1.32 | 0.71 – 2.47 | 0.382 | 0.70 | 0.24 – 2.35 | 0.527 |
| **Program type** |  |  |  |  |  |  |  |  |  |
| Enhanced FFS | Reference | |  |  |  |  |  |  |  |
| Capitation | 0.78 | 0.59 – 1.03 | 0.081 | 0.74 | 0.50 – 1.11 | 0.143 | 0.74 | 0.47 – 1.15 | 0.180 |
| Other | 0.64 | 0.18 – 1.67 | 0.410 | 0.50 | 0.08 – 1.87 | 0.368 | 0.80 | 0.12 – 3.28 | 0.778 |
| **Years with provider** |  |  |  |  |  |  |  |  |  |
| < 4 | Reference | |  |  |  |  |  |  |  |
| 4 – 9 | 1.06 | 0.74 – 1.52 | 0.766 | 0.73 | 0.47 – 1.14 | 0.166 | 2.39 | 1.18 – 5.10 | **0.019** |
| 10 – 19 | 1.06 | 0.74 – 1.53 | 0.738 | 0.76 | 0.48 – 1.19 | 0.227 | 2.26 | 1.12 – 4.81 | **0.027** |
| ≥ 20 | 1.05 | 0.75 – 1.49 | 0.772 | 0.69 | 0.44 – 1.06 | 0.091 | 2.65 | 1.36 – 5.53 | **0.006** |
| **RIO category** |  |  |  |  |  |  |  |  |  |
| Large urban | Reference | |  |  |  |  |  |  |  |
| Urban | 1.28 | 0.95 – 1.73 | 0.109 | 1.12 | 0.76 – 1.66 | 0.567 | 1.53 | 0.93 – 2.52 | 0.096 |
| Small urban | 1.04 | 0.70 – 1.55 | 0.841 | 0.88 | 0.53 – 1.47 | 0.632 | 1.20 | 0.60 – 2.38 | 0.596 |
| Rural | 0.86 | 0.47 – 1.52 | 0.612 | 0.81 | 0.39 – 1.63 | 0.563 | 0.87 | 0.27 – 2.46 | 0.796 |
| **Dependency** |  |  |  |  |  |  |  |  |  |
| 1^st^ quintile *(least marginalized)* | Reference | |  |  |  |  |  |  |  |
| 2^nd^ quintile | 0.97 | 0.66 – 1.42 | 0.889 | 1.01 | 0.63 – 1.61 | 0.972 | 1.14 | 0.56 – 2.31 | 0.720 |
| 3^rd^ quintile | 1.07 | 0.73 – 1.58 | 0.730 | 0.72 | 0.43 – 1.18 | 0.188 | 2.38 | 1.21 – 4.74 | **0.012** |
| 4^th^ quintile | 0.78 | 0.50 – 1.19 | 0.254 | 0.64 | 0.37 – 1.09 | 0.105 | 1.24 | 0.59 – 2.64 | 0.567 |
| 5^th^ quintile *(most marginalized)* | 0.62 | 0.40 – 0.96 | **0.032** | 0.37 | 0.20 – 0.67 | **0.001** | 1.33 | 0.65 – 2.78 | 0.439 |
| **Material Deprivation** |  |  |  |  |  |  |  |  |  |
| 1^st^ quintile *(least marginalized)* | Reference | |  |  |  |  |  |  |  |
| 2^nd^ quintile | 1.19 | 0.82 – 1.73 | 0.354 | 0.93 | 0.58 – 1.49 | 0.766 | 1.73 | 0.91 – 3.41 | 0.102 |
| 3^rd^ quintile | 1.18 | 0.80 – 1.76 | 0.406 | 1.27 | 0.78 – 2.06 | 0.332 | 0.98 | 0.47 – 2.06 | 0.957 |
| 4^th^ quintile | 1.34 | 0.88 – 2.04 | 0.171 | 1.49 | 0.88 – 2.52 | 0.140 | 1.11 | 0.53 – 2.36 | 0.785 |
| 5^th^ quintile *(most marginalized)* | 1.05 | 0.66 – 1.67 | 0.831 | 0.77 | 0.40 – 1.44 | 0.417 | 1.53 | 0.72 – 3.32 | 0.272 |
| **Ethnic Concentration** |  |  |  |  |  |  |  |  |  |
| 1^st^ quintile *(least marginalized)* | Reference | |  |  |  |  |  |  |  |
| 2^nd^ quintile | 1.01 | 0.66 – 1.53 | 0.973 | 1.08 | 0.64 – 1.82 | 0.784 | 0.83 | 0.39 – 1.73 | 0.614 |
| 3^rd^ quintile | 0.77 | 0.48 – 1.22 | 0.265 | 0.74 | 0.41 – 1.34 | 0.318 | 0.77 | 0.35 – 1.67 | 0.501 |
| 4^th^ quintile | 0.88 | 0.54 – 1.45 | 0.624 | 0.83 | 0.45 – 1.55 | 0.554 | 0.87 | 0.38 – 2.00 | 0.735 |
| 5^th^ quintile *(most marginalized)* | 1.19 | 0.72 – 1.99 | 0.509 | 1.05 | 0.55 – 2.03 | 0.889 | 1.38 | 0.59 – 3.32 | 0.460 |
| **Residential Instability** |  |  |  |  |  |  |  |  |  |
| 1^st^ quintile *(least marginalized)* | Reference | |  |  |  |  |  |  |  |
| 2^nd^ quintile | 1.13 | 0.75 – 1.70 | 0.547 | 1.34 | 0.82 – 2.21 | 0.247 | 0.82 | 0.38 – 1.73 | 0.598 |
| 3^rd^ quintile | 1.11 | 0.73 – 1.69 | 0.610 | 1.00 | 0.58 – 1.72 | 0.999 | 1.48 | 0.74 – 2.99 | 0.264 |
| 4^th^ quintile | 1.26 | 0.82 – 1.93 | 0.293 | 1.54 | 0.89 – 2.66 | 0.122 | 1.13 | 0.55 – 2.33 | 0.738 |
| 5^th^ quintile *(most marginalized)* | 1.35 | 0.88 – 2.06 | 0.166 | 1.23 | 0.70 – 2.14 | 0.470 | 1.36 | 0.68 – 2.76 | 0.383 |

Associations between factors and primary care provider sometimes, rarely, or never spending enough time with patient, stratified by age.

| **Predictors** | **Full Model, N = 2,677** | |  | **Age ≤ 60 years, N = 1,378** | |  | **Age > 60 years, N = 1,308** | |  |
| --- | --- | --- | --- | --- | --- | --- | --- | --- | --- |
|  | **Odds Ratios** | **CI (95%)** | **p** | **Odds Ratios** | **CI (95%)** | **p** | **Odds Ratios** | **CI (95%)** | **p** |
| **Telehealth access** | 0.89 | 0.71 – 1.13 | 0.335 | 0.97 | 0.72 – 1.30 | 0.820 | 0.67 | 0.46 – 0.98 | **0.039** |
| **Health record access** | 1.09 | 0.54 – 2.03 | 0.799 | 1.24 | 0.55 – 2.59 | 0.577 | 0.43 | 0.06 – 1.59 | 0.274 |
| **Online booking access** | 0.92 | 0.47 – 1.64 | 0.779 | 0.62 | 0.28 – 1.26 | 0.217 | 2.18 | 0.59 – 6.37 | 0.187 |
| **Sex** |  |  |  |  |  |  |  |  |  |
| Female | Reference | |  |  |  |  |  |  |  |
| Male | 0.84 | 0.66 – 1.06 | 0.136 | 0.91 | 0.67 – 1.23 | 0.539 | 0.80 | 0.53 – 1.17 | 0.254 |
| **Financial Situation** |  |  |  |  |  |  |  |  |  |
| Very comfortable | Reference | |  |  |  |  |  |  |  |
| Comfortable | 1.12 | 0.83 – 1.54 | 0.468 | 0.93 | 0.62 – 1.41 | 0.723 | 1.20 | 0.72 – 2.06 | 0.503 |
| Tight/Very tight/Poor | 1.53 | 1.06 – 2.22 | **0.025** | 1.22 | 0.76 – 1.96 | 0.414 | 1.45 | 0.76 – 2.80 | 0.256 |
| **Educational Attainment** |  |  |  |  |  |  |  |  |  |
| High school or less | Reference | |  |  |  |  |  |  |  |
| Some college/university | 0.77 | 0.48 – 1.21 | 0.276 | 0.72 | 0.38 – 1.35 | 0.319 | 0.73 | 0.34 – 1.43 | 0.378 |
| Completed college/university | 1.07 | 0.82 – 1.41 | 0.611 | 0.91 | 0.62 – 1.35 | 0.623 | 0.99 | 0.65 – 1.50 | 0.946 |
| Post-graduate/professional degree | 0.84 | 0.56 – 1.25 | 0.395 | 0.73 | 0.43 – 1.22 | 0.229 | 0.82 | 0.39 – 1.62 | 0.586 |
| **Primary language spoken** |  |  |  |  |  |  |  |  |  |
| English | Reference | |  |  |  |  |  |  |  |
| Other | 1.75 | 1.28 – 2.38 | **<0.001** | 1.62 | 1.12 – 2.34 | **0.010** | 1.47 | 0.75 – 2.71 | 0.236 |
| **Self-perceived health** |  |  |  |  |  |  |  |  |  |
| Poor | Reference | |  |  |  |  |  |  |  |
| Fair | 1.21 | 0.71 – 2.14 | 0.496 | 1.03 | 0.47 – 2.38 | 0.946 | 1.35 | 0.64 – 3.07 | 0.451 |
| Good | 1.05 | 0.63 – 1.82 | 0.851 | 0.80 | 0.38 – 1.76 | 0.557 | 1.15 | 0.56 – 2.60 | 0.712 |
| Very good | 0.88 | 0.52 – 1.54 | 0.642 | 0.69 | 0.33 – 1.55 | 0.349 | 0.79 | 0.36 – 1.85 | 0.564 |
| Excellent | 0.78 | 0.43 – 1.44 | 0.412 | 0.64 | 0.29 – 1.49 | 0.282 | 0.42 | 0.12 – 1.32 | 0.147 |
| **ADG Score** |  |  |  |  |  |  |  |  |  |
| < 3 | Reference | |  |  |  |  |  |  |  |
| 3 – 4 | 1.06 | 0.70 – 1.64 | 0.786 | 1.09 | 0.68 – 1.79 | 0.728 | 1.30 | 0.53 – 3.71 | 0.588 |
| 5 – 6 | 0.91 | 0.60 – 1.42 | 0.680 | 1.05 | 0.64 – 1.74 | 0.860 | 0.99 | 0.40 – 2.83 | 0.991 |
| 7 – 8 | 0.80 | 0.52 – 1.27 | 0.337 | 0.94 | 0.55 – 1.61 | 0.820 | 0.98 | 0.39 – 2.81 | 0.969 |
| ≥ 9 | 1.10 | 0.72 – 1.72 | 0.663 | 1.12 | 0.66 – 1.93 | 0.680 | 1.78 | 0.75 – 4.94 | 0.226 |
| **Program type** |  |  |  |  |  |  |  |  |  |
| Enhanced FFS | Reference | |  |  |  |  |  |  |  |
| Capitation | 0.75 | 0.59 – 0.98 | **0.031** | 0.74 | 0.54 – 1.02 | 0.068 | 0.94 | 0.60 – 1.52 | 0.809 |
| Other | 0.59 | 0.20 – 1.44 | 0.291 | 0.62 | 0.14 – 2.04 | 0.478 | 0.57 | 0.08 – 2.20 | 0.472 |
| **Years with provider** |  |  |  |  |  |  |  |  |  |
| < 4 | Reference | |  |  |  |  |  |  |  |
| 4 – 9 | 0.79 | 0.57 – 1.08 | 0.134 | 0.72 | 0.49 – 1.06 | 0.100 | 0.83 | 0.46 – 1.49 | 0.542 |
| 10 – 19 | 0.90 | 0.66 – 1.23 | 0.500 | 0.82 | 0.56 – 1.22 | 0.333 | 1.11 | 0.65 – 1.92 | 0.701 |
| ≥ 20 | 0.65 | 0.48 – 0.89 | **0.006** | 0.64 | 0.43 – 0.96 | **0.032** | 0.78 | 0.47 – 1.32 | 0.353 |
| **RIO category** |  |  |  |  |  |  |  |  |  |
| Large urban | Reference | |  |  |  |  |  |  |  |
| Urban | 0.99 | 0.75 – 1.31 | 0.968 | 0.98 | 0.70 – 1.38 | 0.911 | 0.92 | 0.55 – 1.50 | 0.730 |
| Small urban | 1.01 | 0.71 – 1.43 | 0.963 | 1.04 | 0.65 – 1.64 | 0.877 | 0.96 | 0.55 – 1.70 | 0.896 |
| Rural | 0.66 | 0.38 – 1.11 | 0.128 | 0.47 | 0.21 – 0.99 | 0.055 | 0.91 | 0.40 – 2.00 | 0.818 |
| **Dependency** |  |  |  |  |  |  |  |  |  |
| 1^st^ quintile *(least marginalized)* | Reference | |  |  |  |  |  |  |  |
| 2^nd^ quintile | 0.86 | 0.61 – 1.22 | 0.393 | 0.87 | 0.58 – 1.31 | 0.505 | 1.23 | 0.60 – 2.60 | 0.578 |
| 3^rd^ quintile | 0.90 | 0.63 – 1.29 | 0.573 | 0.82 | 0.53 – 1.27 | 0.378 | 1.33 | 0.65 – 2.82 | 0.440 |
| 4^th^ quintile | 0.86 | 0.59 – 1.26 | 0.451 | 0.87 | 0.54 – 1.38 | 0.550 | 1.19 | 0.57 – 2.56 | 0.643 |
| 5^th^ quintile *(most marginalized)* | 0.69 | 0.47 – 1.01 | 0.060 | 0.74 | 0.43 – 1.24 | 0.255 | 1.10 | 0.54 – 2.32 | 0.798 |
| **Material Deprivation** |  |  |  |  |  |  |  |  |  |
| 1^st^ quintile *(least marginalized)* | Reference | |  |  |  |  |  |  |  |
| 2^nd^ quintile | 1.04 | 0.75 – 1.43 | 0.820 | 0.89 | 0.59 – 1.34 | 0.583 | 1.31 | 0.75 – 2.29 | 0.348 |
| 3^rd^ quintile | 1.02 | 0.72 – 1.45 | 0.893 | 0.90 | 0.57 – 1.39 | 0.625 | 1.25 | 0.69 – 2.26 | 0.462 |
| 4^th^ quintile | 1.16 | 0.80 – 1.67 | 0.444 | 1.16 | 0.72 – 1.86 | 0.543 | 1.27 | 0.68 – 2.38 | 0.449 |
| 5^th^ quintile *(most marginalized)* | 0.82 | 0.53 – 1.25 | 0.353 | 0.85 | 0.50 – 1.44 | 0.549 | 0.69 | 0.31 – 1.46 | 0.339 |
| **Ethnic Concentration** |  |  |  |  |  |  |  |  |  |
| 1^st^ quintile *(least marginalized)* | Reference | |  |  |  |  |  |  |  |
| 2^nd^ quintile | 1.01 | 0.69 – 1.48 | 0.940 | 0.94 | 0.56 – 1.57 | 0.806 | 1.25 | 0.70 – 2.24 | 0.461 |
| 3^rd^ quintile | 0.87 | 0.57 – 1.31 | 0.490 | 0.80 | 0.46 – 1.40 | 0.435 | 1.04 | 0.55 – 1.97 | 0.912 |
| 4^th^ quintile | 1.05 | 0.68 – 1.63 | 0.831 | 0.86 | 0.49 – 1.53 | 0.609 | 1.27 | 0.62 – 2.65 | 0.516 |
| 5^th^ quintile *(most marginalized)* | 1.04 | 0.65 – 1.67 | 0.866 | 0.89 | 0.49 – 1.64 | 0.711 | 1.17 | 0.52 – 2.65 | 0.703 |
| **Residential Instability** |  |  |  |  |  |  |  |  |  |
| 1^st^ quintile *(least marginalized)* | Reference | |  |  |  |  |  |  |  |
| 2^nd^ quintile | 1.15 | 0.80 – 1.64 | 0.450 | 1.40 | 0.90 – 2.18 | 0.132 | 0.82 | 0.43 – 1.58 | 0.558 |
| 3^rd^ quintile | 1.10 | 0.76 – 1.60 | 0.603 | 1.27 | 0.79 – 2.04 | 0.325 | 0.97 | 0.52 – 1.82 | 0.917 |
| 4^th^ quintile | 1.23 | 0.84 – 1.80 | 0.287 | 1.31 | 0.80 – 2.13 | 0.285 | 1.15 | 0.61 – 2.18 | 0.663 |
| 5^th^ quintile *(most marginalized)* | 1.13 | 0.77 – 1.65 | 0.544 | 1.48 | 0.92 – 2.39 | 0.106 | 0.79 | 0.40 – 1.56 | 0.490 |

Associations between factors and primary care provider sometimes, rarely, or never spending enough time with patient, stratified by number of encounters over past 12 months.

| **Predictors** | **Full Model, N = 2,677** | |  | **≤ 3 encounters, N = 1,648** | |  | **> 3 encounters, N = 1,039** | |  |
| --- | --- | --- | --- | --- | --- | --- | --- | --- | --- |
|  | **Odds Ratios** | **CI (95%)** | **p** | **Odds Ratios** | **CI (95%)** | **p** | **Odds Ratios** | **CI (95%)** | **p** |
| **Telehealth access** | 0.89 | 0.71 – 1.13 | 0.335 | 0.81 | 0.61 – 1.09 | 0.156 | 1.06 | 0.72 – 1.58 | 0.769 |
| **Health record access** | 1.09 | 0.54 – 2.03 | 0.799 | 1.13 | 0.41 – 2.67 | 0.802 | 1.19 | 0.42 – 2.92 | 0.715 |
| **Online booking access** | 0.92 | 0.47 – 1.64 | 0.779 | 0.96 | 0.42 – 1.95 | 0.914 | 0.73 | 0.20 – 2.03 | 0.579 |
| **Sex** |  |  |  |  |  |  |  |  |  |
| Female | Reference | |  |  |  |  |  |  |  |
| Male | 0.84 | 0.66 – 1.06 | 0.136 | 0.90 | 0.67 – 1.21 | 0.490 | 0.76 | 0.51 – 1.12 | 0.175 |
| **Financial Situation** |  |  |  |  |  |  |  |  |  |
| Very comfortable | Reference | |  |  |  |  |  |  |  |
| Comfortable | 1.12 | 0.83 – 1.54 | 0.468 | 1.03 | 0.69 – 1.54 | 0.902 | 1.28 | 0.77 – 2.22 | 0.353 |
| Tight/Very tight/Poor | 1.53 | 1.06 – 2.22 | **0.025** | 1.50 | 0.93 – 2.43 | 0.098 | 1.60 | 0.88 – 3.00 | 0.131 |
| **Educational Attainment** |  |  |  |  |  |  |  |  |  |
| High school or less | Reference | |  |  |  |  |  |  |  |
| Some college/university | 0.77 | 0.48 – 1.21 | 0.276 | 0.70 | 0.37 – 1.27 | 0.255 | 0.89 | 0.41 – 1.80 | 0.749 |
| Completed college/university | 1.07 | 0.82 – 1.41 | 0.611 | 1.13 | 0.79 – 1.62 | 0.509 | 1.02 | 0.66 – 1.58 | 0.921 |
| Post-graduate/professional degree | 0.84 | 0.56 – 1.25 | 0.395 | 0.80 | 0.46 – 1.35 | 0.406 | 1.01 | 0.52 – 1.93 | 0.974 |
| **Primary language spoken** |  |  |  |  |  |  |  |  |  |
| English | Reference | |  |  |  |  |  |  |  |
| Other | 1.75 | 1.28 – 2.38 | **<0.001** | 1.55 | 1.01 – 2.34 | **0.042** | 1.95 | 1.18 – 3.17 | **0.008** |
| **Self-perceived health** |  |  |  |  |  |  |  |  |  |
| Poor | Reference | |  |  |  |  |  |  |  |
| Fair | 1.21 | 0.71 – 2.14 | 0.496 | 0.95 | 0.43 – 2.24 | 0.901 | 1.48 | 0.70 – 3.34 | 0.318 |
| Good | 1.05 | 0.63 – 1.82 | 0.851 | 0.69 | 0.33 – 1.58 | 0.356 | 1.56 | 0.76 – 3.46 | 0.242 |
| Very good | 0.88 | 0.52 – 1.54 | 0.642 | 0.63 | 0.30 – 1.42 | 0.236 | 1.01 | 0.46 – 2.36 | 0.974 |
| Excellent | 0.78 | 0.43 – 1.44 | 0.412 | 0.51 | 0.23 – 1.21 | 0.110 | 1.26 | 0.47 – 3.41 | 0.649 |
| **ADG Score** |  |  |  |  |  |  |  |  |  |
| < 3 | Reference | |  |  |  |  |  |  |  |
| 3 – 4 | 1.06 | 0.70 – 1.64 | 0.786 | 1.17 | 0.75 – 1.86 | 0.486 | 0.50 | 0.12 – 2.59 | 0.364 |
| 5 – 6 | 0.91 | 0.60 – 1.42 | 0.680 | 0.83 | 0.51 – 1.35 | 0.441 | 1.19 | 0.36 – 5.46 | 0.796 |
| 7 – 8 | 0.80 | 0.52 – 1.27 | 0.337 | 0.88 | 0.52 – 1.51 | 0.646 | 0.81 | 0.24 – 3.71 | 0.751 |
| ≥ 9 | 1.10 | 0.72 – 1.72 | 0.663 | 1.18 | 0.67 – 2.06 | 0.567 | 1.31 | 0.41 – 5.89 | 0.684 |
| **Program type** |  |  |  |  |  |  |  |  |  |
| Enhanced FFS | Reference | |  |  |  |  |  |  |  |
| Capitation | 0.75 | 0.59 – 0.98 | **0.031** | 0.83 | 0.58 – 1.19 | 0.301 | 0.58 | 0.39 – 0.87 | **0.008** |
| Other | 0.59 | 0.20 – 1.44 | 0.291 | 0.61 | 0.14 – 1.94 | 0.455 | 0.62 | 0.09 – 2.46 | 0.548 |
| **Years with provider** |  |  |  |  |  |  |  |  |  |
| < 4 | Reference | |  |  |  |  |  |  |  |
| 4 – 9 | 0.79 | 0.57 – 1.08 | 0.134 | 0.80 | 0.54 – 1.19 | 0.273 | 0.77 | 0.45 – 1.33 | 0.352 |
| 10 – 19 | 0.90 | 0.66 – 1.23 | 0.500 | 0.80 | 0.53 – 1.20 | 0.282 | 1.05 | 0.63 – 1.76 | 0.851 |
| ≥ 20 | 0.65 | 0.48 – 0.89 | **0.006** | 0.67 | 0.45 – 1.00 | **0.048** | 0.63 | 0.38 – 1.07 | 0.084 |
| **RIO category** |  |  |  |  |  |  |  |  |  |
| Large urban | Reference | |  |  |  |  |  |  |  |
| Urban | 0.99 | 0.75 – 1.31 | 0.968 | 1.03 | 0.72 – 1.46 | 0.878 | 0.97 | 0.61 – 1.52 | 0.898 |
| Small urban | 1.01 | 0.71 – 1.43 | 0.963 | 0.91 | 0.58 – 1.42 | 0.686 | 1.24 | 0.68 – 2.23 | 0.483 |
| Rural | 0.66 | 0.38 – 1.11 | 0.128 | 0.61 | 0.31 – 1.17 | 0.150 | 0.78 | 0.29 – 1.92 | 0.600 |
| **Dependency** |  |  |  |  |  |  |  |  |  |
| 1^st^ quintile *(least marginalized)* | Reference | |  |  |  |  |  |  |  |
| 2^nd^ quintile | 0.86 | 0.61 – 1.22 | 0.393 | 0.73 | 0.47 – 1.12 | 0.151 | 1.40 | 0.77 – 2.57 | 0.273 |
| 3^rd^ quintile | 0.90 | 0.63 – 1.29 | 0.573 | 0.57 | 0.36 – 0.90 | **0.016** | 2.20 | 1.20 – 4.07 | **0.011** |
| 4^th^ quintile | 0.86 | 0.59 – 1.26 | 0.451 | 0.69 | 0.43 – 1.11 | 0.125 | 1.40 | 0.73 – 2.70 | 0.313 |
| 5^th^ quintile *(most marginalized)* | 0.69 | 0.47 – 1.01 | 0.060 | 0.61 | 0.37 – 1.00 | 0.052 | 0.99 | 0.51 – 1.93 | 0.968 |
| **Material Deprivation** |  |  |  |  |  |  |  |  |  |
| 1^st^ quintile *(least marginalized)* | Reference | |  |  |  |  |  |  |  |
| 2^nd^ quintile | 1.04 | 0.75 – 1.43 | 0.820 | 1.12 | 0.74 – 1.68 | 0.603 | 0.93 | 0.54 – 1.60 | 0.794 |
| 3^rd^ quintile | 1.02 | 0.72 – 1.45 | 0.893 | 1.34 | 0.87 – 2.06 | 0.181 | 0.58 | 0.31 – 1.08 | 0.088 |
| 4^th^ quintile | 1.16 | 0.80 – 1.67 | 0.444 | 1.34 | 0.83 – 2.18 | 0.233 | 0.93 | 0.51 – 1.69 | 0.807 |
| 5^th^ quintile *(most marginalized)* | 0.82 | 0.53 – 1.25 | 0.353 | 0.83 | 0.46 – 1.46 | 0.519 | 0.78 | 0.40 – 1.50 | 0.456 |
| **Ethnic Concentration** |  |  |  |  |  |  |  |  |  |
| 1^st^ quintile *(least marginalized)* | Reference | |  |  |  |  |  |  |  |
| 2^nd^ quintile | 1.01 | 0.69 – 1.48 | 0.940 | 1.29 | 0.80 – 2.07 | 0.295 | 0.72 | 0.37 – 1.39 | 0.330 |
| 3^rd^ quintile | 0.87 | 0.57 – 1.31 | 0.490 | 1.03 | 0.61 – 1.75 | 0.908 | 0.59 | 0.29 – 1.17 | 0.129 |
| 4^th^ quintile | 1.05 | 0.68 – 1.63 | 0.831 | 1.18 | 0.68 – 2.08 | 0.554 | 0.91 | 0.44 – 1.88 | 0.788 |
| 5^th^ quintile *(most marginalized)* | 1.04 | 0.65 – 1.67 | 0.866 | 1.22 | 0.67 – 2.24 | 0.512 | 0.87 | 0.40 – 1.92 | 0.729 |
| **Residential Instability** |  |  |  |  |  |  |  |  |  |
| 1^st^ quintile *(least marginalized)* | Reference | |  |  |  |  |  |  |  |
| 2^nd^ quintile | 1.15 | 0.80 – 1.64 | 0.450 | 1.26 | 0.80 – 1.99 | 0.319 | 0.98 | 0.53 – 1.80 | 0.949 |
| 3^rd^ quintile | 1.10 | 0.76 – 1.60 | 0.603 | 1.24 | 0.78 – 1.99 | 0.366 | 0.88 | 0.46 – 1.64 | 0.683 |
| 4^th^ quintile | 1.23 | 0.84 – 1.80 | 0.287 | 1.43 | 0.87 – 2.35 | 0.158 | 1.09 | 0.58 – 2.02 | 0.791 |
| 5^th^ quintile *(most marginalized)* | 1.13 | 0.77 – 1.65 | 0.544 | 1.13 | 0.68 – 1.88 | 0.625 | 1.10 | 0.60 – 2.03 | 0.758 |

Associations between factors and primary care provider sometimes, rarely, or never involving patient in decision-making in treatment, stratified by patient age.

| **Predictors** | **Full Model, N = 2,677** | |  | **Age ≤ 60 years, N = 1,378** | |  | **Age > 60 years, N = 1,308** | |  |
| --- | --- | --- | --- | --- | --- | --- | --- | --- | --- |
|  | **Odds Ratios** | **CI (95%)** | **p** | **Odds Ratios** | **CI (95%)** | **p** | **Odds Ratios** | **CI (95%)** | **p** |
| **Telehealth access** | 0.87 | 0.67 – 1.12 | 0.275 | 0.94 | 0.67 – 1.32 | 0.718 | 0.66 | 0.43 – 1.00 | 0.050 |
| **Health record access** | 1.14 | 0.53 – 2.21 | 0.724 | 1.07 | 0.41 – 2.45 | 0.878 | 1.45 | 0.32 – 4.79 | 0.580 |
| **Online booking access** | 0.93 | 0.44 – 1.77 | 0.831 | 0.91 | 0.40 – 1.86 | 0.806 | 0.48 | 0.03 – 2.60 | 0.498 |
| **Sex** |  |  |  |  |  |  |  |  |  |
| Female | Reference | |  |  |  |  |  |  |  |
| Male | 0.91 | 0.71 – 1.18 | 0.491 | 1.00 | 0.71 – 1.39 | 0.986 | 0.99 | 0.64 – 1.52 | 0.968 |
| **Financial Situation** |  |  |  |  |  |  |  |  |  |
| Very comfortable | Reference | |  |  |  |  |  |  |  |
| Comfortable | 1.15 | 0.81 – 1.65 | 0.452 | 0.69 | 0.44 – 1.09 | 0.102 | 1.83 | 0.99 – 3.68 | 0.069 |
| Tight/Very tight/Poor | 1.55 | 1.03 – 2.36 | **0.037** | 0.94 | 0.56 – 1.59 | 0.810 | 2.52 | 1.21 – 5.53 | **0.016** |
| **Educational Attainment** |  |  |  |  |  |  |  |  |  |
| High school or less | Reference | |  |  |  |  |  |  |  |
| Some college/university | 0.65 | 0.37 – 1.07 | 0.105 | 0.86 | 0.40 – 1.78 | 0.700 | 0.52 | 0.22 – 1.11 | 0.113 |
| Completed college/university | 0.95 | 0.71 – 1.28 | 0.731 | 1.21 | 0.77 – 1.94 | 0.414 | 0.64 | 0.40 – 1.01 | 0.058 |
| Post-graduate/professional degree | 0.84 | 0.54 – 1.29 | 0.433 | 1.06 | 0.58 – 1.92 | 0.849 | 0.53 | 0.23 – 1.14 | 0.123 |
| **Primary language spoken** |  |  |  |  |  |  |  |  |  |
| English | Reference | |  |  |  |  |  |  |  |
| Other | 1.62 | 1.14 – 2.28 | **0.006** | 1.42 | 0.93 – 2.15 | 0.099 | 1.53 | 0.77 – 2.88 | 0.207 |
| **Self-perceived health** |  |  |  |  |  |  |  |  |  |
| Poor | Reference | |  |  |  |  |  |  |  |
| Fair | 0.94 | 0.53 – 1.73 | 0.841 | 0.64 | 0.27 – 1.61 | 0.330 | 1.31 | 0.59 – 3.15 | 0.525 |
| Good | 0.97 | 0.57 – 1.73 | 0.915 | 0.66 | 0.31 – 1.54 | 0.315 | 1.19 | 0.55 – 2.82 | 0.674 |
| Very good | 0.68 | 0.39 – 1.24 | 0.196 | 0.52 | 0.24 – 1.22 | 0.112 | 0.64 | 0.27 – 1.63 | 0.329 |
| Excellent | 0.56 | 0.29 – 1.10 | 0.085 | 0.32 | 0.13 – 0.81 | **0.013** | 0.88 | 0.28 – 2.69 | 0.822 |
| **ADG Score** |  |  |  |  |  |  |  |  |  |
| < 3 | Reference | |  |  |  |  |  |  |  |
| 3 – 4 | 1.08 | 0.68 – 1.76 | 0.744 | 1.43 | 0.84 – 2.50 | 0.201 | 0.69 | 0.26 – 2.04 | 0.468 |
| 5 – 6 | 0.96 | 0.60 – 1.56 | 0.865 | 1.09 | 0.62 – 1.95 | 0.769 | 0.94 | 0.38 – 2.71 | 0.904 |
| 7 – 8 | 0.72 | 0.44 – 1.21 | 0.202 | 0.86 | 0.46 – 1.62 | 0.632 | 0.73 | 0.28 – 2.16 | 0.545 |
| ≥ 9 | 1.03 | 0.64 – 1.70 | 0.893 | 1.04 | 0.57 – 1.95 | 0.895 | 1.18 | 0.48 – 3.34 | 0.739 |
| **Program type** |  |  |  |  |  |  |  |  |  |
| Enhanced FFS | Reference | |  |  |  |  |  |  |  |
| Capitation | 0.83 | 0.63 – 1.11 | 0.207 | 0.79 | 0.55 – 1.15 | 0.214 | 0.92 | 0.56 – 1.54 | 0.745 |
| Other | 0.69 | 0.20 – 1.84 | 0.506 | 0.57 | 0.09 – 2.24 | 0.479 | 0.66 | 0.10 – 2.66 | 0.608 |
| **Years with provider** |  |  |  |  |  |  |  |  |  |
| < 4 | Reference | |  |  |  |  |  |  |  |
| 4 – 9 | 0.93 | 0.64 – 1.34 | 0.679 | 1.06 | 0.68 – 1.68 | 0.791 | 0.66 | 0.33 – 1.31 | 0.243 |
| 10 – 19 | 1.23 | 0.86 – 1.75 | 0.257 | 1.24 | 0.78 – 1.96 | 0.363 | 1.21 | 0.67 – 2.23 | 0.524 |
| ≥ 20 | 0.95 | 0.67 – 1.35 | 0.773 | 1.04 | 0.65 – 1.67 | 0.867 | 1.01 | 0.58 – 1.80 | 0.962 |
| **RIO category** |  |  |  |  |  |  |  |  |  |
| Large urban | Reference | |  |  |  |  |  |  |  |
| Urban | 0.90 | 0.66 – 1.22 | 0.498 | 0.71 | 0.48 – 1.04 | 0.084 | 1.10 | 0.64 – 1.89 | 0.721 |
| Small urban | 1.02 | 0.69 – 1.51 | 0.920 | 0.98 | 0.58 – 1.63 | 0.932 | 1.19 | 0.62 – 2.29 | 0.603 |
| Rural | 0.69 | 0.37 – 1.24 | 0.230 | 0.43 | 0.16 – 1.01 | 0.067 | 1.18 | 0.47 – 2.86 | 0.716 |
| **Dependency** |  |  |  |  |  |  |  |  |  |
| 1^st^ quintile *(least marginalized)* | Reference | |  |  |  |  |  |  |  |
| 2^nd^ quintile | 1.22 | 0.83 – 1.80 | 0.306 | 1.22 | 0.77 – 1.91 | 0.392 | 1.54 | 0.69 – 3.56 | 0.298 |
| 3^rd^ quintile | 1.29 | 0.87 – 1.92 | 0.200 | 1.17 | 0.73 – 1.89 | 0.510 | 1.94 | 0.88 – 4.51 | 0.108 |
| 4^th^ quintile | 0.96 | 0.62 – 1.48 | 0.863 | 0.70 | 0.39 – 1.21 | 0.204 | 1.79 | 0.81 – 4.18 | 0.163 |
| 5^th^ quintile *(most marginalized)* | 0.68 | 0.43 – 1.08 | 0.101 | 0.50 | 0.25 – 0.95 | **0.040** | 1.32 | 0.60 – 3.08 | 0.499 |
| **Material Deprivation** |  |  |  |  |  |  |  |  |  |
| 1^st^ quintile *(least marginalized)* | Reference | |  |  |  |  |  |  |  |
| 2^nd^ quintile | 1.02 | 0.71 – 1.46 | 0.934 | 0.92 | 0.58 – 1.47 | 0.729 | 1.15 | 0.62 – 2.15 | 0.658 |
| 3^rd^ quintile | 1.01 | 0.69 – 1.49 | 0.946 | 0.91 | 0.54 – 1.51 | 0.716 | 1.16 | 0.61 – 2.20 | 0.658 |
| 4^th^ quintile | 1.09 | 0.72 – 1.65 | 0.698 | 1.61 | 0.94 – 2.74 | 0.080 | 0.71 | 0.34 – 1.45 | 0.344 |
| 5^th^ quintile *(most marginalized)* | 0.76 | 0.47 – 1.22 | 0.264 | 0.95 | 0.52 – 1.75 | 0.880 | 0.57 | 0.25 – 1.27 | 0.173 |
| **Ethnic Concentration** |  |  |  |  |  |  |  |  |  |
| 1^st^ quintile *(least marginalized)* | Reference | |  |  |  |  |  |  |  |
| 2^nd^ quintile | 1.17 | 0.77 – 1.79 | 0.460 | 1.55 | 0.86 – 2.82 | 0.150 | 1.03 | 0.54 – 1.96 | 0.930 |
| 3^rd^ quintile | 0.86 | 0.54 – 1.38 | 0.535 | 1.01 | 0.53 – 1.95 | 0.971 | 0.75 | 0.35 – 1.57 | 0.449 |
| 4^th^ quintile | 1.15 | 0.70 – 1.90 | 0.573 | 0.88 | 0.45 – 1.74 | 0.705 | 1.81 | 0.84 – 3.98 | 0.133 |
| 5^th^ quintile *(most marginalized)* | 1.37 | 0.81 – 2.32 | 0.241 | 1.28 | 0.64 – 2.59 | 0.486 | 1.62 | 0.67 – 3.94 | 0.282 |
| **Residential Instability** |  |  |  |  |  |  |  |  |  |
| 1^st^ quintile *(least marginalized)* | Reference | |  |  |  |  |  |  |  |
| 2^nd^ quintile | 1.20 | 0.80 – 1.79 | 0.377 | 1.39 | 0.85 – 2.28 | 0.187 | 1.20 | 0.56 – 2.59 | 0.639 |
| 3^rd^ quintile | 1.23 | 0.81 – 1.86 | 0.326 | 1.32 | 0.78 – 2.23 | 0.289 | 1.30 | 0.62 – 2.78 | 0.485 |
| 4^th^ quintile | 1.26 | 0.82 – 1.94 | 0.293 | 1.00 | 0.57 – 1.74 | 0.989 | 1.89 | 0.92 – 4.01 | 0.087 |
| 5^th^ quintile *(most marginalized)* | 1.28 | 0.84 – 1.95 | 0.258 | 1.16 | 0.68 – 1.97 | 0.586 | 1.73 | 0.82 – 3.78 | 0.160 |

Associations between factors and primary care provider sometimes, rarely, or never involving patient in decision-making in treatment, stratified by number of encounters over past 12 months.

| **Predictors** | **Full Model, N = 2,677** | |  | **≤ 3 encounters, N = 1,648** | |  | **> 3 encounters, N = 1,039** | |  |
| --- | --- | --- | --- | --- | --- | --- | --- | --- | --- |
|  | **Odds Ratios** | **CI (95%)** | **p** | **Odds Ratios** | **CI (95%)** | **p** | **Odds Ratios** | **CI (95%)** | **p** |
| **Telehealth access** | 0.87 | 0.67 – 1.12 | 0.275 | 0.91 | 0.66 – 1.27 | 0.570 | 0.78 | 0.50 – 1.21 | 0.266 |
| **Health record access** | 1.14 | 0.53 – 2.21 | 0.724 | 1.61 | 0.61 – 3.76 | 0.297 | 0.73 | 0.17 – 2.21 | 0.622 |
| **Online booking access** | 0.93 | 0.44 – 1.77 | 0.831 | 1.34 | 0.58 – 2.79 | 0.453 | 0.28 | 0.02 – 1.38 | 0.216 |
| **Sex** |  |  |  |  |  |  |  |  |  |
| Female | Reference | |  |  |  |  |  |  |  |
| Male | 0.91 | 0.71 – 1.18 | 0.491 | 0.91 | 0.65 – 1.26 | 0.556 | 0.97 | 0.62 – 1.49 | 0.884 |
| **Financial Situation** |  |  |  |  |  |  |  |  |  |
| Very comfortable | Reference | |  |  |  |  |  |  |  |
| Comfortable | 1.15 | 0.81 – 1.65 | 0.452 | 1.14 | 0.73 – 1.84 | 0.575 | 1.20 | 0.69 – 2.21 | 0.531 |
| Tight/Very tight/Poor | 1.55 | 1.03 – 2.36 | **0.037** | 1.89 | 1.12 – 3.27 | **0.020** | 1.15 | 0.57 – 2.33 | 0.699 |
| **Educational Attainment** |  |  |  |  |  |  |  |  |  |
| High school or less | Reference | |  |  |  |  |  |  |  |
| Some college/university | 0.65 | 0.37 – 1.07 | 0.105 | 0.49 | 0.23 – 0.97 | 0.050 | 0.82 | 0.34 – 1.81 | 0.641 |
| Completed college/university | 0.95 | 0.71 – 1.28 | 0.731 | 0.99 | 0.68 – 1.47 | 0.979 | 0.91 | 0.56 – 1.50 | 0.705 |
| Post-graduate/professional degree | 0.84 | 0.54 – 1.29 | 0.433 | 0.62 | 0.33 – 1.13 | 0.130 | 1.32 | 0.66 – 2.59 | 0.432 |
| **Primary language spoken** |  |  |  |  |  |  |  |  |  |
| English | Reference | |  |  |  |  |  |  |  |
| Other | 1.62 | 1.14 – 2.28 | **0.006** | 1.43 | 0.87 – 2.28 | 0.147 | 2.16 | 1.24 – 3.70 | **0.006** |
| **Self-perceived health** |  |  |  |  |  |  |  |  |  |
| Poor | Reference | |  |  |  |  |  |  |  |
| Fair | 0.94 | 0.53 – 1.73 | 0.841 | 1.04 | 0.44 – 2.70 | 0.927 | 0.74 | 0.33 – 1.73 | 0.471 |
| Good | 0.97 | 0.57 – 1.73 | 0.915 | 0.78 | 0.35 – 1.94 | 0.567 | 1.07 | 0.51 – 2.41 | 0.865 |
| Very good | 0.68 | 0.39 – 1.24 | 0.196 | 0.62 | 0.28 – 1.54 | 0.271 | 0.54 | 0.23 – 1.33 | 0.170 |
| Excellent | 0.56 | 0.29 – 1.10 | 0.085 | 0.40 | 0.16 – 1.06 | 0.054 | 0.88 | 0.30 – 2.50 | 0.810 |
| **ADG Score** |  |  |  |  |  |  |  |  |  |
| < 3 | Reference | |  |  |  |  |  |  |  |
| 3 – 4 | 1.08 | 0.68 – 1.76 | 0.744 | 1.11 | 0.67 – 1.90 | 0.686 | 0.61 | 0.17 – 2.52 | 0.460 |
| 5 – 6 | 0.96 | 0.60 – 1.56 | 0.865 | 1.03 | 0.61 – 1.79 | 0.909 | 0.55 | 0.17 – 2.13 | 0.337 |
| 7 – 8 | 0.72 | 0.44 – 1.21 | 0.202 | 0.94 | 0.51 – 1.74 | 0.850 | 0.36 | 0.11 – 1.40 | 0.105 |
| ≥ 9 | 1.03 | 0.64 – 1.70 | 0.893 | 1.31 | 0.70 – 2.47 | 0.398 | 0.64 | 0.21 – 2.43 | 0.470 |
| **Program type** |  |  |  |  |  |  |  |  |  |
| Enhanced FFS | Reference | |  |  |  |  |  |  |  |
| Capitation | 0.83 | 0.63 – 1.11 | 0.207 | 0.77 | 0.52 – 1.15 | 0.188 | 0.85 | 0.54 – 1.34 | 0.486 |
| Other | 0.69 | 0.20 – 1.84 | 0.506 | 0.46 | 0.07 – 1.79 | 0.330 | 1.28 | 0.19 – 5.30 | 0.759 |
| **Years with provider** |  |  |  |  |  |  |  |  |  |
| < 4 | Reference | |  |  |  |  |  |  |  |
| 4 – 9 | 0.93 | 0.64 – 1.34 | 0.679 | 0.97 | 0.62 – 1.54 | 0.912 | 0.73 | 0.37 – 1.44 | 0.359 |
| 10 – 19 | 1.23 | 0.86 – 1.75 | 0.257 | 1.02 | 0.64 – 1.62 | 0.935 | 1.49 | 0.82 – 2.76 | 0.194 |
| ≥ 20 | 0.95 | 0.67 – 1.35 | 0.773 | 0.90 | 0.58 – 1.40 | 0.635 | 1.00 | 0.55 – 1.86 | 0.998 |
| **RIO category** |  |  |  |  |  |  |  |  |  |
| Large urban | Reference | |  |  |  |  |  |  |  |
| Urban | 0.90 | 0.66 – 1.22 | 0.498 | 0.82 | 0.55 – 1.23 | 0.344 | 1.02 | 0.61 – 1.68 | 0.949 |
| Small urban | 1.02 | 0.69 – 1.51 | 0.920 | 0.97 | 0.60 – 1.57 | 0.900 | 1.04 | 0.51 – 2.08 | 0.907 |
| Rural | 0.69 | 0.37 – 1.24 | 0.230 | 0.76 | 0.36 – 1.52 | 0.447 | 0.49 | 0.13 – 1.55 | 0.258 |
| **Dependency** |  |  |  |  |  |  |  |  |  |
| 1^st^ quintile *(least marginalized)* | Reference | |  |  |  |  |  |  |  |
| 2^nd^ quintile | 1.22 | 0.83 – 1.80 | 0.306 | 1.17 | 0.73 – 1.87 | 0.512 | 1.40 | 0.69 – 2.86 | 0.347 |
| 3^rd^ quintile | 1.29 | 0.87 – 1.92 | 0.200 | 0.90 | 0.54 – 1.48 | 0.668 | 2.56 | 1.28 – 5.23 | **0.009** |
| 4^th^ quintile | 0.96 | 0.62 – 1.48 | 0.863 | 0.73 | 0.42 – 1.25 | 0.256 | 1.51 | 0.70 – 3.25 | 0.288 |
| 5^th^ quintile *(most marginalized)* | 0.68 | 0.43 – 1.08 | 0.101 | 0.45 | 0.24 – 0.82 | **0.010** | 1.25 | 0.59 – 2.66 | 0.558 |
| **Material Deprivation** |  |  |  |  |  |  |  |  |  |
| 1^st^ quintile *(least marginalized)* | Reference | |  |  |  |  |  |  |  |
| 2^nd^ quintile | 1.02 | 0.71 – 1.46 | 0.934 | 0.88 | 0.56 – 1.40 | 0.600 | 1.12 | 0.59 – 2.13 | 0.722 |
| 3^rd^ quintile | 1.01 | 0.69 – 1.49 | 0.946 | 1.20 | 0.75 – 1.93 | 0.443 | 0.61 | 0.29 – 1.25 | 0.178 |
| 4^th^ quintile | 1.09 | 0.72 – 1.65 | 0.698 | 1.24 | 0.73 – 2.10 | 0.426 | 0.77 | 0.37 – 1.57 | 0.468 |
| 5^th^ quintile *(most marginalized)* | 0.76 | 0.47 – 1.22 | 0.264 | 0.56 | 0.28 – 1.08 | 0.092 | 0.87 | 0.41 – 1.82 | 0.704 |
| **Ethnic Concentration** |  |  |  |  |  |  |  |  |  |
| 1^st^ quintile *(least marginalized)* | Reference | |  |  |  |  |  |  |  |
| 2^nd^ quintile | 1.17 | 0.77 – 1.79 | 0.460 | 1.45 | 0.87 – 2.45 | 0.158 | 0.70 | 0.32 – 1.51 | 0.360 |
| 3^rd^ quintile | 0.86 | 0.54 – 1.38 | 0.535 | 0.95 | 0.52 – 1.74 | 0.878 | 0.64 | 0.28 – 1.44 | 0.282 |
| 4^th^ quintile | 1.15 | 0.70 – 1.90 | 0.573 | 1.16 | 0.62 – 2.19 | 0.649 | 0.99 | 0.44 – 2.30 | 0.986 |
| 5^th^ quintile *(most marginalized)* | 1.37 | 0.81 – 2.32 | 0.241 | 1.40 | 0.72 – 2.76 | 0.323 | 1.15 | 0.48 – 2.83 | 0.749 |
| **Residential Instability** |  |  |  |  |  |  |  |  |  |
| 1^st^ quintile *(least marginalized)* | Reference | |  |  |  |  |  |  |  |
| 2^nd^ quintile | 1.20 | 0.80 – 1.79 | 0.377 | 1.20 | 0.73 – 1.95 | 0.471 | 1.26 | 0.59 – 2.69 | 0.552 |
| 3^rd^ quintile | 1.23 | 0.81 – 1.86 | 0.326 | 1.16 | 0.70 – 1.93 | 0.562 | 1.53 | 0.72 – 3.26 | 0.264 |
| 4^th^ quintile | 1.26 | 0.82 – 1.94 | 0.293 | 1.33 | 0.77 – 2.29 | 0.303 | 1.48 | 0.70 – 3.17 | 0.304 |
| 5^th^ quintile *(most marginalized)* | 1.28 | 0.84 – 1.95 | 0.258 | 0.84 | 0.47 – 1.47 | 0.539 | 2.31 | 1.15 – 4.76 | **0.021** |

Associations between factors and primary care provider sometimes, rarely, or never explaining things in a way that is easy to understand, stratified by patient age.

| **Predictors** | **Full Model, N = 2,677** | |  | **Age ≤ 60 years, N = 1,378** | |  | **Age > 60 years, N = 1,308** | |  |
| --- | --- | --- | --- | --- | --- | --- | --- | --- | --- |
|  | **Odds Ratios** | **CI (95%)** | **p** | **Odds Ratios** | **CI (95%)** | **p** | **Odds Ratios** | **CI (95%)** | **p** |
| **Telehealth access** | 1.13 | 0.80 – 1.60 | 0.505 | 1.04 | 0.66 – 1.65 | 0.867 | 1.30 | 0.73 – 2.37 | 0.387 |
| **Health record access** | 0.61 | 0.15 – 1.73 | 0.422 | 0.22 | 0.01 – 1.11 | 0.150 | 1.77 | 0.26 – 7.23 | 0.479 |
| **Online booking access** | 0.83 | 0.24 – 2.10 | 0.721 | 0.69 | 0.16 – 2.08 | 0.564 | 1.00 | 0.05 – 5.65 | 0.997 |
| **Sex** |  |  |  |  |  |  |  |  |  |
| Female | Reference | |  |  |  |  |  |  |  |
| Male | 0.84 | 0.59 – 1.18 | 0.326 | 0.79 | 0.49 – 1.25 | 0.330 | 1.17 | 0.66 – 2.05 | 0.583 |
| **Financial Situation** |  |  |  |  |  |  |  |  |  |
| Very comfortable | Reference | |  |  |  |  |  |  |  |
| Comfortable | 1.09 | 0.69 – 1.79 | 0.714 | 1.01 | 0.55 – 1.98 | 0.976 | 0.97 | 0.47 – 2.18 | 0.945 |
| Tight/Very tight/Poor | 1.35 | 0.78 – 2.38 | 0.289 | 1.18 | 0.57 – 2.52 | 0.665 | 1.38 | 0.55 – 3.53 | 0.494 |
| **Educational Attainment** |  |  |  |  |  |  |  |  |  |
| High school or less | Reference | |  |  |  |  |  |  |  |
| Some college/university | 0.72 | 0.34 – 1.39 | 0.347 | 0.62 | 0.19 – 1.71 | 0.383 | 0.81 | 0.28 – 1.97 | 0.659 |
| Completed college/university | 1.00 | 0.68 – 1.50 | 0.999 | 1.10 | 0.62 – 2.05 | 0.760 | 0.66 | 0.35 – 1.23 | 0.192 |
| Post-graduate/professional degree | 0.91 | 0.50 – 1.61 | 0.755 | 0.91 | 0.41 – 2.03 | 0.825 | 0.63 | 0.21 – 1.61 | 0.363 |
| **Primary language spoken** |  |  |  |  |  |  |  |  |  |
| English | Reference | |  |  |  |  |  |  |  |
| Other | 1.88 | 1.21 – 2.88 | **0.004** | 1.87 | 1.10 – 3.13 | **0.019** | 1.28 | 0.47 – 3.05 | 0.604 |
| **Self-perceived health** |  |  |  |  |  |  |  |  |  |
| Poor | Reference | |  |  |  |  |  |  |  |
| Fair | 0.89 | 0.45 – 1.85 | 0.743 | 0.74 | 0.27 – 2.19 | 0.560 | 1.07 | 0.40 – 3.21 | 0.902 |
| Good | 0.63 | 0.33 – 1.29 | 0.184 | 0.42 | 0.17 – 1.19 | 0.083 | 0.88 | 0.34 – 2.60 | 0.802 |
| Very good | 0.46 | 0.23 – 0.95 | **0.029** | 0.33 | 0.13 – 0.95 | **0.028** | 0.42 | 0.14 – 1.38 | 0.134 |
| Excellent | 0.47 | 0.20 – 1.08 | 0.069 | 0.35 | 0.12 – 1.08 | 0.057 | 0.30 | 0.04 – 1.52 | 0.175 |
| **ADG Score** |  |  |  |  |  |  |  |  |  |
| < 3 | Reference | |  |  |  |  |  |  |  |
| 3 – 4 | 0.65 | 0.35 – 1.24 | 0.181 | 0.93 | 0.44 – 2.06 | 0.845 | 0.28 | 0.09 – 0.97 | **0.038** |
| 5 – 6 | 0.62 | 0.33 – 1.17 | 0.129 | 0.86 | 0.40 – 1.94 | 0.708 | 0.34 | 0.11 – 1.09 | 0.057 |
| 7 – 8 | 0.61 | 0.32 – 1.17 | 0.124 | 0.84 | 0.37 – 1.96 | 0.675 | 0.33 | 0.11 – 1.08 | 0.053 |
| ≥ 9 | 0.82 | 0.45 – 1.55 | 0.520 | 1.03 | 0.46 – 2.39 | 0.943 | 0.50 | 0.18 – 1.53 | 0.191 |
| **Program type** |  |  |  |  |  |  |  |  |  |
| Enhanced FFS | Reference | |  |  |  |  |  |  |  |
| Capitation | 0.78 | 0.53 – 1.14 | 0.190 | 0.62 | 0.38 – 1.01 | 0.055 | 1.57 | 0.77 – 3.50 | 0.236 |
| Other | 0.59 | 0.09 – 2.09 | 0.481 | 0.48 | 0.02 – 2.81 | 0.504 | 0.82 | 0.04 – 5.50 | 0.859 |
| **Years with provider** |  |  |  |  |  |  |  |  |  |
| < 4 | Reference | |  |  |  |  |  |  |  |
| 4 – 9 | 0.93 | 0.56 – 1.54 | 0.766 | 0.82 | 0.44 – 1.56 | 0.546 | 1.21 | 0.48 – 3.07 | 0.689 |
| 10 – 19 | 1.22 | 0.75 – 1.98 | 0.426 | 1.03 | 0.56 – 1.93 | 0.918 | 1.79 | 0.79 – 4.26 | 0.171 |
| ≥ 20 | 1.07 | 0.67 – 1.72 | 0.793 | 1.14 | 0.61 – 2.14 | 0.683 | 1.39 | 0.63 – 3.24 | 0.423 |
| **RIO category** |  |  |  |  |  |  |  |  |  |
| Large urban | Reference | |  |  |  |  |  |  |  |
| Urban | 1.06 | 0.70 – 1.59 | 0.792 | 0.91 | 0.53 – 1.53 | 0.715 | 1.12 | 0.54 – 2.29 | 0.761 |
| Small urban | 0.99 | 0.56 – 1.72 | 0.970 | 1.21 | 0.58 – 2.50 | 0.602 | 0.63 | 0.25 – 1.54 | 0.315 |
| Rural | 1.03 | 0.48 – 2.15 | 0.940 | 0.91 | 0.53 – 1.53 | 0.409 | 1.35 | 0.47 – 3.80 | 0.568 |
| **Dependency** |  |  |  |  |  |  |  |  |  |
| 1^st^ quintile *(least marginalized)* | Reference | |  |  |  |  |  |  |  |
| 2^nd^ quintile | 1.23 | 0.70 – 2.14 | 0.467 | 1.23 | 0.66 – 2.28 | 0.517 | 2.90 | 0.63 – 20.49 | 0.206 |
| 3^rd^ quintile | 1.32 | 0.74 – 2.35 | 0.342 | 1.11 | 0.55 – 2.17 | 0.769 | 4.90 | 1.18 – 33.49 | 0.050 |
| 4^th^ quintile | 1.74 | 0.98 – 3.11 | 0.057 | 1.24 | 0.60 – 2.53 | 0.558 | 8.21 | 2.10 – 54.93 | **0.008** |
| 5^th^ quintile *(most marginalized)* | 1.54 | 0.86 – 2.79 | 0.150 | 0.94 | 0.40 – 2.12 | 0.881 | 8.10 | 2.12 – 53.77 | **0.008** |
| **Material Deprivation** |  |  |  |  |  |  |  |  |  |
| 1^st^ quintile *(least marginalized)* | Reference | |  |  |  |  |  |  |  |
| 2^nd^ quintile | 1.07 | 0.65 – 1.79 | 0.785 | 1.20 | 0.62 – 2.34 | 0.588 | 0.80 | 0.33 – 1.89 | 0.606 |
| 3^rd^ quintile | 0.98 | 0.56 – 1.70 | 0.942 | 1.06 | 0.51 – 2.18 | 0.881 | 0.76 | 0.31 – 1.85 | 0.548 |
| 4^th^ quintile | 1.48 | 0.86 – 2.57 | 0.155 | 1.87 | 0.91 – 3.90 | 0.089 | 1.12 | 0.47 – 2.68 | 0.804 |
| 5^th^ quintile *(most marginalized)* | 0.86 | 0.45 – 1.64 | 0.652 | 1.06 | 0.44 – 2.48 | 0.899 | 0.68 | 0.23 – 1.91 | 0.476 |
| **Ethnic Concentration** |  |  |  |  |  |  |  |  |  |
| 1^st^ quintile *(least marginalized)* | Reference | |  |  |  |  |  |  |  |
| 2^nd^ quintile | 0.82 | 0.45 – 1.46 | 0.495 | 0.83 | 0.34 – 1.96 | 0.675 | 0.91 | 0.38 – 2.10 | 0.825 |
| 3^rd^ quintile | 0.79 | 0.41 – 1.49 | 0.464 | 0.97 | 0.40 – 2.36 | 0.946 | 0.56 | 0.19 – 1.54 | 0.274 |
| 4^th^ quintile | 1.04 | 0.53 – 2.03 | 0.918 | 0.78 | 0.31 – 2.00 | 0.599 | 1.69 | 0.62 – 4.67 | 0.303 |
| 5^th^ quintile *(most marginalized)* | 1.73 | 0.88 – 3.46 | 0.115 | 1.42 | 0.57 – 3.71 | 0.462 | 2.06 | 0.69 – 6.19 | 0.194 |
| **Residential Instability** |  |  |  |  |  |  |  |  |  |
| 1^st^ quintile *(least marginalized)* | Reference | |  |  |  |  |  |  |  |
| 2^nd^ quintile | 0.76 | 0.44 – 1.30 | 0.322 | 0.86 | 0.44 – 1.66 | 0.659 | 0.73 | 0.26 – 2.04 | 0.547 |
| 3^rd^ quintile | 0.85 | 0.50 – 1.46 | 0.567 | 1.10 | 0.56 – 2.15 | 0.778 | 0.74 | 0.28 – 2.03 | 0.553 |
| 4^th^ quintile | 0.72 | 0.40 – 1.27 | 0.263 | 0.45 | 0.19 – 1.01 | 0.061 | 1.40 | 0.56 – 3.70 | 0.483 |
| 5^th^ quintile *(most marginalized)* | 0.82 | 0.47 – 1.41 | 0.464 | 1.07 | 0.54 – 2.13 | 0.845 | 0.71 | 0.26 – 1.98 | 0.499 |

Associations between factors and primary care provider sometimes, rarely, or never explaining things in a way that is easy to understand, stratified by number of encounters over past 12 months.

| **Predictors** | **Full Model, N = 2,677** | |  | **≤ 3 encounters, N = 1,648** | |  | **> 3 encounters, N = 1,039** | |  |
| --- | --- | --- | --- | --- | --- | --- | --- | --- | --- |
|  | **Odds Ratios** | **CI (95%)** | **p** | **Odds Ratios** | **CI (95%)** | **p** | **Odds Ratios** | **CI (95%)** | **p** |
| **Telehealth access** | 1.13 | 0.80 – 1.60 | 0.505 | 1.29 | 0.82 – 2.07 | 0.285 | 1.08 | 0.62 – 1.91 | 0.799 |
| **Health record access** | 0.61 | 0.15 – 1.73 | 0.422 | 0.87 | 0.13 – 3.16 | 0.854 | 0.39 | 0.02 – 1.97 | 0.364 |
| **Online booking access** | 0.83 | 0.24 – 2.10 | 0.721 | 1.00 | 0.23 – 2.98 | 0.994 | 0.42 | 0.02 – 2.41 | 0.433 |
| **Sex** |  |  |  |  |  |  |  |  |  |
| Female | Reference | |  |  |  |  |  |  |  |
| Male | 0.84 | 0.59 – 1.18 | 0.326 | 0.99 | 0.63 – 1.55 | 0.976 | 0.66 | 0.36 – 1.16 | 0.158 |
| **Financial Situation** |  |  |  |  |  |  |  |  |  |
| Very comfortable | Reference | |  |  |  |  |  |  |  |
| Comfortable | 1.09 | 0.69 – 1.79 | 0.714 | 1.19 | 0.65 – 2.29 | 0.593 | 1.04 | 0.50 – 2.36 | 0.919 |
| Tight/Very tight/Poor | 1.35 | 0.78 – 2.38 | 0.289 | 1.33 | 0.62 – 2.88 | 0.465 | 1.69 | 0.72 – 4.19 | 0.237 |
| **Educational Attainment** |  |  |  |  |  |  |  |  |  |
| High school or less | Reference | |  |  |  |  |  |  |  |
| Some college/university | 0.72 | 0.34 – 1.39 | 0.347 | 0.67 | 0.24 – 1.65 | 0.410 | 0.60 | 0.18 – 1.64 | 0.349 |
| Completed college/university | 1.00 | 0.68 – 1.50 | 0.999 | 1.01 | 0.59 – 1.77 | 0.972 | 0.95 | 0.52 – 1.78 | 0.877 |
| Post-graduate/professional degree | 0.91 | 0.50 – 1.61 | 0.755 | 0.98 | 0.44 – 2.11 | 0.960 | 1.12 | 0.42 – 2.81 | 0.809 |
| **Primary language spoken** |  |  |  |  |  |  |  |  |  |
| English | Reference | |  |  |  |  |  |  |  |
| Other | 1.88 | 1.21 – 2.88 | **0.004** | 2.22 | 1.22 – 3.94 | **0.007** | 1.58 | 0.78 – 3.11 | 0.196 |
| **Self-perceived health** |  |  |  |  |  |  |  |  |  |
| Poor | Reference | |  |  |  |  |  |  |  |
| Fair | 0.89 | 0.45 – 1.85 | 0.743 | 1.08 | 0.39 – 3.52 | 0.893 | 0.72 | 0.28 – 1.97 | 0.507 |
| Good | 0.63 | 0.33 – 1.29 | 0.184 | 0.40 | 0.15 – 1.29 | 0.092 | 0.92 | 0.38 – 2.39 | 0.849 |
| Very good | 0.46 | 0.23 – 0.95 | **0.029** | 0.38 | 0.14 – 1.21 | 0.073 | 0.42 | 0.15 – 1.25 | 0.109 |
| Excellent | 0.47 | 0.20 – 1.08 | 0.069 | 0.34 | 0.11 – 1.20 | 0.074 | 0.65 | 0.15 – 2.40 | 0.527 |
| **ADG Score** |  |  |  |  |  |  |  |  |  |
| < 3 | Reference | |  |  |  |  |  |  |  |
| 3 – 4 | 0.65 | 0.35 – 1.24 | 0.181 | 0.72 | 0.36 – 1.48 | 0.357 | 0.32 | 0.06 – 1.82 | 0.167 |
| 5 – 6 | 0.62 | 0.33 – 1.17 | 0.129 | 0.75 | 0.37 – 1.57 | 0.430 | 0.33 | 0.08 – 1.67 | 0.135 |
| 7 – 8 | 0.61 | 0.32 – 1.17 | 0.124 | 0.87 | 0.40 – 1.91 | 0.724 | 0.28 | 0.07 – 1.42 | 0.086 |
| ≥ 9 | 0.82 | 0.45 – 1.55 | 0.520 | 0.80 | 0.33 – 1.87 | 0.603 | 0.58 | 0.16 – 2.77 | 0.436 |
| **Program type** |  |  |  |  |  |  |  |  |  |
| Enhanced FFS | Reference | |  |  |  |  |  |  |  |
| Capitation | 0.78 | 0.53 – 1.14 | 0.190 | 0.83 | 0.48 – 1.49 | 0.528 | 0.65 | 0.36 – 1.15 | 0.137 |
| Other | 0.59 | 0.09 – 2.09 | 0.481 | 0.50 | 0.03 – 2.84 | 0.520 | 0.60 | 0.03 – 3.80 | 0.655 |
| **Years with provider** |  |  |  |  |  |  |  |  |  |
| < 4 | Reference | |  |  |  |  |  |  |  |
| 4 – 9 | 0.93 | 0.56 – 1.54 | 0.766 | 0.73 | 0.38 – 1.39 | 0.338 | 1.36 | 0.58 – 3.34 | 0.485 |
| 10 – 19 | 1.22 | 0.75 – 1.98 | 0.426 | 1.17 | 0.64 – 2.18 | 0.612 | 1.47 | 0.65 – 3.49 | 0.367 |
| ≥ 20 | 1.07 | 0.67 – 1.72 | 0.793 | 0.86 | 0.47 – 1.60 | 0.639 | 1.57 | 0.71 – 3.69 | 0.281 |
| **RIO category** |  |  |  |  |  |  |  |  |  |
| Large urban | Reference | |  |  |  |  |  |  |  |
| Urban | 1.06 | 0.70 – 1.59 | 0.792 | 0.99 | 0.56 – 1.73 | 0.980 | 1.14 | 0.59 – 2.14 | 0.694 |
| Small urban | 0.99 | 0.56 – 1.72 | 0.970 | 1.14 | 0.56 – 2.31 | 0.723 | 0.83 | 0.30 – 2.14 | 0.700 |
| Rural | 1.03 | 0.48 – 2.15 | 0.940 | 1.01 | 0.38 – 2.55 | 0.986 | 1.30 | 0.33 – 4.54 | 0.692 |
| **Dependency** |  |  |  |  |  |  |  |  |  |
| 1^st^ quintile *(least marginalized)* | Reference | |  |  |  |  |  |  |  |
| 2^nd^ quintile | 1.23 | 0.70 – 2.14 | 0.467 | 1.08 | 0.54 – 2.15 | 0.829 | 1.55 | 0.59 – 4.17 | 0.372 |
| 3^rd^ quintile | 1.32 | 0.74 – 2.35 | 0.342 | 0.76 | 0.35 – 1.64 | 0.488 | 3.20 | 1.28 – 8.42 | **0.015** |
| 4^th^ quintile | 1.74 | 0.98 – 3.11 | 0.057 | 1.30 | 0.63 – 2.70 | 0.478 | 3.02 | 1.15 – 8.30 | **0.027** |
| 5^th^ quintile *(most marginalized)* | 1.54 | 0.86 – 2.79 | 0.150 | 1.01 | 0.47 – 2.18 | 0.985 | 3.13 | 1.20 – 8.61 | **0.022** |
| **Material Deprivation** |  |  |  |  |  |  |  |  |  |
| 1^st^ quintile *(least marginalized)* | Reference | |  |  |  |  |  |  |  |
| 2^nd^ quintile | 1.07 | 0.65 – 1.79 | 0.785 | 0.89 | 0.45 – 1.76 | 0.728 | 1.41 | 0.62 – 3.25 | 0.414 |
| 3^rd^ quintile | 0.98 | 0.56 – 1.70 | 0.942 | 1.27 | 0.64 – 2.52 | 0.489 | 0.54 | 0.19 – 1.44 | 0.223 |
| 4^th^ quintile | 1.48 | 0.86 – 2.57 | 0.155 | 1.83 | 0.91 – 3.75 | 0.093 | 1.17 | 0.48 – 2.91 | 0.724 |
| 5^th^ quintile *(most marginalized)* | 0.86 | 0.45 – 1.64 | 0.652 | 0.68 | 0.26 – 1.68 | 0.410 | 0.97 | 0.36 – 2.60 | 0.950 |
| **Ethnic Concentration** |  |  |  |  |  |  |  |  |  |
| 1^st^ quintile *(least marginalized)* | Reference | |  |  |  |  |  |  |  |
| 2^nd^ quintile | 0.82 | 0.45 – 1.46 | 0.495 | 0.81 | 0.39 – 1.66 | 0.573 | 0.82 | 0.28 – 2.33 | 0.712 |
| 3^rd^ quintile | 0.79 | 0.41 – 1.49 | 0.464 | 0.82 | 0.36 – 1.83 | 0.627 | 0.81 | 0.27 – 2.45 | 0.711 |
| 4^th^ quintile | 1.04 | 0.53 – 2.03 | 0.918 | 0.91 | 0.38 – 2.17 | 0.833 | 1.21 | 0.41 – 3.80 | 0.732 |
| 5^th^ quintile *(most marginalized)* | 1.73 | 0.88 – 3.46 | 0.115 | 1.37 | 0.57 – 3.39 | 0.483 | 2.82 | 0.92 – 9.25 | 0.076 |
| **Residential Instability** |  |  |  |  |  |  |  |  |  |
| 1^st^ quintile *(least marginalized)* | Reference | |  |  |  |  |  |  |  |
| 2^nd^ quintile | 0.76 | 0.44 – 1.30 | 0.322 | 0.59 | 0.28 – 1.22 | 0.162 | 1.06 | 0.45 – 2.48 | 0.899 |
| 3^rd^ quintile | 0.85 | 0.50 – 1.46 | 0.567 | 1.01 | 0.51 – 2.00 | 0.975 | 0.64 | 0.24 – 1.62 | 0.349 |
| 4^th^ quintile | 0.72 | 0.40 – 1.27 | 0.263 | 0.67 | 0.30 – 1.45 | 0.309 | 0.87 | 0.36 – 2.12 | 0.762 |
| 5^th^ quintile *(most marginalized)* | 0.82 | 0.47 – 1.41 | 0.464 | 0.91 | 0.43 – 1.89 | 0.790 | 0.68 | 0.28 – 1.63 | 0.386 |
