# Supplementary material for: The relationship between patient experience and real-world digital health access in primary care: A population-based cross-sectional study
Source: PLoS One. 2024 May 7;19(5):e0299005. doi: 10.1371/journal.pone.0299005 (PMC11075820; doi:10.1371/journal.pone.0299005)
Supplement: S5 Appendix — (DOCX) [file pone.0299005.s005.docx]

#load library ----

library(sas7bdat)

library(dplyr)

library(tidyr)

library(Hmisc)

library(psych)

library(mice)

library(VIM)

library(ggplot2)

library(xtable)

library(knitr)

library(finalfit)

library(sjPlot)

library(MASS)

library(car)

library(lme4)

library(nlme)

library(gridExtra)

library(sjstats)

library(sjlabelled)

library(pROC)

library(MASS)

library(caret)

library(gridExtra)

library(grid)

library(sandwich)

library(clubSandwich)

library(aod)

library(ordinal)

library(VGAM)

library(ResourceSelection)

library(brant)

library(ggpubr)

library(scales)

library(forestplot)

#load data ----

df <- read.sas7bdat(file = REDACTED)

df2 <- df

summary(df2)

#subset by wave ----

df2 <- subset(df2, wave == "27" | wave == "28" | wave == "29")

df2 <- subset(df2, study != 1 | is.na(study))

#omit responses without primary care physician ----

df2$physnum[df2$physnum == ""] <- NA

primary <- df2[,c("physnum")]

df2 <- df2[complete.cases(primary),]

#subset famdoc (1: has primary care provider)

df2 <- subset(df2, famdoc == 1)

#subset by patients seen provider at least once in past year

df2 <- subset(df2, num_encounters_1yr > 0)

#recoding (_.) ----

#date/wave reformat

df2$intdate. <- as.Date(df2$intdate, origin = "1960-01-01")

##PRIMARY EXPOSURES ----

#communicate through phone

df2$access_1[df2$access_1 == 8] <- NA

df2$access_1. <- factor(df2$access_1,

levels = c(1,2),

labels = c("Yes","No"))

#communicate through other means - HCES

df2$access_13[df2$access_13 == "NaN" & df2$wave == 27 & df2$study == 2] <- 5

df2$access_13[df2$access_13 == "NaN" & df2$wave == 28] <- 5

df2$access_13[df2$access_13 == "NaN" & df2$wave == 29] <- 5

df2$access_13[df2$access_13 == c(8,9)] <- NA

df2$access_13. <- factor(df2$access_13,

levels = c(1,5),

labels = c("Yes","No"))

#video access - HCES

df2$access_11a[df2$access_11a == "NaN" & df2$wave == 27 & df2$study == 2] <- 5

df2$access_11a[df2$access_11a == "NaN" & df2$wave == 28] <- 5

df2$access_11a[df2$access_11a == "NaN" & df2$wave == 29] <- 5

df2$access_11a[df2$access_11a == c(8,9)] <- NA

df2$access_11a. <- factor(df2$access_11a,

levels = c(1,5),

labels = c("Yes","No"))

#messaging access - HCES

df2$access_12a[df2$access_12a == "NaN" & df2$wave == 27 & df2$study == 2] <- 5

df2$access_12a[df2$access_12a == "NaN" & df2$wave == 28] <- 5

df2$access_12a[df2$access_12a == "NaN" & df2$wave == 29] <- 5

df2$access_12a[df2$access_12a == c(8,9)] <- NA

df2$access_12a. <- factor(df2$access_12a,

levels = c(1,5),

labels = c("Yes","No"))

#health record (condition) - HCES

df2$dh_2[df2$dh_2 == "NaN" & df2$wave == 27 & df2$study == 2] <- 5

df2$dh_2[df2$dh_2 == "NaN" & df2$wave == 28] <- 5

df2$dh_2[df2$dh_2 == "NaN" & df2$wave == 29] <- 5

df2$dh_2[df2$dh_2 == c(8,9)] <- NA

df2$dh_2. <- factor(df2$dh_2,

levels = c(1,5),

labels = c("Yes","No"))

#health record (comprehensive) - HCES

df2$dh_4[df2$dh_4 == "NaN" & df2$wave == 27 & df2$study == 2] <- 5

df2$dh_4[df2$dh_4 == "NaN" & df2$wave == 28] <- 5

df2$dh_4[df2$dh_4 == "NaN" & df2$wave == 29] <- 5

df2$dh_4[df2$dh_4 == c(8,9)] <- NA

df2$dh_4. <- factor(df2$dh_4,

levels = c(1,5),

labels = c("Yes","No"))

#health record - HCES

df2$dh_1a[df2$dh_1a == "NaN" & df2$wave == 27 & df2$study == 2] <- 5

df2$dh_1a[df2$dh_1a == "NaN" & df2$wave == 28] <- 5

df2$dh_1a[df2$dh_1a == "NaN" & df2$wave == 29] <- 5

df2$dh_1a[df2$dh_1a == c(8,9)] <- NA

df2$dh_1a. <- factor(df2$dh_1a,

levels = c(1,5),

labels = c("Yes","No"))

#virtual care with other provider - HCES

df2$dh_5a[df2$dh_5a == "NaN" & df2$wave == 27 & df2$study == 2] <- 5

df2$dh_5a[df2$dh_5a == "NaN" & df2$wave == 28] <- 5

df2$dh_5a[df2$dh_5a == "NaN" & df2$wave == 29] <- 5

df2$dh_5a[df2$dh_5a == c(8,9)] <- NA

df2$dh_5a. <- factor(df2$dh_5a,

levels = c(1,5),

labels = c("Yes","No"))

#email access - HCES

df2$access_3[df2$access_3 == "NaN" & df2$wave == 27 & df2$study == 2] <- 5

df2$access_3[df2$access_3 == "NaN" & df2$wave == 28] <- 5

df2$access_3[df2$access_3 == "NaN" & df2$wave == 29] <- 5

df2$access_3[df2$access_3 == c(8,9)] <- NA

df2$access_3. <- factor(df2$access_3,

levels = c(1,5,6,7),

labels = c("Yes","No","No","No"))

#internet access - HCES

df2$access_3a[df2$access_3a == "NaN" & df2$wave == 27 & df2$study == 2] <- 5

df2$access_3a[df2$access_3a == "NaN" & df2$wave == 28] <- 5

df2$access_3a[df2$access_3a == "NaN" & df2$wave == 29] <- 5

df2$access_3a[df2$access_3a == c(8,9)] <- NA

df2$access_3a. <- factor(df2$access_3a,

levels = c(1,5),

labels = c("Yes", "No"))

#appointment booking - HCES

df2$access_4c[df2$access_4c == "NaN" & df2$wave == 27 & df2$study == 2] <- 5

df2$access_4c[df2$access_4c == "NaN" & df2$wave == 28] <- 5

df2$access_4c[df2$access_4c == "NaN" & df2$wave == 29] <- 5

df2$access_4c[df2$access_4c == c(8,9)] <- NA

df2$access_4c. <- factor(df2$access_4c,

levels = c(5,1),

labels = c("No","Yes"))

#answered?

df2$access_4c[is.na(df2$access_4c)] <- "Missing"

df2 = df2 %>% mutate(booka = ifelse(access_4c == 1 | access_4c == 5, "Answered", "Missing"))

df2$booka. <- factor(df2$booka)

#overal digital health use

df2 = df2 %>% mutate(dhu = ifelse(access_11a. == "Yes" | access_12a. == "Yes" | dh_2. == "Yes" | dh_4. == "Yes" |

dh_1a. == "Yes" | dh_5a. == "Yes" | access_3. == "Yes" | access_3a. == "Yes" |

access_4c. == "Yes", "Digital health", "No digital health"))

df2$dhu. <- factor(df2$dhu, levels = c("No digital health","Digital health"))

#answered?

df2$dhu[is.na(df2$dhu)] <- "Missing"

df2 = df2 %>% mutate(dhua = ifelse(dhu == "Digital health" | dhu == "No digital health", "Answered", "Missing"))

df2$dhua. <- factor(df2$dhua)

#overall remote communication

df2 = df2 %>% mutate(rc = ifelse(access_1. == "Yes" | access_11a. == "Yes" | access_3. == "Yes" | access_3a. == "Yes" | access_12a. == "Yes" |

dh_5a. == "Yes" | access_13. == "Yes", "Remote communication","No remote communication"))

df2$rc. <- factor(df2$rc, levels = c("No remote communication","Remote communication"))

#answered?

df2$rc[is.na(df2$rc)] <- "Missing"

df2 = df2 %>% mutate(rca = ifelse(rc == "Remote communication" | rc == "No remote communication", "Answered", "Missing"))

df2$rca. <- factor(df2$rca)

#overall health record

df2 = df2 %>% mutate(hr = ifelse(dh_2. == "Yes" | dh_4. == "Yes" | dh_1a. == "Yes", "Health record", "No health record"))

df2$hr. <- factor(df2$hr, levels = c("No health record","Health record"))

#answered?

df2$hr[is.na(df2$hr)] <- "Missing"

df2 = df2 %>% mutate(hra = ifelse(hr == "Health record use" | hr == "No health record use", "Answered", "Missing"))

df2$hra. <- factor(df2$hra)

#PRIMARY OUTCOME ----

#doctor knows medical history

df2$exp_1[df2$exp_1 == c(7,8,9)] <- NA

df2$exp_1. <- factor(df2$exp_1,

levels = c(1,2,3,4,5,6),

labels = c("Always","Often","Sometimes","Rarely","Never","Depends"))

#doctor gives oppurtunity to ask questions

df2$exp_2[df2$exp_2 == c(7,8,9)] <- NA

df2$exp_2. <- factor(df2$exp_2,

levels = c(1,2,3,4,5,6),

labels = c("Always","Often","Sometimes","Rarely","Never","Depends"))

#Doctor spends enough time with you

df2$exp_3[df2$exp_3 == c(7,8,9)] <- NA

df2$exp_3. <- factor(df2$exp_3,

levels = c(1,2,3,4,5,6),

labels = c("Always","Often","Sometimes","Rarely","Never","Depends"))

#Doctor involves patient in decision making

df2$exp_4[df2$exp_4 == c(7,8,9)] <- NA

df2$exp_4. <- factor(df2$exp_4,

levels = c(1,2,3,4,5,6),

labels = c("Always","Often","Sometimes","Rarely","Never","Depends"))

#doctor explanations are easy to understand

df2$exp_5[df2$exp_5 == c(7,8,9)] <- NA

df2$exp_5. <- factor(df2$exp_5,

levels = c(1,2,3,4,5,6),

labels = c("Always","Often","Sometimes","Rarely","Never","Depends"))

#ordinal groups

df2 = df2 %>% mutate(exp_recode_1. = ifelse(exp_recode_1 <= 3, "Sometimes/Rarely/Never",

ifelse(exp_recode_1 == 4, "Often", "Always")))

df2$exp_recode_1. <- factor(df2$exp_recode_1., ordered = T)

levels(df2$exp_recode_1.)

df2 = df2 %>% mutate(exp_recode_2. = ifelse(exp_recode_2 <= 3, "Sometimes/Rarely/Never",

ifelse(exp_recode_2 == 4, "Often", "Always")))

df2$exp_recode_2. <- factor(df2$exp_recode_2., ordered = T)

levels(df2$exp_recode_2.)

df2 = df2 %>% mutate(exp_recode_3. = ifelse(exp_recode_3 <= 3, "Sometimes/Rarely/Never",

ifelse(exp_recode_3 == 4, "Often", "Always")))

df2$exp_recode_3. <- factor(df2$exp_recode_3., ordered = T)

levels(df2$exp_recode_3.)

df2 = df2 %>% mutate(exp_recode_4. = ifelse(exp_recode_4 <= 3, "Sometimes/Rarely/Never",

ifelse(exp_recode_4 == 4, "Often", "Always")))

df2$exp_recode_4. <- factor(df2$exp_recode_4., ordered = T)

df2 = df2 %>% mutate(exp_recode_5. = ifelse(exp_recode_5 <= 3, "Sometimes/Rarely/Never",

ifelse(exp_recode_5 == 4, "Often", "Always")))

df2$exp_recode_5. <- factor(df2$exp_recode_5., ordered = T)

levels(df2$exp_recode_5.)

#consistent experience responses

df2 = df2 %>% mutate(exp_con = ifelse(exp_recode_1. == "Sometimes/Rarely/Never" & exp_recode_2. == "Sometimes/Rarely/Never" &

exp_recode_3. == "Sometimes/Rarely/Never" & exp_recode_4. == "Sometimes/Rarely/Never" &

exp_recode_5. == "Sometimes/Rarely/Never", "Consistent", "Other"))

##CONFOUNDER/EFFECT MODIFIERS ----

#age - RPDB

df2$age_rpdb[df2$age_rpdb == c(1)] <- NA

df2$age_rpdb[df2$age_rpdb == c(14)] <- NA

df2$age_rpdb[df2$age_rpdb == c(15)] <- NA

df2 = df2 %>% mutate(agec. = ifelse(age_rpdb <= 44, "16-44",

ifelse(age_rpdb >= 45 & age_rpdb <= 64, "45-64", "65+")))

df2$agec. <- factor(df2$agec.)

#sex - RPDB

df2$sex_rpdb. <- factor(df2$sex_rpdb)

summary(df2$sex_rpdb.)

#education status - HCES

df2$edu[df2$edu == c(98,99)] <- NA

df2 = df2 %>% mutate(edu. = ifelse(edu <= 3, "Highschool or less",

ifelse(edu == 4 | edu == 6, "Some college/undergraduate",

ifelse(edu == 5 | edu == 7, "College/undergraduate degree",

"Postgraduate/professional degree"))))

df2$edu. <- factor(df2$edu.)

df2 <- within(df2, edu. <- relevel(edu., ref = 2))

levels(df2$edu.)

#lhin - HCES

df2$lhin. <- factor(df2$lhin,

levels = c(1,2,3,4,5,6,7,8,9,10,11,12,13,14),

labels = c("Erie St Clair","South West","Waterloo Wellington",

"Hamilton Niagara Haldimand Brant","Central West",

"Mississauga Halton","Toronto Central","Central","Central East",

"South East","Champlain","North Simcoe Muskoka","North East",

"North West"))

#prog type -

df2$progtype[df2$progtype == c("")] <- NA

levels(df2$progtype) <- list(Other = "COE", Other = "CSA", Other = "HIV", Other = "SLA",

Other = "SMO", Other = "STJ", EnhancedFFS = "CCM", EnhancedFFS = "FHG",

Capitation = "FHN", Capitation = "FHO", Other = "GHC", Other = "RAN")

df2$progtype. <- df2$progtype

df2$progtype. <- relevel(df2$progtype., ref = "EnhancedFFS")

#practice type

df2$practype. <- as.character(df2$practype)

df2$practype.[df2$practype. == ""] <- NA

df2$practype.[df2$practype. == "specialist"] <- NA

df2$practype.[df2$practype. == "<44 days"] <- "other"

df2$practype.[df2$practype. == "focused"] <- "other"

df2$practype. <- factor(df2$practype.)

df2$practype. <- relevel(df2$practype., ref = "other")

#years with current doc

df2$fd_5[df2$fd_5 == c(0)] <- NA

df2$fd_5[df2$fd_5 == c(81)] <- NA

df2$fd_5[df2$fd_5 == c(98)] <- NA

df2$fd_5[df2$fd_5 == c(99)] <- NA

df2 = df2 %>% mutate(fdy. = ifelse(fd_5 < 4, "3 or less",

ifelse(fd_5 >= 4 & fd_5 < 10, "4-9",

ifelse(fd_5 >= 10 & fd_5 < 20, "10-19",

"20 or more"))))

df2$fdy. <- factor(df2$fdy.)

df2$fdy. <- relevel(df2$fdy., ref = "3 or less")

df2$fdy <- df2$fd_5

#language spoken

df2$lang_2[df2$lang_2 == c(98)] <- NA

df2$lang_2[df2$lang_2 == c(99)] <- NA

df2 = df2 %>% mutate(lang. = ifelse(lang_2 == 1, "English", "Other"))

df2$lang. <- factor(df2$lang.)

#rurality - RPDB

df2$rural. <- factor(df2$rural_rpdb)

#more in depth breakdown - RPDB

df2$rurality. <- df2$rio2008_rpdb

df2 = df2 %>% mutate(rurality = ifelse(rio2008_rpdb == 0, "Large urban",

ifelse(rio2008_rpdb >= 1 & rio2008_rpdb < 10, "Urban",

ifelse(rio2008_rpdb >= 10 & rio2008_rpdb <40, "Small urban",

"Rural"))))

df2$rurality. <- factor(df2$rurality)

#satisfaction of care in the community

df2$w1[df2$w1 == c(8,9)] <- NA

df2$w1. <- factor(df2$w1,

levels = c(4,3,2,1),

labels = c("Not satisfied at all","Not very satisfied", "Somewhat satisfied",

"Very satisfied"))

#Income - HCES ----

#financial situtation

df2$fin_sit[df2$fin_sit == c(8)] <- NA

df2$fin_sit[df2$fin_sit == c(9)] <- NA

df2 = df2 %>% mutate(fin_sit. = ifelse(fin_sit == 1, "Very comfortable",

ifelse(fin_sit == 2, "Comfortable", "Tight/Very tight/Poor")))

df2$fin_sit. <- factor(df2$fin_sit.)

df2$fin_sit. <- relevel(df2$fin_sit., ref = "Very comfortable")

#income

df2$inc_cat[df2$inc_cat == c(98,99)] <- NA

df2 = df2 %>% mutate(inc_cat. = ifelse(inc_cat == 1, "<$20k",

ifelse(inc_cat == 2 | inc_cat == 3, "$20k-<$40k",

ifelse(inc_cat == 4 | inc_cat == 5, "$40k-<$60k",

ifelse(inc_cat == 6 | inc_cat == 7, "$60k-<$80k",

ifelse(inc_cat == 8 | inc_cat == 9, "$80k-<$100k",

ifelse(inc_cat == 10 | inc_cat == 11, "$100k-<$150k",

">150k")))))))

df2$inc_cat. <- factor(df2$inc_cat.)

#Marginilization ----

#dependency - ONMARG

df2$dependency_q_da[is.nan(df2$dependency_q_da)] <- NA

df2$dependency. <- factor(df2$dependency_q_da)

#deprivation - ONMARG

df2$deprivation_q_da[is.nan(df2$deprivation_q_da)] <- NA

df2$deprivation. <- factor(df2$deprivation_q_da)

#ethnic concentration - ONMARG

df2$ethniccon_q_da[is.nan(df2$ethniccon_q_da)] <- NA

df2$ethnic. <- factor(df2$ethniccon_q_da)

#instability - ONMARG

df2$instability_q_da[is.nan(df2$instability_q_da)] <- NA

df2$instability. <- factor(df2$instability_q_da)

#correlation between scores

onmarg <- data.frame(df2$dependency_da, df2$deprivation_da, df2$ethniccon_da, df2$instability_da)

cor(onmarg, use = "complete.obs")

#summary margin. score

df2$margs. <- (df2$dependency_q_da + df2$deprivation_q_da + df2$ethniccon_q_da + df2$instability_q_da)/4

#chronic conditions ----

#chronic - HCES

#respondants health - HCES

df2$rh_1[df2$rh_1 == c(8,9)] <- NA

df2$rh_1. <- factor(df2$rh_1,

levels = c(5,4,3,2,1),

labels = c("Poor","Fair","Good","Very good","Excellent"))

#high bp/hypertension - HCES

df2$rh_2a[df$rh_2a == c(8,9)] <- NA

df2$rh_2a. <- factor(df2$rh_2a,

levels = c(1,5),

labels = c("Yes","No"))

#diabetes - HCES

df2$rh_2b[df2$rh_2b == c(8,9)] <- NA

df2$rh_2b. <- factor(df2$rh_2b,

levels = c(1,5),

labels = c("Yes","No"))

#arthritis - HCES

df2$rh_2c[df2$rh_2c == c(8,9)] <- NA

df2$rh_2c. <- factor(df2$rh_2c,

levels = c(1,5),

labels = c("Yes","No"))

#heart attack, heart disease - HCES

df2$rh_2d[df2$rh_2d == c(8,9)] <- NA

df2$rh_2d. <- factor(df2$rh_2d,

levels = c(1,5),

labels = c("Yes","No"))

#cancer - HCES

df2$rh_2e[df2$rh_2e == c(8,9)] <- NA

df2$rh_2e. <- factor(df2$rh_2e,

levels = c(1,5),

labels = c("Yes","No"))

#asthma - ASTHMA

df2$asthma. <- factor(df2$asthma,

levels = c(0,1),

labels = c("No","Yes"))

#congestive heart failure - CHF

df2$chf. <- factor(df2$chf,

levels = c(0,1),

labels = c("No","Yes"))

#chronic obstructive pulmonary disease - COPD

df2$copd. <- factor(df2$copd,

levels = c(0,1),

labels = c("No","Yes"))

#dementia - DEMENTIA

df2$dementia. <- factor(df2$dementia,

levels = c(0,1),

labels = c("No","Yes"))

#HIV - HIV

df2$hiv. <- factor(df2$hiv,

levels = c(0,1),

labels = c("No","Yes"))

#hypertension - HYPER

df2$hyper. <- factor(df2$hyper,

levels = c(0,1),

labels = c("No","Yes"))

#diabetes - ODD

df2$diabetes. <- factor(df2$diabetes,

levels = c(0,1),

labels = c("No","Yes"))

#rheumatoid arthitis - ORAD

df2$rheum_arth. <- factor(df2$rheum_arth,

levels = c(0,1),

labels = c("No","Yes"))

#crohns disease - OCCC

df2$crohns. <- factor(df2$crohns,

levels = c(0,1),

labels = c("No","Yes"))

#ulcertative colitis - UCCC

df2$ulcerative_colitis. <- factor(df2$ulcerative_colitis,

levels = c(0,1),

labels = c("No","Yes"))

#heart attack - OMID

df2$omid_ha. <- factor(df2$omid_ha,

levels = c(0,1),

labels = c("No","Yes"))

#heart attack - DAD, NACRS, OHIP

df2$heart_attack. <- factor(df2$heart_attack,

levels = c(0,1),

labels = c("No","Yes"))

#comorbidities summary variable ----

df2 = df2 %>% mutate(cancer_n = ifelse(rh_2e. == "Yes", 1, 0))

df2$comorb <- df2$asthma + df2$chf + df2$copd + df2$dementia + df2$hiv + df2$hyper + df2$diabetes + df2$rheum_arth + df2$crohns + df2$ulcerative_colitis +

df2 = df2 %>% mutate(comorb. = ifelse(comorb < 1, "No chronic condition",

ifelse(comorb == 1, "1 chronic condition",

ifelse(comorb == 2, "2 comorbidities",

"3 or more comorbidities"))))

df2$comorb. <- factor(df2$comorb.)

df2$comorb. <- relevel(df2$comorb., ref="No chronic condition")

#ADG Score ----

quantile(df2$ADG_score, probs = seq(0,1,.2))

df2 = df2 %>% mutate(ADG. = ifelse(ADG_score < 3, "< 3",

ifelse(ADG_score >= 3 & ADG_score < 5, "3-4",

ifelse(ADG_score >= 5 & ADG_score < 7, "5-6",

ifelse(ADG_score >= 7 & ADG_score < 9, "7-8",

"> 9")))))

df2$ADG. <- factor(df2$ADG.)

#assigning waves by int. date ----

df2$wave. <- factor(df2$wave)

#imputing income using deprivation ----

df3 <- df2

init = mice(df3, maxit = 0)

meth = init$method

predM = init$predictorMatrix

predM[,c(1:348, 350:457)] = 0

meth[c(1:428, 430:457)] = ""

meth[c("inc_cat.")] = "polyreg"

set.seed(12345)

imputed = mice(df3, method = meth, predictorMatrix = predM, m=5)

imputed <- complete(imputed)

sapply(imputed, function(x) sum(is.na(x)))

###MODEL BUILDING### ----

#remove NA's and subset by analyses ----

#a1: overall patient experience

#a2: time to urgent appointment

#a3: time to routine appointment

a1 <- df3[,c(90,297:301,303,304,307,313,347,349,351,353,355,357,358,385,389,393,397,415:421,423:425,427,428,430:453,455:457)]

a1 <- na.omit(a1)

s12 <- df3[,c(90,93,297:301,303,304,307,313,347,349,351,353,355,357,358,385,389,393,397,415:421,423:425,427:453,455:457)]

#descriptive statistics ----

s12 = s12 %>% mutate(expb = ifelse(oa_pat_exp <= 21, "Poorer experience","Positive experience"))

s12$expb. <- factor(s12$expb)

s12 = s12 %>% mutate(urgent2. = ifelse(sick_3 <= 1, "Same/next day", "Later day"))

s12$urgent2. <- factor(s12$urgent2.)

s12 = s12 %>% mutate(num_encounters_1yr. = ifelse(num_encounters_1yr <= 3, "<= 3 encounters","> 3 encounters"))

s12$num_encounters_1yr. <- factor(s12$num_encounters_1yr.)

#outcome plots

tv1 <- sum(a1$oa_pat_exp)

to1 <- nrow(a1)

o1 <- ggplot(a1, aes(oa_pat_exp, fill = ..x.. <= 21)) +

geom_histogram(binwidth = 1, colour = "black", size = 0.4) +

scale_fill_manual(values = c("#DCEEF3","#003366"),

labels = c("Positive experience","Poor experience")) +

labs(y = "Frequency (n)", x = "Overall patient experience score (/25)", fill = "Experience") +

geom_vline(xintercept = 21.5, linetype = "dotted") +

scale_y_continuous(sec.axis = sec_axis(trans = ~./to1, labels = percent,

name = "Proportion (%)")) +

theme_bw() +

theme(legend.justification = c(1,1), legend.position = c(0.35,0.98), legend.title = element_blank())

##A1 ANALYSIS## ----

#potential multilevel

#level a: patient characteristics

#level b: provider characteristics: practype, progtype, physnum

#level c: area charateristics: FSA, onmarg?, lhin

#quantiles: quartiles, quintiles & deciles

quantile(a1$oa_pat_exp, probs = seq(0,1,1/4))

quantile(a1$oa_pat_exp, probs = seq(0,1,1/5))

quantile(a1$oa_pat_exp, probs = seq(0,1,1/10))

#distinguish low-end of experience

a1 = a1 %>% mutate(expb = ifelse(oa_pat_exp <= 21, "Poorer experience","Positive experience"))

a1$expb. <- factor(a1$expb)

a1$expb2. <- relevel(a1$expb., "Positive experience")

#univariable logistic regression

#plots ----

dhlab <- c("No","Yes")

p1 <- ggplot(a1, aes(x = rc., fill = expb.)) +

geom_bar(position = "fill") +

labs(y = "Proportion",

x = "Remote communication") +

scale_y_continuous(labels = scales::percent) +

theme_linedraw() +

scale_x_discrete(labels = dhlab)

p2 <- ggplot(a1, aes(x = hr., fill = expb.)) +

geom_bar(position = "fill") +

labs(y = "Proportion",

x = "Health record use") +

scale_y_continuous(labels = scales::percent) +

scale_x_discrete(labels = dhlab) +

theme_linedraw()

p3 <- ggplot(a1, aes(x = access_4c., fill = expb.)) +

geom_bar(position = "fill") +

labs(y = "Proportion",

x = "Online booking use") +

scale_y_continuous(labels = scales::percent) +

theme_linedraw()

pm1 <- ggarrange(p1,p2,p3, ncol = 3, nrow = 1, common.legend = TRUE, legend = "bottom")

#Bivariate analysis - chisq test ----

sjt.xtab(a1$expb2., a1$rc., show.col.prc = T, var.labels = c("Summed patient experience",

"Remote communication access"))

sjt.xtab(a1$expb2., a1$hr., show.col.prc = T, var.labels = c("Summed patient experience",

"Digital health record access"))

sjt.xtab(a1$expb2., a1$access_4c., show.col.prc = T, var.labels = c("Summed patient experience",

"Online booking access"))

#models ----

#null

a1nmodel1 <- glm(expb. ~ 1, data = a1, family = "binomial")

tab_model(a1nmodel1)

a1nmodel2 <- glmer(expb. ~ 1 + (1 | physnum),

data = a1, family = "binomial",

control = glmerControl(optimizer = "bobyqa"),

nAGQ = 1)

tab_model(a1nmodel2)

a1nmodel3 <- glmer(expb. ~ 1 + (1 | lhin.),

data = a1, family = "binomial",

control = glmerControl(optimizer = "bobyqa"),

nAGQ = 1)

tab_model(a1nmodel3)

anova(a1nmodel3,a1nmodel2)

anova(a1nmodel3,a1nmodel1)

a1nmodel4 <- glmer(expb. ~ 1 + (1 | fsa_rpdb),

data = a1, family = "binomial",

control = glmerControl(optimizer = "bobyqa"),

nAGQ = 1)

tab_model(a1nmodel4)

anova(a1nmodel4,a1nmodel2)

anova(a1nmodel4,a1nmodel1)

#model comparisons

a1nmodel1$aic

AIC(logLik(a1nmodel2))

AIC(logLik(a1nmodel3))

AIC(logLik(a1nmodel4))

performance::icc(a1nmodel2)

performance::icc(a1nmodel3)

performance::icc(a1nmodel4)

#univariable

modelau1 <- glm(expb. ~ rc., data = a1, family = "binomial")

summary(modelau1)

tab_model(modelau1)

modelau2 <- glm(expb. ~ hr., data = a1, family = "binomial")

summary(modelau2)

tab_model(modelau2)

modelau3 <- glm(expb. ~ access_4c., data = a1, family = "binomial")

summary(modelau3)

tab_model(modelau3)

#generalized linear mixed models (GLMM)/multilevel analysis

modela1 <- glm(expb. ~ rc. + hr. + access_4c. + age_rpdb + sex_rpdb. + edu. +

w1. + inc_cat. + rh_1. + asthma. + chf. + copd. + dementia. + hyper. +

diabetes. + rheum_arth. + crohns. + ulcerative_colitis. + heart_attack.,

data = a1, family = binomial)

summary(modela1)

tab_model(modela1)

vif(modela1)

modela1$aic

#remove hiv - problematic

modela2 <- glmer(expb. ~ rc. + hr. + access_4c. + age_rpdb + sex_rpdb. + edu. +

w1. + inc_cat. + rh_1. + asthma. + chf. + copd. + dementia. + hyper. +

diabetes. + rheum_arth. + crohns. + ulcerative_colitis. + heart_attack. +

(1 | physnum) + practype. + progtype., data = a1, family = binomial, control = glmerControl(optimizer = "bobyqa"),

nAGQ = 1)

print(modela2, corr = F)

performance::icc(modela2)

vif(modela2)

tab_model(modela2)

AIC(logLik(modela2))

#remove lhin

modela3 <- glmer(expb. ~ rc. + hr. + access_4c. + age_rpdb + sex_rpdb. + edu. +

w1. + inc_cat. + rh_1. + asthma. + chf. + copd. + dementia. + hyper. +

diabetes. + rheum_arth. + crohns. + ulcerative_colitis. + heart_attack. +

(1 | physnum) + practype. + progtype. + (1 | fsa_rpdb) + dependency. + deprivation. +

ethnic. + instability. + rurality., data = a1, family = binomial, control = glmerControl(optimizer = "bobyqa"),

nAGQ = 1)

print(modela3, corr = F)

performance::icc(modela3)

vif(modela3)

tab_model(modela3)

plot(modela3)

AIC(logLik(modela3))

#only 2 levels w/ lhin

#only digital health

modela4 <- glmer(expb. ~ rc. + hr. + access_4c. + (1 | lhin.),

data = a1, family = binomial, control = glmerControl(optimizer = "bobyqa"),

nAGQ = 10)

print(modela4, corr = F)

performance::icc(modela4)

vif(modela4)

tab_model(modela4)

plot(modela4)

AIC(logLik(modela4))

#include personal characteristics

modela5 <- glmer(expb. ~ rc. + hr. + access_4c. + age_rpdb +

(1 | lhin.), data = a1, family = binomial,

control = glmerControl(optimizer = "bobyqa"),

nAGQ = 10)

AIC(logLik(modela5))

anova(modela5,modela4)

modela6 <- glmer(expb. ~ rc. + hr. + access_4c. + age_rpdb + sex_rpdb.

+ (1 | fsa_rpdb), data = a1, family = binomial,

control = glmerControl(optimizer = "bobyqa"),

nAGQ = 1)

AIC(logLik(modela6))

anova(modela6,modela5, test = "LRT")

modela7 <- glmer(expb. ~ rc. + hr. + access_4c. + age_rpdb + sex_rpdb. + edu. +

(1 | fsa_rpdb), data = a1, family = binomial,

control = glmerControl(optimizer = "bobyqa"),

nAGQ = 1)

AIC(logLik(modela7))

anova(modela7,modela6, test = "LRT")

modela8 <- glmer(expb. ~ rc. + hr. + access_4c. + age_rpdb + sex_rpdb. + edu. + inc_cat. +

(1 | fsa_rpdb), data = a1, family = binomial,

control = glmerControl(optimizer = "bobyqa"),

nAGQ = 1)

AIC(logLik(modela8))

anova(modela8,modela7, test = "LRT")

modela9 <- glmer(expb. ~ rc. + hr. + access_4c. + age_rpdb + sex_rpdb. + edu. + inc_cat. + w1. +

(1 | fsa_rpdb), data = a1, family = binomial,

control = glmerControl(optimizer = "bobyqa"),

nAGQ = 1)

AIC(logLik(modela9))

anova(modela9,modela8, test = "LRT")

tab_model(modela9)

vif(modela9)

Anova()

anova(modela9, test = "Chisq")

modela9 <- glm(expb. ~ rc. + hr. + access_4c. + age_rpdb + sex_rpdb. + edu. + inc_cat. + w1.,

data = a1, family = binomial)

modela4 <- glmer(expb. ~ rc. + hr. + access_4c. + age_rpdb + sex_rpdb. + edu. +

inc_cat. + rh_1. + num_encounters_1yr +

progtype. + practype. + (1 | lhin.) + dependency_da +

deprivation_da + ethniccon_da + instability_da,

data = a1, family = binomial, control = glmerControl(optimizer = "bobyqa"),

nAGQ = 1)

print(modela4, corr = F)

performance::icc(modela4)

vif(modela4)

tab_model(modela4)

plot(modela4)

AIC(logLik(modela4))

#multivariable logistic regression - expb. ----

#buidling hiearchal model

#level 1: digital health use

#level 2: patient characteristics (age, sex, income, education, rurality, onmarg)

#level 3: patient health conditions

#level 4: healthcare use (satisfaction with healthcare, program type, number of

#encounters in past 1yr, practice type)

#only digital health predictors

modela3 <- glm(expb. ~ rc. + hr. + access_4c., data = a1, family = "binomial")

tab_model(modela3)

vif(modela3)

#patient characteristics

modela4 <- glm(expb. ~ rc. + hr. + access_4c. + age_rpdb,

data = a1, family = "binomial")

anova(modela3, modela4, test = "LRT") #significant

modela5 <- glm(expb. ~ rc. + hr. + access_4c. + age_rpdb + sex_rpdb.,

data = a1, family = "binomial")

anova(modela4, modela5, test = "LRT") #not significant

modela6 <- glm(expb. ~ rc. + hr. + access_4c. + age_rpdb + sex_rpdb.,

data = a1, family = "binomial")

anova(modela4, modela5, test = "LRT") #not significant

modela6 <- glm(expb. ~ rc. + hr. + access_4c. + age_rpdb + sex_rpdb. + inc_cat.,

data = a1, family = "binomial")

anova(modela5, modela6, test = "LRT") #significant

modela7 <- glm(expb. ~ rc. + hr. + access_4c. + age_rpdb + sex_rpdb. + inc_cat. + edu.,

data = a1, family = "binomial")

anova(modela6, modela7, test = "LRT") #not significant

modela8 <- glm(expb. ~ rc. + hr. + access_4c. + age_rpdb + sex_rpdb. + inc_cat. + edu. + w1.,

data = a1, family = "binomial")

anova(modela7, modela8, test = "LRT") #significant

modela9 <- glm(expb. ~ rc. + hr. + access_4c. + age_rpdb + sex_rpdb. + inc_cat. + edu. + w1. +

dependency_da,

data = a1, family = "binomial")

anova(modela8, modela9, test = "LRT") #not significant

modela10 <- glm(expb. ~ rc. + hr. + access_4c. + age_rpdb + sex_rpdb. + inc_cat. + edu. + w1. +

dependency_da + deprivation_da,

data = a1, family = "binomial")

anova(modela9, modela10, test = "LRT") #not significant

modela11 <- glm(expb. ~ rc. + hr. + access_4c. + age_rpdb + sex_rpdb. + inc_cat. + edu. + w1. +

dependency_da + deprivation_da + ethniccon_da,

data = a1, family = "binomial")

anova(modela10, modela11, test = "LRT") #significant

modela12 <- glm(expb. ~ rc. + hr. + access_4c. + age_rpdb + sex_rpdb. + inc_cat. + edu. + w1. +

dependency_da + deprivation_da + ethniccon_da + instability_da,

data = a1, family = "binomial")

anova(modela11, modela12, test = "LRT") #not significant

modela13 <- glm(expb. ~ rc. + hr. + access_4c. + age_rpdb + sex_rpdb. + inc_cat. + edu. + w1. +

dependency_da + deprivation_da + ethniccon_da + instability_da + rurality.,

data = a1, family = "binomial")

anova(modela12, modela13, test = "LRT") #not significant

#overall examination

anova(modela13, test = "Chisq")

tab_model(modela13)

#patient health

modela14 <- glm(expb. ~ rc. + hr. + access_4c. + age_rpdb + sex_rpdb. + inc_cat. + edu. + w1. +

dependency_da + deprivation_da + ethniccon_da + instability_da + rurality. + rh_1.,

data = a1, family = "binomial")

anova(modela13, modela14, test = "LRT") # significant

modela15 <- glm(expb. ~ rc. + hr. + access_4c. + age_rpdb + sex_rpdb. + inc_cat. + edu. + w1. +

dependency_da + deprivation_da + ethniccon_da + instability_da + rurality. + rh_1. +

asthma., data = a1, family = "binomial")

anova(modela14, modela15, test = "LRT") #not significant

modela16 <- glm(expb. ~ rc. + hr. + access_4c. + age_rpdb + sex_rpdb. + inc_cat. + edu. + w1. +

dependency_da + deprivation_da + ethniccon_da + instability_da + urality. + rh_1. +

asthma. + chf., data = a1, family = "binomial")

anova(modela15, modela16, test = "LRT") #not significant

modela17 <- glm(expb. ~ rc. + hr. + access_4c. + age_rpdb + sex_rpdb. + inc_cat. + edu. + w1. +

dependency_da + deprivation_da + ethniccon_da + instability_da + rurality. + rh_1. +

asthma. + chf. + copd., data = a1, family = "binomial")

anova(modela16, modela17, test = "LRT") #not significant

modela18 <- glm(expb. ~ rc. + hr. + access_4c. + age_rpdb + sex_rpdb. + inc_cat. + edu. + w1. +

dependency_da + deprivation_da + ethniccon_da + instability_da + rurality. + rh_1. +

asthma. + chf. + copd. + dementia., data = a1, family = "binomial")

anova(modela17, modela18, test = "LRT") #not significant

modela19 <- glm(expb. ~ rc. + hr. + access_4c. + age_rpdb + sex_rpdb. + inc_cat. + edu. + w1. +

dependency_da + deprivation_da + ethniccon_da + instability_da + rurality. + rh_1. +

asthma. + chf. + copd. + dementia. + hyper., data = a1, family = "binomial")

anova(modela18, modela19, test = "LRT") #not significant

modela20 <- glm(expb. ~ rc. + hr. + access_4c. + age_rpdb + sex_rpdb. + inc_cat. + edu. + w1. +

dependency_da + deprivation_da + ethniccon_da + instability_da + rurality. + rh_1. +

asthma. + chf. + copd. + dementia. + hyper. + diabetes.,

data = a1, family = "binomial")

anova(modela19, modela20, test = "LRT") #not significant

modela21 <- glm(expb. ~ rc. + hr. + access_4c. + age_rpdb + sex_rpdb. + inc_cat. + edu. + w1. +

dependency_da + deprivation_da + ethniccon_da + instability_da + rurality. + rh_1. +

asthma. + chf. + copd. + dementia. + hyper. + diabetes. + rheum_arth.,

data = a1, family = "binomial")

anova(modela20, modela21, test = "LRT") #not significant

modela22 <- glm(expb. ~ rc. + hr. + access_4c. + age_rpdb + sex_rpdb. + inc_cat. + edu. + w1. +

dependency_da + deprivation_da + ethniccon_da + instability_da + rurality. + rh_1. +

asthma. + chf. + copd. + dementia. + hyper. + diabetes. + rheum_arth. + crohns.,

data = a1, family = "binomial")

anova(modela21, modela22, test = "LRT") #not significant

modela23 <- glm(expb. ~ rc. + hr. + access_4c. + age_rpdb + sex_rpdb. + inc_cat. + edu. + w1. +

dependency_da + deprivation_da + ethniccon_da + instability_da + rurality. + rh_1. +

asthma. + chf. + copd. + dementia. + hyper. + diabetes. + rheum_arth. + crohns. +

ulcerative_colitis., data = a1, family = "binomial")

anova(modela22, modela23, test = "LRT") #not significant

modela24 <- glm(expb. ~ rc. + hr. + access_4c. + age_rpdb + sex_rpdb. + inc_cat. + edu. + w1. +

dependency_da + deprivation_da + ethniccon_da + instability_da + rurality. + rh_1. +

asthma. + chf. + copd. + dementia. + hyper. + diabetes. + rheum_arth. + crohns. +

ulcerative_colitis. + heart_attack., data = a1, family = "binomial")

anova(modela23, modela24, test = "LRT") #not significant

modela25 <- glm(expb. ~ rc. + hr. + access_4c. + age_rpdb + sex_rpdb. + inc_cat. + edu. + w1. +

dependency_da + deprivation_da + ethniccon_da + instability_da + rurality. + rh_1. +

comorb. + heart_attack., data = a1, family = "binomial")

anova(modela24, modela25, test = "LRT") #not significant

#overall examination

tab_model(modela25)

anova(modela24, test = "Chisq")

anova(modela25, test = "Chisq")

vif(modela24)

vif(modela25)

#provider-level predictors

modela26 <- glm(expb. ~ rc. + hr. + access_4c. + age_rpdb + sex_rpdb. + inc_cat. + edu. + w1. +

dependency_da + deprivation_da + ethniccon_da + instability_da + rurality. + rh_1. +

num_encounters_1yr, data = a1, family = "binomial")

anova(modela25, modela26, test = "LRT") #not significant

modela27 <- glm(expb. ~ rc. + hr. + access_4c. + age_rpdb + sex_rpdb. + inc_cat. + edu. + w1. +

dependency_da + deprivation_da + ethniccon_da + instability_da + rurality. + rh_1. +

num_encounters_1yr + practype., data = a1, family = "binomial")

anova(modela26, modela27, test = "LRT") #not significant

modela28 <- glm(expb. ~ rc. + hr. + access_4c. + age_rpdb + sex_rpdb. + inc_cat. + edu. + w1. +

dependency_da + deprivation_da + ethniccon_da + instability_da + rurality. + rh_1. +

num_encounters_1yr + practype. + progtype., data = a1, family = "binomial")

anova(modela27, modela28, test = "LRT") #not significant

modela29 <- glm(expb. ~ rc. + hr. + access_4c. + age_rpdb + sex_rpdb. + inc_cat. + edu. + w1. +

dependency_da + deprivation_da + ethniccon_da + instability_da + rurality. + rh_1. +

num_encounters_1yr + practype. + progtype. + lhin., data = a1, family = "binomial")

anova(modela28, modela29, test = "LRT") #not significant

vif(modela29)

vif(modela28)

anova(modela28, test = "Chisq")

tab_model(modela28)

#test interactions - age, num_encounters, w1 ----

#no interaction - age

#significant interaction - access_4c:num_encounters

#near significant interaction - sex:w1 p = 0.06197

modela30 <- glm(expb. ~ rc. + hr. + access_4c.*num_encounters_1yr + age_rpdb + sex_rpdb. + inc_cat. + edu. +

dependency_da + deprivation_da + ethniccon_da + instability_da + rurality. + rh_1. +

practype. + progtype., data = a1, family = "binomial")

tab_model(modela30)

#stratified analysis - num_encounters_1yr

ggplot(data = a1, aes(x = num_encounters_1yr)) +

geom_bar(stat = "count", width = 0.7) +

geom_text(stat = "count", aes(label = ..count..), vjust = -1) +

ggtitle("Number of encounters (1 year)") +

ylab("Count") +

xlab("Response categories")

a1 = a1 %>% mutate(num_encounters_1yr. = ifelse(num_encounters_1yr <= 3, "<= 3 encounters","> 3 encounters"))

a1$num_encounters_1yr. <- factor(a1$num_encounters_1yr.)

summary(a1$num_encounters_1yr.)

#<= 3 encounters

modela30a <- glm(expb. ~ rc. + hr. + access_4c. + age_rpdb + sex_rpdb. + inc_cat. + edu. +

dependency_da + deprivation_da + ethniccon_da + instability_da + rurality. + rh_1. +

practype. + progtype., data = a1, subset = num_encounters_1yr. == "<= 3 encounters",

family = "binomial")

tab_model(modela30a)

vif(modela30a)

#check and omit outliers

plot(modela30a, which = 5)

cooksd1 <- cooks.distance(modela30a)

plot(cooksd1, pch = "*", cex = 2, main = "Influential observations by Cooks distance")

abline(h = (4/1730), col = "red")

inf30a <- as.numeric(names(cooksd1)[(cooksd1 > (4/1730))])

infdfa1ba <- a1[-inf30a,]

modela30a1 <- glm(expb. ~ rc. + hr. + access_4c. + age_rpdb + sex_rpdb. + inc_cat. + edu. +

dependency_da + deprivation_da + ethniccon_da + instability_da + rurality. + rh_1. +

practype. + progtype., data = infdfa1ba, subset = num_encounters_1yr. == "<= 3 encounters",

family = "binomial")

tab_model(modela30a1)

vif(modela30a1)

#ROC

ldf <- subset(infdfa1ba, num_encounters_1yr. == "<= 3 encounters")

ldfa1 <- subset(a1, num_encounters_1yr. == "<= 3 encounters")

roc(ldf$expb., modela30a1$fitted.values, plot = T,

legacy.axes = T, percent = T, xlab = "False Positive Percentage",

ylab = "True Positive Percentage", col = "blue", lwd = 3, print.auc = T)

#subset characteristics

summary(ldf, subset = expb. == "Poorer experience")

summary(ldf, subset = expb. == "Positive experience")

#> 3 encounters

modela30b <- glm(expb. ~ rc. + hr. + access_4c. + age_rpdb + sex_rpdb. + inc_cat. + edu. +

dependency_da + deprivation_da + ethniccon_da + instability_da + rurality. + rh_1. +

practype. + progtype.,

data = a1, subset = num_encounters_1yr. == "> 3 encounters",

family = "binomial")

tab_model(modela30b)

vif(modela30b)

summary(a1, subset = num_encounters_1yr. == "> 3 encounters")

#check and omit outliers

plot(modela30b, which = 5)

cooksd2 <- cooks.distance(modela30b)

plot(cooksd2, pch = "*", cex = 2, main = "Influential observations by Cooks distance")

abline(h = (4/1102), col = "red")

inf30b <- as.numeric(names(cooksd2)[(cooksd2 > (4/1102))])

infdfa1b <- a1[-c(216,713,825,1508,1592,1753),]

modela30b1 <- glm(expb. ~ rc. + hr. + access_4c. + age_rpdb + sex_rpdb. + inc_cat. + edu. +

dependency_da + deprivation_da + ethniccon_da + instability_da + rurality. + rh_1. +

practype. + progtype., data = infdfa1b, subset = num_encounters_1yr. == "> 3 encounters",

family = "binomial")

tab_model(modela30b1)

#remove program and practice type - problematic

modela30b2 <- glm(expb. ~ rc. + hr. + access_4c. + age_rpdb + sex_rpdb. + inc_cat. + edu. +

dependency_da + deprivation_da + ethniccon_da + instability_da + rurality. + rh_1.,

data = infdfa1b, subset = num_encounters_1yr. == "> 3 encounters",

family = "binomial")

tab_model(modela30b2)

vif(modela30b2)

#ROC

hdf <- subset(infdfa1b, num_encounters_1yr. == "> 3 encounters")

hdfa1 <- subset(a1, num_encounters_1yr. == "> 3 encounters")

roc(hdf$expb., modela30b2$fitted.values, plot = T,

legacy.axes = T, percent = T, xlab = "False Positive Percentage",

ylab = "True Positive Percentage", col = "blue", lwd = 3, print.auc = T)

#subset characteristics

summary(hdf, subset = expb. == "Poorer experience")

summary(ldf, subset = expb. == "Positive experience")

#multivariable logistic regression - expb2. ----

#buidling hiearchal model

#level 1: digital health use

#level 2: patient characteristics (age, sex, income, education)

#level 3: rurality, onmarg

#level 3: patient health conditions

#level 4: healthcare use (satisfaction with healthcare, program type, number of

#encounters in past 1yr, practice type)

#only digital health predictors

model2a3 <- glm(expb2. ~ rc. + hr. + access_4c., data = a1, family = "binomial")

summary(model2a3)

tab_model(model2a3)

vif(model2a3)

#patient characteristics

model2a4 <- glm(expb2. ~ rc. + hr. + access_4c. + age_rpdb,

data = a1, family = "binomial")

anova(model2a3, model2a4, test = "LRT") #significant

model2a5 <- glm(expb2. ~ rc. + hr. + access_4c. + age_rpdb + sex_rpdb.,

data = a1, family = "binomial")

anova(model2a4, model2a5, test = "LRT") #not significant

model2a6 <- glm(expb2. ~ rc. + hr. + access_4c. + age_rpdb + sex_rpdb.,

data = a1, family = "binomial")

anova(model2a4, model2a5, test = "LRT") #not significant

model2a6 <- glm(expb2. ~ rc. + hr. + access_4c. + age_rpdb + sex_rpdb. + inc_cat.,

data = a1, family = "binomial")

anova(model2a5, model2a6, test = "LRT") #significant

model2a7 <- glm(expb2. ~ rc. + hr. + access_4c. + age_rpdb + sex_rpdb. + inc_cat. + fin_sit.,

data = a1, family = "binomial")

anova(model2a6, model2a7, test = "LRT") #significant

model2a8 <- glm(expb2. ~ rc. + hr. + access_4c. + age_rpdb + sex_rpdb. + inc_cat. + fin_sit. + edu.,

data = a1, family = "binomial")

anova(model2a7, model2a8, test = "LRT") #not significant

model2a9 <- glm(expb2. ~ rc. + hr. + access_4c. + age_rpdb + sex_rpdb. + fin_sit. + edu. + w1.,

data = a1, family = "binomial")

anova(model2a8, model2a9, test = "LRT") #not significant

model2a9a <- glm(expb2. ~ rc. + hr. + access_4c. + age_rpdb + sex_rpdb. + fin_sit. + edu. + w1. + lang.,

data = a1, family = "binomial")

anova(model2a9, model2a9a, test = "LRT") #not significant

#overall examination

anova(model2a9a, test = "Chisq")

tab_model(model2a9a)

vif(model2a9a)

#level 2: patient health

model2a10 <- glm(expb2. ~ rc. + hr. + access_4c. + age_rpdb + sex_rpdb. + fin_sit. + edu. + w1. + lang. +

rh_1., data = a1, family = "binomial")

anova(model2a9, model2a10, test = "LRT") #significant

model2a11 <- glm(expb2. ~ rc. + hr. + access_4c. + age_rpdb + sex_rpdb. + fin_sit. + edu. + w1. + lang. +

rh_1. + ADG., data = a1, family = "binomial")

anova(model2a10, model2a11, test = "LRT") #not significant

#overall examination

anova(model2a11, test = "Chisq")

tab_model(model2a11)

vif(model2a11)

#level 4: provider level

model2a12 <- glm(expb2. ~ rc. + hr. + access_4c. + age_rpdb + sex_rpdb. + fin_sit. + edu. + w1. + lang. +

rh_1. + ADG. + num_encounters_1yr, data = a1, family = "binomial")

anova(model2a11, model2a12, test = "LRT") #not significant

model2a13 <- glm(expb2. ~ rc. + hr. + access_4c. + age_rpdb + sex_rpdb. + fin_sit. + edu. + w1. + lang. +

rh_1. + ADG. + num_encounters_1yr + progtype., data = a1, family = "binomial")

anova(model2a12, model2a13, test = "LRT") # significant

model2a14 <- glm(expb2. ~ rc. + hr. + access_4c. + age_rpdb + sex_rpdb. + fin_sit. + edu. + w1. + lang. +

rh_1. + ADG. + num_encounters_1yr + progtype. + practype., data = a1, family = "binomial")

anova(model2a13, model2a14, test = "LRT") #not significant

model2a15 <- glm(expb2. ~ rc. + hr. + access_4c. + age_rpdb + sex_rpdb. + fin_sit. + edu. + w1. + lang. +

rh_1. + ADG. + num_encounters_1yr + progtype. + practype. + fdy., data = a1, family = "binomial")

anova(model2a14, model2a15, test = "LRT") #not significant

#overall examination

anova(model2a15, test = "Chisq")

tab_model(model2a15)

vif(model2a15)

#level 5: geographic predictors

model2a16 <- glm(expb2. ~ rc. + hr. + access_4c. + age_rpdb + sex_rpdb. + fin_sit. + edu. + w1. + lang. +

rh_1. + ADG. + num_encounters_1yr + progtype. + practype. + fdy. + rurality., data = a1, family = "binomial")

anova(model2a15, model2a16, test = "LRT") #not significant

model2a17 <- glm(expb2. ~ rc. + hr. + access_4c. + age_rpdb + sex_rpdb. + fin_sit. + edu. + w1. + lang. +

rh_1. + ADG. + num_encounters_1yr + progtype. + practype. + fdy. + rurality. + lang. +

dependency., data = a1, family = "binomial")

anova(model2a16, model2a17, test = "LRT") #not significant

model2a18 <- glm(expb2. ~ rc. + hr. + access_4c. + age_rpdb + sex_rpdb. + fin_sit. + edu. + w1. + lang. +

rh_1. + ADG. + num_encounters_1yr + progtype. + practype. + fdy. + rurality. +

dependency. + deprivation., data = a1, family = "binomial")

anova(model2a17, model2a18, test = "LRT") #not significant

model2a19 <- glm(expb2. ~ rc. + hr. + access_4c. + age_rpdb + sex_rpdb. + fin_sit. + edu. + w1. + lang. +

rh_1. + ADG. + num_encounters_1yr + progtype. + practype. + fdy. + rurality. +

dependency. + deprivation. + ethnic., data = a1, family = "binomial")

anova(model2a18, model2a19, test = "LRT") #significant

model2a20 <- glm(expb2. ~ rc. + hr. + access_4c. + age_rpdb + sex_rpdb. + fin_sit. + edu. + w1. + lang. +

rh_1. + ADG. + num_encounters_1yr + progtype. + practype. + fdy. + rurality. +

dependency. + deprivation. + ethnic. + instability., data = a1, family = "binomial")

anova(model2a19, model2a20, test = "LRT") #not significant

#overall examination

anova(model2a20, test = "Chisq")

tab_model(model2a20)

vif(model2a20)

#test interactions - age, num_encounters, w1 ----

#no interaction - age

#significant interaction - access_4c:num_encounters

#no significant interaction - w1

model2a33 <- glm(expb2. ~ rc.*age_rpdb + hr. + access_4c.*num_encounters_1yr + age_rpdb + sex_rpdb. + fin_sit. + lang. + edu. + w1. +

dependency_da + deprivation_da + ethniccon_da + instability_da + rurality. + rh_1. + ADG. +

practype. + progtype. + fdy., data = a1, family = "binomial")

summary(model2a33)

tab_model(model2a30)

model2a34 <- glm(expb2. ~ rc. + hr. + access_4c. + age_rpdb + sex_rpdb. + fin_sit. + lang. + edu. + w1. +

dependency_da + deprivation_da + ethniccon_da + instability_da + rurality. + rh_1. + ADG. +

practype. + progtype. + fdy., data = a1, family = "binomial")

tab_model(model2a34)

vif(model2a34)

#test inc_cat vs fin_sit

at1 <- glm(expb2. ~ inc_cat., data = a1, family = "binomial")

at2 <- glm(expb2. ~ fin_sit., data = a1, family = "binomial") #reduction in deviance

anova(at1, at2)

#unstratified

model2a35 <- glm(expb2. ~ rc. + hr. + access_4c. + agec. + sex_rpdb. + fin_sit. + edu. + lang. +

rh_1. + ADG. + progtype. + fdy. + rurality. +

dependency. + deprivation. + ethnic. + instability., data = a1, family = "binomial")

tab_model(model2a35)

vif(model2a35)

#check and omit outliers

plot(model2a35, which = 5)

cooks2d0 <- cooks.distance(model2a35)

plot(cooks2d0, pch = "*", cex = 2, main = "Influential observations by Cooks distance")

abline(h = (4/2692), col = "red")

in2f30a. <- as.numeric(names(cooks2d0)[(cooks2d0 > (4/2692))])

infdf2a1b0 <- a1[-in2f30a.,]

model2a30a0 <- glm(expb2. ~ rc. + hr. + access_4c. + agec. + sex_rpdb. + fin_sit. + edu. + lang. +

rh_1. + ADG. + progtype. + fdy. + rurality. +

dependency. + deprivation. + ethnic. + instability., data = infdf2a1b0, family = "binomial")

tab_model(model2a30a0)

vif(model2a30a0)

#ROC

roc(infdf2a1b0$expb2., model2a30a0$fitted.values, plot = T,

legacy.axes = T, percent = T, xlab = "False Positive Percentage",

ylab = "True Positive Percentage", col = "blue", lwd = 3, print.auc = T)

#unadjusted odds ratios

tab_model(glm(expb2. ~ rc., data = infdf2a1b0, family = "binomial"))

tab_model(glm(expb2. ~ hr., data = infdf2a1b0, family = "binomial"))

tab_model(glm(expb2. ~ access_4c., data = infdf2a1b0, family = "binomial"))

tab_model(glm(expb2. ~ agec., data = infdf2a1b0, family = "binomial"))

tab_model(glm(expb2. ~ sex_rpdb., data = infdf2a1b0, family = "binomial"))

tab_model(glm(expb2. ~ fin_sit., data = infdf2a1b0, family = "binomial"))

tab_model(glm(expb2. ~ edu., data = infdf2a1b0, family = "binomial"))

tab_model(glm(expb2. ~ lang., data = infdf2a1b0, family = "binomial"))

tab_model(glm(expb2. ~ rh_1., data = infdf2a1b0, family = "binomial"))

tab_model(glm(expb2. ~ ADG., data = infdf2a1b0, family = "binomial"))

tab_model(glm(expb2. ~ progtype., data = infdf2a1b0, family = "binomial"))

tab_model(glm(expb2. ~ fdy., data = infdf2a1b0, family = "binomial"))

tab_model(glm(expb2. ~ rurality., data = infdf2a1b0, family = "binomial"))

tab_model(glm(expb2. ~ dependency., data = infdf2a1b0, family = "binomial"))

tab_model(glm(expb2. ~ deprivation., data = infdf2a1b0, family = "binomial"))

tab_model(glm(expb2. ~ ethnic., data = infdf2a1b0, family = "binomial"))

tab_model(glm(expb2. ~ instability., data = infdf2a1b0, family = "binomial"))

#<= 3 encounters

model2a35a <- glm(expb2. ~ rc. + hr. + access_4c. + agec. + sex_rpdb. + fin_sit. + edu. + lang. +

rh_1. + ADG. + progtype. + fdy. + rurality. +

dependency. + deprivation. + ethnic. + instability., data = a1, subset = num_encounters_1yr. == "<= 3 encounters",

family = "binomial")

tab_model(model2a35a)

vif(model2a35a)

#check and omit outliers

plot(model2a35a, which = 5)

cooks2d1 <- cooks.distance(model2a35a)

plot(cooks2d1, pch = "*", cex = 2, main = "Influential observations by Cooks distance")

abline(h = (4/1651), col = "red")

in2f30a <- as.numeric(names(cooks2d1)[(cooks2d1 > (4/1651))])

infdf2a1ba <- a1[-in2f30a,]

model2a30a1 <- glm(expb2. ~ rc. + hr. + access_4c. + agec. + sex_rpdb. + fin_sit. + edu. + lang. +

rh_1. + ADG. + progtype. + fdy. + rurality. +

dependency. + deprivation. + ethnic. + instability., data = infdf2a1ba, subset = num_encounters_1yr. == "<= 3 encounters",

family = "binomial")

tab_model(model2a30a1)

vif(model2a30a1)

#ROC

ldf <- subset(infdf2a1ba, num_encounters_1yr. == "<= 3 encounters")

ldfa1 <- subset(a1, num_encounters_1yr. == "<= 3 encounters")

roc(ldf$expb2., model2a30a1$fitted.values, plot = T,

legacy.axes = T, percent = T, xlab = "False Positive Percentage",

ylab = "True Positive Percentage", col = "blue", lwd = 3, print.auc = T)

#subset characteristics

summary(ldf, subset = expb. == "Poorer experience")

summary(ldf, subset = expb. == "Positive experience")

#> 3 encounters

modela35b <- glm(expb2. ~ rc. + hr. + access_4c. + agec. + sex_rpdb. + fin_sit. + edu. + lang. +

rh_1. + ADG. + progtype. + fdy. + rurality. +

dependency. + deprivation. + ethnic. + instability.,

data = a1, subset = num_encounters_1yr. == "> 3 encounters",

family = "binomial")

tab_model(modela35b)

vif(modela35b)

summary(a1, subset = num_encounters_1yr. == "> 3 encounters")

#check and omit outliers

plot(modela35b, which = 5)

cooksd2 <- cooks.distance(modela35b)

plot(cooksd2, pch = "*", cex = 2, main = "Influential observations by Cooks distance")

abline(h = (4/1041), col = "red")

inf30b <- as.numeric(names(cooksd2)[(cooksd2 > (4/1041))])

infdfa1b <- a1[-inf30b,]

modela35b1 <- glm(expb2. ~ rc. + hr. + access_4c. + agec. + sex_rpdb. + fin_sit. + edu. + lang. +

rh_1. + ADG. + progtype. + fdy. + rurality. +

dependency. + deprivation. + ethnic. + instability., data = infdfa1b, subset = num_encounters_1yr. == "> 3 encounters",

family = "binomial")

tab_model(modela35b1)

vif(modela35b1)

#ROC

hdf <- subset(infdfa1b, num_encounters_1yr. == "> 3 encounters")

hdfa1 <- subset(a1, num_encounters_1yr. == "> 3 encounters")

roc(hdf$expb2., modela35b1$fitted.values, plot = T,

legacy.axes = T, percent = T, xlab = "False Positive Percentage",

ylab = "True Positive Percentage", col = "blue", lwd = 3, print.auc = T)

#subset characteristics

summary(hdf, subset = expb. == "Poorer experience")

summary(ldf, subset = expb. == "Positive experience")

#forest plots ----

#digital health and patient characteristics

fpa1a <- plot_models(model2a30a0, model2a30a1, modela35b1,

show.values = F, show.p = T, spacing = .7, value.size = 4,

vline.color = "grey" , legend.title = "Models",

m.labels = c("Unstratified","\u2264 3 encounters", "> 3 encounters"),

axis.labels = c("Non-English speaking at home","Some college/undergraduate","Postgraduate/professional degree","College/undergraduate degree",

"Tight/Very tight/Poor financial situation","Comfortable financial situation","Male","\u2265 65 years",

"45-64 years","Online booking access","Digital health record access","Telehealth access"),

rm.terms = c("fdy. [4-9,10-19,20 or more]","ADG. [> 9, 3-4,5-6,7-8]","rh_1. [Fair,Good,Very good,Excellent]",

"practype. [ccpc phys]", "progtype. [Capitation, Other]","rurality. [Rural,Small urban,Urban]",

"dependency. [2,3,4,5]","deprivation. [2,3,4,5]","ethnic. [2,3,4,5]","instability. [2,3,4,5]")) +

theme_bw()

fpa1b <- plot_models(model2a30a0, model2a30a1, modela35b1,

show.values = F, show.p = T, spacing = .7, value.size = 4,

vline.color = "grey" , legend.title = "Models",

m.labels = c("Unstratified","\u2264 3 encounters", "> 3 encounters"),

axis.labels = c("4-9 years with provider","\u2265 20 years with provider","10-19 years with provider","Capitation (FHN/FHO)",

"Other payment model","ADG 7-8","ADG 5-6", "ADG 3-4","ADG > 9","Excellent health","Very good health","Good health","Fair health"),

rm.terms = c("rc. [Remote communication]","hr. [Health record]", "access_4c. [Yes]",

"agec. [45-64,65+]","sex_rpdb. [M]","fin_sit. [Comfortable,Tight/Very tight/Poor]",

"edu. [College/undergraduate degree,Postgraduate/professional degree,Some college/undergraduate]",

"lang. [Other]","rurality. [Rural,Small urban,Urban]",

"dependency. [2,3,4,5]","deprivation. [2,3,4,5]","ethnic. [2,3,4,5]","instability. [2,3,4,5]")) +

theme_bw()

fpa1c <- plot_models(model2a30a0, model2a30a1, modela35b1,

show.values = F, show.p = T, spacing = .7, value.size = 4,

vline.color = "grey" , legend.title = "Models",

m.labels = c("Unstratified","\u2264 3 encounters", "> 3 encounters"),

axis.labels = c("Residential instability (5th quintile)","Residential instability (4th quintile)","Residential instability (3rd quintile)",

"Residential instability (2nd quintile)","Ethnic concentration (5th quintile)","Ethnic concentration (4th quintile)",

"Ethnic concentration (3rd quintile)","Ethnic concentration (2nd quintile)","Material deprivation (5th quintile)","Material deprivation (4th quintile)",

"Material deprivation (3rd quintile)","Material deprivation (2nd quintile)","Dependency (5th quintile)","Dependency (4th quintile)",

"Dependency (3rd quintile)", "Dependency (2nd quintile)","Urban area","Small urban area","Rural area"),

rm.terms = c("fdy. [4-9,10-19,20 or more]","ADG. [> 9, 3-4,5-6,7-8]","rh_1. [Fair,Good,Very good,Excellent]",

"practype. [ccpc phys]", "progtype. [Capitation, Other]","rc. [Remote communication]","hr. [Health record]", "access_4c. [Yes]",

"agec. [45-64,65+]","sex_rpdb. [M]","fin_sit. [Comfortable,Tight/Very tight/Poor]",

"edu. [College/undergraduate degree,Postgraduate/professional degree,Some college/undergraduate]",

"lang. [Other]")) +

theme_bw()

modela4 <- glm(expb. ~ rc. + hr. + access_4c. + age_rpdb + sex_rpdb. + inc_cat. +

w1. + edu. + dependency. + deprivation. + ethnic. + instability.,

data = a1, family = "binomial")

summary(modela4)

tab_model(modela4)

vif(modela4)

anova(modela3, modela4, test = "LRT")

#patient health conditions

modela5 <- glm(expb. ~ rc. + hr. + access_4c. + age_rpdb + sex_rpdb. + inc_cat. +

w1. + edu. + dependency. + deprivation. + ethnic. + instability. +

asthma. + chf. + copd. + dementia. + hyper. +

diabetes. + rheum_arth. + crohns. + ulcerative_colitis. + heart_attack. + rh_1.,

data = a1, family = "binomial")

summary(modela5)

tab_model(modela5)

vif(modela5)

anova(modela4, modela5, test = "Chisq") #no significant improvement in model

#alternative: only use number of chronic conditions

modela6 <- glm(expb. ~ rc. + hr. + access_4c. + age_rpdb + sex_rpdb. + inc_cat. +

w1. + edu. + dependency. + deprivation. + ethnic. + instability. +

comorb. + heart_attack. + rh_1., data = a1, family = "binomial")

summary(modela6)

tab_model(modela6)

vif(modela6)

anova(modela4, modela6, test = "Chisq")

#healthcare utilization

modela7 <- glm(expb. ~ rc. + hr. + access_4c. + age_rpdb + sex_rpdb. + inc_cat. +

w1. + edu. + dependency. + deprivation. + ethnic. + instability. +

progtype. + practype. + w1. + lhin. + comorb. + heart_attack. + rh_1.,

data = a1, family = "binomial")

summary(modela7)

tab_model(modela7)

vif(modela7)

anova(modela6, modela7, test = "Chisq")

#####

#multivariable logistic regression ----

#only digital health predictors

modela3 <- glm(expb. ~ rc. + hr. + access_4c., data = a1, family = "binomial")

summary(modela3)

tab_model(modela3)

vif(modela3)

#patient demographics

#no interaction terms: age + sex

modela4 <- glm(expb. ~ rc. + hr. + access_4c. + age_rpdb + sex_rpdb. + inc_cat. +

w1. + edu. + rurality., data = a1, family = "binomial")

summary(modela4)

tab_model(modela4)

vif(modela4)

anova(modela3, modela4, test = "Chisq")

#ROC

roc(a1$expb., modela4$fitted.values, plot = T,

legacy.axes = T, percent = T, xlab = "False Positive Percentage",

ylab = "True Positive Percentage", col = "blue", lwd = 3, print.auc = T)

#health care utilization

modela5 <- glm(expb. ~ rc. + hr. + access_4c. + age_rpdb + sex_rpdb. + inc_cat. +

w1. + edu. + num_encounters_1yr + lhin. + progtype. + practype. +

rurality., data = a1, family = "binomial")

summary(modela5)

tab_model(modela5)

vif(modela5)

anova(modela4, modela5, test = "Chisq")

#health care utilization and conditions

#remove comorb.; high vif

#potentially remove rurality; moderate vif (maybe influencing ethnic. and instability.)

modela6 <- glm(expb. ~ rc. + hr. + access_4c. + age_rpdb + sex_rpdb. + inc_cat. +

w1. + edu. + num_encounters_1yr + lhin. + progtype. + practype. +

rurality. + asthma. + chf. + copd. + dementia. + hiv. + hyper. +

diabetes. + rheum_arth. + crohns. + ulcerative_colitis. + heart_attack. +

rurality. + deprivation. + dependency. + ethnic. + instability.,

data = a1, family = "binomial")

summary(modela6)

tab_model(modela6)

vif(modela6)

anova(modela6, modela5, test = "Chisq")

#Stepwise regression using AIC

step.modela <- stepAIC(modela6, direction = "backward", trace = F)

summary(step.modela)

tab_model(step.modela)

vif(step.modela)

#ROC

roc(a1$expb., step.modela$fitted.values, plot = T,

legacy.axes = T, percent = T, xlab = "False Positive Percentage",

ylab = "True Positive Percentage", col = "blue", lwd = 3, print.auc = T)

##A4 ANALYSIS## ----

summary(factor(a4$exp_recode_1))

a4 = a4 %>% mutate(exp1. = ifelse(exp_recode_1 == 4 | exp_recode_1 == 5, "Always/Often","Sometimes/Rarely/Never"))

a4$exp1. <- factor(a4$exp1.)

summary(a4$exp1.)

summary(factor(a4$exp_recode_2))

a4 = a4 %>% mutate(exp2. = ifelse(exp_recode_2 == 4 | exp_recode_2 == 5, "Always/Often","Sometimes/Rarely/Never"))

a4$exp2. <- factor(a4$exp2.)

summary(a4$exp2.)

a4 = a4 %>% mutate(exp3. = ifelse(exp_recode_3 == 4 | exp_recode_3 == 5, "Always/Often","Sometimes/Rarely/Never"))

a4$exp3. <- factor(a4$exp3.)

summary(a4$exp3.)

a4 = a4 %>% mutate(exp4. = ifelse(exp_recode_4 == 4 | exp_recode_4 == 5, "Always/Often","Sometimes/Rarely/Never"))

a4$exp4. <- factor(a4$exp4.)

summary(a4$exp4.)

a4 = a4 %>% mutate(exp5. = ifelse(exp_recode_5 == 4 | exp_recode_5 == 5, "Always/Often","Sometimes/Rarely/Never"))

a4$exp5. <- factor(a4$exp5.)

summary(a4$exp5.)

#exp1 ----

#significant association in age and rural (0.031)

#signficant association between number of encounters and ADG

model4a00 <- glm(exp1. ~ rc. + hr. + access_4c. + sex_rpdb. + fin_sit. + edu. + lang. +

rh_1. + ADG.*num_encounters_1yr + progtype. + fdy. + rurality.*age_rpdb +

dependency. + deprivation. + ethnic. + instability., data = a4, family = "binomial")

summary(model4a00)

#create categories

a4 = a4 %>% mutate(ages. = ifelse(age_rpdb <= 60, "<= 60 years","> 60 years"))

a4$ages. <- factor(a4$ages.)

summary(a4$ages.)

a4 = a4 %>% mutate(num_encounters_1yr. = ifelse(num_encounters_1yr <= 3, "<= 3 encounters","> 3 encounters"))

a4$num_encounters_1yr. <- factor(a4$num_encounters_1yr.)

summary(a4$num_encounters_1yr.)

#full model

model4a1 <- glm(exp1. ~ rc. + hr. + access_4c. + sex_rpdb. + fin_sit. + edu. + lang. +

rh_1. + ADG. + progtype. + fdy. + rurality. +

dependency. + deprivation. + ethnic. + instability., data = a4, family = "binomial")

vif(model4a1)

anova(model4a1, test = "Chisq")

tab_model(model4a1)

#remove inf. outliers

plot(model4a1, which = 5)

cooks4d0 <- cooks.distance(model4a1)

plot(cooks4d0, pch = "*", cex = 2, main = "Influential observations by Cooks distance")

abline(h = (4/2692), col = "red")

inf4a. <- as.numeric(names(cooks4d0)[(cooks4d0 > (4/2692))])

head(inf4a.)

infdf5a <- a4[-inf4a.,]

model4a1. <- glm(exp1. ~ rc. + hr. + access_4c. + sex_rpdb. + fin_sit. + edu. + lang. +

rh_1. + ADG. + progtype. + fdy. + rurality. +

dependency. + deprivation. + ethnic. + instability., data = infdf5a, family = "binomial")

tab_model(model4a1.)

vif(model4a1.)

#ROC

roc(infdf5a$exp1., model4a1.$fitted.values, plot = T,

legacy.axes = T, percent = T, xlab = "False Positive Percentage",

ylab = "True Positive Percentage", col = "blue", lwd = 3, print.auc = T)

#< 60 years

model4a2 <- glm(exp1. ~ rc. + hr. + access_4c. + sex_rpdb. + fin_sit. + edu. + lang. +

rh_1. + ADG. + progtype. + fdy. + rurality. +

dependency. + deprivation. + ethnic. + instability., data = a4,

subset = ages. == "<= 60 years", family = "binomial")

vif(model4a2)

tab_model(model4a2)

#remove inf. outliers

plot(model4a2, which = 5)

cooks4d02 <- cooks.distance(model4a2)

plot(cooks4d02, pch = "*", cex = 2, main = "Influential observations by Cooks distance")

abline(h = (4/1381), col = "red")

inf4b. <- as.numeric(names(cooks4d02)[(cooks4d02 > (4/1381))])

head(inf4b.)

infdf4b <- a4[-inf4b.,]

model4a2. <- glm(exp1. ~ rc. + hr. + access_4c. + sex_rpdb. + fin_sit. + edu. + lang. +

rh_1. + ADG. + progtype. + fdy. + rurality. +

dependency. + deprivation. + ethnic. + instability., data = infdf4b, subset =

ages. == "<= 60 years", family = "binomial")

tab_model(model4a2.)

vif(model4a2.)

#ROC

ro1 <- subset(infdf4b, ages. == "<= 60 years")

roc(ro1$exp1., model4a2.$fitted.values, plot = T,

legacy.axes = T, percent = T, xlab = "False Positive Percentage",

ylab = "True Positive Percentage", col = "blue", lwd = 3, print.auc = T)

#> 60 years

model4a3 <- glm(exp1. ~ rc. + hr. + access_4c. + sex_rpdb. + fin_sit. + edu. + lang. +

rh_1. + ADG. + progtype. + fdy. + rurality. +

dependency. + deprivation. + ethnic. + instability., data = a4,

subset = ages. == "> 60 years", family = "binomial")

vif(model4a3)

tab_model(model4a3)

#remove inf. outliers

plot(model4a3, which = 5)

cooks4d03 <- cooks.distance(model4a3)

plot(cooks4d03, pch = "*", cex = 2, main = "Influential observations by Cooks distance")

abline(h = (4/1311), col = "red")

inf4c. <- as.numeric(names(cooks4d03)[(cooks4d03 > (4/1311))])

head(inf4c.)

infdf4c <- a4[-inf4c.,]

model4a3. <- glm(exp1. ~ rc. + hr. + access_4c. + sex_rpdb. + fin_sit. + edu. + lang. +

rh_1. + ADG. + progtype. + fdy. + rurality. +

dependency. + deprivation. + ethnic. + instability., data = infdf4c, subset =

ages. == "> 60 years", family = "binomial")

tab_model(model4a3.)

vif(model4a3.)

#ROC

ro2 <- subset(infdf4c, ages. == "> 60 years")

roc(ro2$exp1., model4a3.$fitted.values, plot = T,

legacy.axes = T, percent = T, xlab = "False Positive Percentage",

ylab = "True Positive Percentage", col = "blue", lwd = 3, print.auc = T)

#<= 3 encounters

model4a4 <- glm(exp1. ~ rc. + hr. + access_4c. + sex_rpdb. + fin_sit. + edu. + lang. +

rh_1. + ADG. + progtype. + fdy. + rurality. +

dependency. + deprivation. + ethnic. + instability., data = a4,

subset = num_encounters_1yr. == "<= 3 encounters", family = "binomial")

vif(model4a4)

tab_model(model4a4)

#remove inf. outliers

plot(model4a4, which = 5)

cooks4d04 <- cooks.distance(model4a4)

plot(cooks4d04, pch = "*", cex = 2, main = "Influential observations by Cooks distance")

abline(h = (4/1651), col = "red")

inf4d. <- as.numeric(names(cooks4d04)[(cooks4d04 > (4/1651))])

head(inf4d.)

infdf4d <- a4[-inf4c.,]

model4a4. <- glm(exp1. ~ rc. + hr. + access_4c. + sex_rpdb. + fin_sit. + edu. + lang. +

rh_1. + ADG. + progtype. + fdy. + rurality. +

dependency. + deprivation. + ethnic. + instability., data = infdf4d, subset =

num_encounters_1yr. == "<= 3 encounters", family = "binomial")

tab_model(model4a4.)

vif(model4a4.)

#ROC

ro3 <- subset(infdf4d, num_encounters_1yr. == "<= 3 encounters")

roc(ro3$exp1., model4a4.$fitted.values, plot = T,

legacy.axes = T, percent = T, xlab = "False Positive Percentage",

ylab = "True Positive Percentage", col = "blue", lwd = 3, print.auc = T)

#> 3 encounters

model4a5 <- glm(exp1. ~ rc. + hr. + access_4c. + sex_rpdb. + fin_sit. + edu. + lang. +

rh_1. + ADG. + progtype. + fdy. + rurality. +

dependency. + deprivation. + ethnic. + instability., data = a4,

subset = num_encounters_1yr. == "> 3 encounters", family = "binomial")

vif(model4a5)

tab_model(model4a5)

#remove inf. outliers

plot(model4a5, which = 5)

cooks4d05 <- cooks.distance(model4a5)

plot(cooks4d05, pch = "*", cex = 2, main = "Influential observations by Cooks distance")

abline(h = (4/1041), col = "red")

inf4e. <- as.numeric(names(cooks4d05)[(cooks4d05 > (4/1041))])

head(inf4d.)

infdf4e <- a4[-inf4e.,]

model4a5. <- glm(exp1. ~ rc. + hr. + access_4c. + sex_rpdb. + fin_sit. + edu. + lang. +

rh_1. + ADG. + progtype. + fdy. + rurality. +

dependency. + deprivation. + ethnic. + instability., data = infdf4e, subset =

num_encounters_1yr. == "> 3 encounters", family = "binomial")

tab_model(model4a5.)

vif(model4a5.)

#ROC

ro4 <- subset(infdf4e, num_encounters_1yr. == "> 3 encounters")

roc(ro4$exp1., model4a5.$fitted.values, plot = T,

legacy.axes = T, percent = T, xlab = "False Positive Percentage",

ylab = "True Positive Percentage", col = "blue", lwd = 3, print.auc = T)

#exp2 ----

#significant association in age and rural (0.019)

#signficant association between number of encounters and ADG

model4a06 <- glm(exp2. ~ rc.*age_rpdb + hr. + access_4c. + sex_rpdb. + fin_sit. + edu. + lang. +

rh_1. + ADG.*num_encounters_1yr + progtype. + fdy. + rurality. +

dependency. + deprivation. + ethnic. + instability., data = a4, family = "binomial")

summary(model4a06)

#full model

model4a6 <- glm(exp2. ~ rc. + hr. + access_4c. + sex_rpdb. + fin_sit. + edu. + lang. +

rh_1. + ADG. + progtype. + fdy. + rurality. +

dependency. + deprivation. + ethnic. + instability., data = a4, family = "binomial")

anova(model4a6, test = "Chisq")

tab_model(model4a6)

#remove inf. outliers

plot(model4a6, which = 5)

cooks4d02 <- cooks.distance(model4a6)

plot(cooks4d02, pch = "*", cex = 2, main = "Influential observations by Cooks distance")

abline(h = (4/2692), col = "red")

inf4f. <- as.numeric(names(cooks4d02)[(cooks4d02 > (4/2692))])

head(inf4f.)

infdf5f <- a4[-inf4f.,]

model4a6. <- glm(exp2. ~ rc. + hr. + access_4c. + sex_rpdb. + fin_sit. + edu. + lang. +

rh_1. + ADG. + progtype. + fdy. + rurality. +

dependency. + deprivation. + ethnic. + instability., data = infdf5f, family = "binomial")

tab_model(model4a6.)

vif(model4a6.)

#ROC

roc(infdf5f$exp2., model4a6.$fitted.values, plot = T,

legacy.axes = T, percent = T, xlab = "False Positive Percentage",

ylab = "True Positive Percentage", col = "blue", lwd = 3, print.auc = T)

#< 60 years

model4a7 <- glm(exp2. ~ rc. + hr. + access_4c. + sex_rpdb. + fin_sit. + edu. + lang. +

rh_1. + ADG. + progtype. + fdy. + rurality. +

dependency. + deprivation. + ethnic. + instability., data = a4,

subset = ages. == "<= 60 years", family = "binomial")

vif(model4a7)

tab_model(model4a7)

#remove inf. outliers

plot(model4a7, which = 5)

cooks4d02 <- cooks.distance(model4a7)

plot(cooks4d02, pch = "*", cex = 2, main = "Influential observations by Cooks distance")

abline(h = (4/1381), col = "red")

inf4g. <- as.numeric(names(cooks4d02)[(cooks4d02 > (4/1381))])

head(inf4g.)

infdf4g <- a4[-inf4g.,]

model4a7. <- glm(exp2. ~ rc. + hr. + access_4c. + sex_rpdb. + fin_sit. + edu. + lang. +

rh_1. + ADG. + progtype. + fdy. + rurality. +

dependency. + deprivation. + ethnic. + instability., data = infdf4g, subset =

ages. == "<= 60 years", family = "binomial")

tab_model(model4a7.)

vif(model4a7.)

#ROC

ro5 <- subset(infdf4g, ages. == "<= 60 years")

roc(ro5$exp2., model4a7.$fitted.values, plot = T,

legacy.axes = T, percent = T, xlab = "False Positive Percentage",

ylab = "True Positive Percentage", col = "blue", lwd = 3, print.auc = T)

#> 60 years

model4a8 <- glm(exp2. ~ rc. + hr. + access_4c. + sex_rpdb. + fin_sit. + edu. + lang. +

rh_1. + ADG. + progtype. + fdy. + rurality. +

dependency. + deprivation. + ethnic. + instability., data = a4,

subset = ages. == "> 60 years", family = "binomial")

vif(model4a4)

tab_model(model4a8)

#remove inf. outliers

plot(model4a8, which = 5)

cooks4d03 <- cooks.distance(model4a8)

plot(cooks4d03, pch = "*", cex = 2, main = "Influential observations by Cooks distance")

abline(h = (4/1311), col = "red")

inf4h. <- as.numeric(names(cooks4d03)[(cooks4d03 > (4/1311))])

head(inf4h.)

infdf4h <- a4[-inf4h.,]

model4a8. <- glm(exp2. ~ rc. + hr. + access_4c. + sex_rpdb. + fin_sit. + edu. + lang. +

rh_1. + ADG. + progtype. + fdy. + rurality. +

dependency. + deprivation. + ethnic. + instability., data = infdf4h, subset =

ages. == "> 60 years", family = "binomial")

tab_model(model4a8.)

vif(model4a8.)

#ROC

ro6 <- subset(infdf4h, ages. == "> 60 years")

roc(ro6$exp2., model4a8.$fitted.values, plot = T,

legacy.axes = T, percent = T, xlab = "False Positive Percentage",

ylab = "True Positive Percentage", col = "blue", lwd = 3, print.auc = T)

#<= 3 encounters

model4a9 <- glm(exp2. ~ rc. + hr. + access_4c. + sex_rpdb. + fin_sit. + edu. + lang. +

rh_1. + ADG. + progtype. + fdy. + rurality. +

dependency. + deprivation. + ethnic. + instability., data = a4,

subset = num_encounters_1yr. == "<= 3 encounters", family = "binomial")

vif(model4a9)

tab_model(model4a9)

#remove inf. outliers

plot(model4a9, which = 5)

cooks4d06 <- cooks.distance(model4a9)

plot(cooks4d06, pch = "*", cex = 2, main = "Influential observations by Cooks distance")

abline(h = (4/1651), col = "red")

inf4i <- as.numeric(names(cooks4d06)[(cooks4d06 > (4/1651))])

head(inf4i)

infdf4i <- a4[-inf4i,]

model4a9. <- glm(exp2. ~ rc. + hr. + access_4c. + sex_rpdb. + fin_sit. + edu. + lang. +

rh_1. + ADG. + progtype. + fdy. + rurality. +

dependency. + deprivation. + ethnic. + instability., data = infdf4i, subset =

num_encounters_1yr. == "<= 3 encounters", family = "binomial")

tab_model(model4a9.)

vif(model4a9.)

#ROC

ro7 <- subset(infdf4i, num_encounters_1yr. == "<= 3 encounters")

roc(ro7$exp2., model4a9.$fitted.values, plot = T,

legacy.axes = T, percent = T, xlab = "False Positive Percentage",

ylab = "True Positive Percentage", col = "blue", lwd = 3, print.auc = T)

#> 3 encounters

model4a10 <- glm(exp2. ~ rc. + hr. + access_4c. + sex_rpdb. + fin_sit. + edu. + lang. +

rh_1. + ADG. + progtype. + fdy. + rurality. +

dependency. + deprivation. + ethnic. + instability., data = a4,

subset = num_encounters_1yr. == "> 3 encounters", family = "binomial")

vif(model4a10)

tab_model(model4a10)

#remove inf. outliers

plot(model4a10, which = 5)

cooks4d07 <- cooks.distance(model4a10)

plot(cooks4d07, pch = "*", cex = 2, main = "Influential observations by Cooks distance")

abline(h = (4/1041), col = "red")

inf4j. <- as.numeric(names(cooks4d07)[(cooks4d07 > (4/1041))])

head(inf4j.)

infdf4j <- a4[-inf4j.,]

model4a10. <- glm(exp2. ~ rc. + hr. + access_4c. + sex_rpdb. + fin_sit. + edu. + lang. +

rh_1. + ADG. + progtype. + fdy. + rurality. +

dependency. + deprivation. + ethnic. + instability., data = infdf4j, subset =

num_encounters_1yr. == "> 3 encounters", family = "binomial")

tab_model(model4a10.)

vif(model4a10.)

#ROC

ro8 <- subset(infdf4j, num_encounters_1yr. == "> 3 encounters")

roc(ro8$exp2., model4a10.$fitted.values, plot = T,

legacy.axes = T, percent = T, xlab = "False Positive Percentage",

ylab = "True Positive Percentage", col = "blue", lwd = 3, print.auc = T)

#exp3 ----

#significant association in age and rural (0.047)

#signficant association between number of encounters and dependency

model4a011 <- glm(exp3. ~ rc.*age_rpdb + hr. + access_4c. + sex_rpdb. + fin_sit. + edu. + lang. +

rh_1. + ADG. + progtype. + fdy. + rurality. +

dependency.*num_encounters_1yr + deprivation. + ethnic. + instability., data = a4, family = "binomial")

summary(model4a011)

#full model

model4a11 <- glm(exp3. ~ rc. + hr. + access_4c. + sex_rpdb. + fin_sit. + edu. + lang. +

rh_1. + ADG. + progtype. + fdy. + rurality. +

dependency. + deprivation. + ethnic. + instability., data = a4, family = "binomial")

anova(model4a11, test = "Chisq")

tab_model(model4a11)

#remove inf. outliers

plot(model4a11, which = 5)

cooks4d011 <- cooks.distance(model4a11)

plot(cooks4d011, pch = "*", cex = 2, main = "Influential observations by Cooks distance")

abline(h = (4/2692), col = "red")

inf4k. <- as.numeric(names(cooks4d011)[(cooks4d011 > (4/2692))])

head(inf4k.)

infdf5k <- a4[-inf4k.,]

model4a11. <- glm(exp3. ~ rc. + hr. + access_4c. + sex_rpdb. + fin_sit. + edu. + lang. +

rh_1. + ADG. + progtype. + fdy. + rurality. +

dependency. + deprivation. + ethnic. + instability., data = infdf5k, family = "binomial")

tab_model(model4a11.)

vif(model4a11.)

#ROC

roc(infdf5k$exp2., model4a11.$fitted.values, plot = T,

legacy.axes = T, percent = T, xlab = "False Positive Percentage",

ylab = "True Positive Percentage", col = "blue", lwd = 3, print.auc = T)

#< 60 years

model4a12 <- glm(exp3. ~ rc. + hr. + access_4c. + sex_rpdb. + fin_sit. + edu. + lang. +

rh_1. + ADG. + progtype. + fdy. + rurality. +

dependency. + deprivation. + ethnic. + instability., data = a4,

subset = ages. == "<= 60 years", family = "binomial")

vif(model4a12)

tab_model(model4a12)

#remove inf. outliers

plot(model4a12, which = 5)

cooks4d012 <- cooks.distance(model4a12)

plot(cooks4d012, pch = "*", cex = 2, main = "Influential observations by Cooks distance")

abline(h = (4/1381), col = "red")

inf4l. <- as.numeric(names(cooks4d012)[(cooks4d012 > (4/1381))])

head(inf4l.)

infdf4l. <- a4[-inf4l.,]

model4a12. <- glm(exp3. ~ rc. + hr. + access_4c. + sex_rpdb. + fin_sit. + edu. + lang. +

rh_1. + ADG. + progtype. + fdy. + rurality. +

dependency. + deprivation. + ethnic. + instability., data = infdf4l., subset =

ages. == "<= 60 years", family = "binomial")

tab_model(model4a12.)

vif(model4a12.)

#ROC

ro9 <- subset(infdf4l., ages. == "<= 60 years")

roc(ro9$exp2., model4a12.$fitted.values, plot = T,

legacy.axes = T, percent = T, xlab = "False Positive Percentage",

ylab = "True Positive Percentage", col = "blue", lwd = 3, print.auc = T)

#> 60 years

model4a13 <- glm(exp3. ~ rc. + hr. + access_4c. + sex_rpdb. + fin_sit. + edu. + lang. +

rh_1. + ADG. + progtype. + fdy. + rurality. +

dependency. + deprivation. + ethnic. + instability., data = a4,

subset = ages. == "> 60 years", family = "binomial")

vif(model4a13)

tab_model(model4a13)

#remove inf. outliers

plot(model4a13, which = 5)

cooks4d013 <- cooks.distance(model4a13)

plot(cooks4d03, pch = "*", cex = 2, main = "Influential observations by Cooks distance")

abline(h = (4/1311), col = "red")

inf4m. <- as.numeric(names(cooks4d013)[(cooks4d013 > (4/1311))])

head(inf4m.)

infdf4m <- a4[-inf4m.,]

model4a13. <- glm(exp3. ~ rc. + hr. + access_4c. + sex_rpdb. + fin_sit. + edu. + lang. +

rh_1. + ADG. + progtype. + fdy. + rurality. +

dependency. + deprivation. + ethnic. + instability., data = infdf4m, subset =

ages. == "> 60 years", family = "binomial")

tab_model(model4a13.)

vif(model4a13.)

#ROC

ro10 <- subset(infdf4m, ages. == "> 60 years")

roc(ro10$exp3., model4a13.$fitted.values, plot = T,

legacy.axes = T, percent = T, xlab = "False Positive Percentage",

ylab = "True Positive Percentage", col = "blue", lwd = 3, print.auc = T)

#<= 3 encounters

model4a14 <- glm(exp3. ~ rc. + hr. + access_4c. + sex_rpdb. + fin_sit. + edu. + lang. +

rh_1. + ADG. + progtype. + fdy. + rurality. +

dependency. + deprivation. + ethnic. + instability., data = a4,

subset = num_encounters_1yr. == "<= 3 encounters", family = "binomial")

vif(model4a14)

tab_model(model4a14)

#remove inf. outliers

plot(model4a14, which = 5)

cooks4d014 <- cooks.distance(model4a14)

plot(cooks4d014, pch = "*", cex = 2, main = "Influential observations by Cooks distance")

abline(h = (4/1651), col = "red")

inf4n <- as.numeric(names(cooks4d014)[(cooks4d014 > (4/1651))])

head(inf4n)

infdf4n <- a4[-inf4n,]

model4a14. <- glm(exp3. ~ rc. + hr. + access_4c. + sex_rpdb. + fin_sit. + edu. + lang. +

rh_1. + ADG. + progtype. + fdy. + rurality. +

dependency. + deprivation. + ethnic. + instability., data = infdf4n, subset =

num_encounters_1yr. == "<= 3 encounters", family = "binomial")

tab_model(model4a14.)

vif(model4a14.)

#ROC

ro11 <- subset(infdf4n, num_encounters_1yr. == "<= 3 encounters")

roc(ro11$exp3., model4a14.$fitted.values, plot = T,

legacy.axes = T, percent = T, xlab = "False Positive Percentage",

ylab = "True Positive Percentage", col = "blue", lwd = 3, print.auc = T)

#> 3 encounters

model4a15 <- glm(exp3. ~ rc. + hr. + access_4c. + sex_rpdb. + fin_sit. + edu. + lang. +

rh_1. + ADG. + progtype. + fdy. + rurality. +

dependency. + deprivation. + ethnic. + instability., data = a4,

subset = num_encounters_1yr. == "> 3 encounters", family = "binomial")

vif(model4a15)

tab_model(model4a15)

#remove inf. outliers

plot(model4a15, which = 5)

cooks4d015 <- cooks.distance(model4a15)

plot(cooks4d015, pch = "*", cex = 2, main = "Influential observations by Cooks distance")

abline(h = (4/1041), col = "red")

inf4o. <- as.numeric(names(cooks4d015)[(cooks4d015 > (4/1041))])

head(inf4o.)

infdf4o <- a4[-inf4o.,]

model4a15. <- glm(exp3. ~ rc. + hr. + access_4c. + sex_rpdb. + fin_sit. + edu. + lang. +

rh_1. + ADG. + progtype. + fdy. + rurality. +

dependency. + deprivation. + ethnic. + instability., data = infdf4o, subset =

num_encounters_1yr. == "> 3 encounters", family = "binomial")

tab_model(model4a15.)

vif(model4a15.)

#ROC

ro11 <- subset(infdf4o, num_encounters_1yr. == "> 3 encounters")

roc(ro11$exp3., model4a15.$fitted.values, plot = T,

legacy.axes = T, percent = T, xlab = "False Positive Percentage",

ylab = "True Positive Percentage", col = "blue", lwd = 3, print.auc = T)

#exp4 ----

#significant association in age and comfortable financial sit (0.018)

#signficant association between number of encounters and adg

model4a016 <- glm(exp4. ~ rc. + hr. + access_4c. + sex_rpdb. + fin_sit.*age_rpdb + edu. + lang. +

rh_1. + ADG.*num_encounters_1yr + progtype. + fdy. + rurality. +

dependency. + deprivation. + ethnic. + instability., data = a4, family = "binomial")

summary(model4a016)

#full model

model4a16 <- glm(exp4. ~ rc. + hr. + access_4c. + sex_rpdb. + fin_sit. + edu. + lang. +

rh_1. + ADG. + progtype. + fdy. + rurality. +

dependency. + deprivation. + ethnic. + instability., data = a4, family = "binomial")

anova(model4a16, test = "Chisq")

tab_model(model4a16)

#remove inf. outliers

plot(model4a16, which = 5)

cooks4d016 <- cooks.distance(model4a16)

plot(cooks4d016, pch = "*", cex = 2, main = "Influential observations by Cooks distance")

abline(h = (4/2692), col = "red")

inf4p. <- as.numeric(names(cooks4d016)[(cooks4d016 > (4/2692))])

head(inf4p.)

infdf5p <- a4[-inf4p.,]

model4a16. <- glm(exp4. ~ rc. + hr. + access_4c. + sex_rpdb. + fin_sit. + edu. + lang. +

rh_1. + ADG. + progtype. + fdy. + rurality. +

dependency. + deprivation. + ethnic. + instability., data = infdf5p, family = "binomial")

tab_model(model4a16.)

vif(model4a16.)

#ROC

roc(infdf5p$exp4., model4a16.$fitted.values, plot = T,

legacy.axes = T, percent = T, xlab = "False Positive Percentage",

ylab = "True Positive Percentage", col = "blue", lwd = 3, print.auc = T)

#< 60 years

model4a17 <- glm(exp4. ~ rc. + hr. + access_4c. + sex_rpdb. + fin_sit. + edu. + lang. +

rh_1. + ADG. + progtype. + fdy. + rurality. +

dependency. + deprivation. + ethnic. + instability., data = a4,

subset = ages. == "<= 60 years", family = "binomial")

vif(model4a17)

tab_model(model4a17)

#remove inf. outliers

plot(model4a17, which = 5)

cooks4d017 <- cooks.distance(model4a17)

plot(cooks4d017, pch = "*", cex = 2, main = "Influential observations by Cooks distance")

abline(h = (4/1381), col = "red")

inf4q. <- as.numeric(names(cooks4d017)[(cooks4d017 > (4/1381))])

head(inf4q.)

infdf4q. <- a4[-inf4q.,]

model4a17. <- glm(exp4. ~ rc. + hr. + access_4c. + sex_rpdb. + fin_sit. + edu. + lang. +

rh_1. + ADG. + progtype. + fdy. + rurality. +

dependency. + deprivation. + ethnic. + instability., data = infdf4q., subset =

ages. == "<= 60 years", family = "binomial")

tab_model(model4a17.)

vif(model4a17.)

#ROC

ro12 <- subset(infdf4q., ages. == "<= 60 years")

roc(ro12$exp4., model4a17.$fitted.values, plot = T,

legacy.axes = T, percent = T, xlab = "False Positive Percentage",

ylab = "True Positive Percentage", col = "blue", lwd = 3, print.auc = T)

#> 60 years

model4a18 <- glm(exp4. ~ rc. + hr. + access_4c. + sex_rpdb. + fin_sit. + edu. + lang. +

rh_1. + ADG. + progtype. + fdy. + rurality. +

dependency. + deprivation. + ethnic. + instability., data = a4,

subset = ages. == "> 60 years", family = "binomial")

vif(model4a18)

tab_model(model4a18)

#remove inf. outliers

plot(model4a18, which = 5)

cooks4d018 <- cooks.distance(model4a18)

plot(cooks4d018, pch = "*", cex = 2, main = "Influential observations by Cooks distance")

abline(h = (4/1311), col = "red")

inf4r. <- as.numeric(names(cooks4d018)[(cooks4d018 > (4/1311))])

head(inf4r.)

infdf4r <- a4[-inf4r.,]

model4a18. <- glm(exp4. ~ rc. + hr. + access_4c. + sex_rpdb. + fin_sit. + edu. + lang. +

rh_1. + ADG. + progtype. + fdy. + rurality. +

dependency. + deprivation. + ethnic. + instability., data = infdf4r, subset =

ages. == "> 60 years", family = "binomial")

tab_model(model4a18.)

vif(model4a18.)

#ROC

ro13 <- subset(infdf4r, ages. == "> 60 years")

roc(ro13$exp4., model4a18.$fitted.values, plot = T,

legacy.axes = T, percent = T, xlab = "False Positive Percentage",

ylab = "True Positive Percentage", col = "blue", lwd = 3, print.auc = T)

#<= 3 encounters

model4a19 <- glm(exp4. ~ rc. + hr. + access_4c. + sex_rpdb. + fin_sit. + edu. + lang. +

rh_1. + ADG. + progtype. + fdy. + rurality. +

dependency. + deprivation. + ethnic. + instability., data = a4,

subset = num_encounters_1yr. == "<= 3 encounters", family = "binomial")

vif(model4a19)

tab_model(model4a19)

#remove inf. outliers

plot(model4a19, which = 5)

cooks4d019 <- cooks.distance(model4a19)

plot(cooks4d019, pch = "*", cex = 2, main = "Influential observations by Cooks distance")

abline(h = (4/1651), col = "red")

inf4r2 <- as.numeric(names(cooks4d019)[(cooks4d019 > (4/1651))])

head(inf4n)

infdf4r2 <- a4[-inf4r2,]

model4a19. <- glm(exp4. ~ rc. + hr. + access_4c. + sex_rpdb. + fin_sit. + edu. + lang. +

rh_1. + ADG. + progtype. + fdy. + rurality. +

dependency. + deprivation. + ethnic. + instability., data = infdf4r2, subset =

num_encounters_1yr. == "<= 3 encounters", family = "binomial")

tab_model(model4a19.)

vif(model4a19.)

#ROC

ro14 <- subset(infdf4r2, num_encounters_1yr. == "<= 3 encounters")

roc(ro14$exp4., model4a19.$fitted.values, plot = T,

legacy.axes = T, percent = T, xlab = "False Positive Percentage",

ylab = "True Positive Percentage", col = "blue", lwd = 3, print.auc = T)

#> 3 encounters

model4a20 <- glm(exp4. ~ rc. + hr. + access_4c. + sex_rpdb. + fin_sit. + edu. + lang. +

rh_1. + ADG. + progtype. + fdy. + rurality. +

dependency. + deprivation. + ethnic. + instability., data = a4,

subset = num_encounters_1yr. == "> 3 encounters", family = "binomial")

vif(model4a20)

tab_model(model4a20)

#remove inf. outliers

plot(model4a20, which = 5)

cooks4d020 <- cooks.distance(model4a20)

plot(cooks4d020, pch = "*", cex = 2, main = "Influential observations by Cooks distance")

abline(h = (4/1041), col = "red")

inf4s. <- as.numeric(names(cooks4d020)[(cooks4d020 > (4/1041))])

head(inf4s.)

infdf4s <- a4[-inf4s.,]

model4a20. <- glm(exp4. ~ rc. + hr. + access_4c. + sex_rpdb. + fin_sit. + edu. + lang. +

rh_1. + ADG. + progtype. + fdy. + rurality. +

dependency. + deprivation. + ethnic. + instability., data = infdf4s, subset =

num_encounters_1yr. == "> 3 encounters", family = "binomial")

tab_model(model4a20.)

vif(model4a20.)

#ROC

ro15 <- subset(infdf4s, num_encounters_1yr. == "> 3 encounters")

roc(ro15$exp4., model4a20.$fitted.values, plot = T,

legacy.axes = T, percent = T, xlab = "False Positive Percentage",

ylab = "True Positive Percentage", col = "blue", lwd = 3, print.auc = T)

#exp5 ----

#significant association in age and instability 4 (0.0147)

#signficant association between number of encounters and rc (0.035)

model4a021 <- glm(exp5. ~ rc.*num_encounters_1yr + hr. + access_4c. + sex_rpdb. + fin_sit. + edu. + lang. +

rh_1. + ADG. + progtype. + fdy. + rurality. +

dependency. + deprivation. + ethnic. + instability.*age_rpdb, data = a4, family = "binomial")

summary(model4a021)

#full model

model4a021 <- glm(exp5. ~ rc. + hr. + access_4c. + sex_rpdb. + fin_sit. + edu. + lang. +

rh_1. + ADG. + progtype. + fdy. + rurality. +

dependency. + deprivation. + ethnic. + instability., data = a4, family = "binomial")

anova(model4a021, test = "Chisq")

tab_model(model4a021)

#remove inf. outliers

plot(model4a021, which = 5)

cooks4d021 <- cooks.distance(model4a021)

plot(cooks4d021, pch = "*", cex = 2, main = "Influential observations by Cooks distance")

abline(h = (4/2692), col = "red")

inf4t. <- as.numeric(names(cooks4d021)[(cooks4d021 > (4/2692))])

head(inf4t.)

infdf5t <- a4[-inf4t.,]

model4a21. <- glm(exp5. ~ rc. + hr. + access_4c. + sex_rpdb. + fin_sit. + edu. + lang. +

rh_1. + ADG. + progtype. + fdy. + rurality. +

dependency. + deprivation. + ethnic. + instability., data = infdf5t, family = "binomial")

tab_model(model4a21.)

vif(model4a21.)

#ROC

roc(infdf5t$exp5., model4a21.$fitted.values, plot = T,

legacy.axes = T, percent = T, xlab = "False Positive Percentage",

ylab = "True Positive Percentage", col = "blue", lwd = 3, print.auc = T)

#< 60 years

model4a22 <- glm(exp5. ~ rc. + hr. + access_4c. + sex_rpdb. + fin_sit. + edu. + lang. +

rh_1. + ADG. + progtype. + fdy. + rurality. +

dependency. + deprivation. + ethnic. + instability., data = a4,

subset = ages. == "<= 60 years", family = "binomial")

vif(model4a22)

tab_model(model4a22)

#remove inf. outliers

plot(model4a22, which = 5)

cooks4d022 <- cooks.distance(model4a22)

plot(cooks4d022, pch = "*", cex = 2, main = "Influential observations by Cooks distance")

abline(h = (4/1381), col = "red")

inf4w. <- as.numeric(names(cooks4d022)[(cooks4d022 > (4/1381))])

head(inf4w.)

infdf4w. <- a4[-inf4w.,]

model4a22. <- glm(exp5. ~ rc. + hr. + access_4c. + sex_rpdb. + fin_sit. + edu. + lang. +

rh_1. + ADG. + progtype. + fdy. + rurality. +

dependency. + deprivation. + ethnic. + instability., data = infdf4w., subset =

ages. == "<= 60 years", family = "binomial")

tab_model(model4a22.)

vif(model4a22.)

#ROC

ro16 <- subset(infdf4w., ages. == "<= 60 years")

roc(ro16$exp5., model4a22.$fitted.values, plot = T,

legacy.axes = T, percent = T, xlab = "False Positive Percentage",

ylab = "True Positive Percentage", col = "blue", lwd = 3, print.auc = T)

#> 60 years

model4a23 <- glm(exp5. ~ rc. + hr. + access_4c. + sex_rpdb. + fin_sit. + edu. + lang. +

rh_1. + ADG. + progtype. + fdy. + rurality. +

dependency. + deprivation. + ethnic. + instability., data = a4,

subset = ages. == "> 60 years", family = "binomial")

vif(model4a23)

tab_model(model4a23)

#remove inf. outliers

plot(model4a23, which = 5)

cooks4d023 <- cooks.distance(model4a23)

plot(cooks4d023, pch = "*", cex = 2, main = "Influential observations by Cooks distance")

abline(h = (4/1311), col = "red")

inf4x. <- as.numeric(names(cooks4d023)[(cooks4d023 > (4/1311))])

head(inf4x.)

infdf4x <- a4[-inf4x.,]

model4a23. <- glm(exp5. ~ rc. + hr. + access_4c. + sex_rpdb. + fin_sit. + edu. + lang. +

rh_1. + ADG. + progtype. + fdy. + rurality. +

dependency. + deprivation. + ethnic. + instability., data = infdf4x, subset =

ages. == "> 60 years", family = "binomial")

tab_model(model4a23.)

vif(model4a23.)

#ROC

ro17 <- subset(infdf4x, ages. == "> 60 years")

roc(ro17$exp5., model4a23.$fitted.values, plot = T,

legacy.axes = T, percent = T, xlab = "False Positive Percentage",

ylab = "True Positive Percentage", col = "blue", lwd = 3, print.auc = T)

#<= 3 encounters

model4a24 <- glm(exp5. ~ rc. + hr. + access_4c. + sex_rpdb. + fin_sit. + edu. + lang. +

rh_1. + ADG. + progtype. + fdy. + rurality. +

dependency. + deprivation. + ethnic. + instability., data = a4,

subset = num_encounters_1yr. == "<= 3 encounters", family = "binomial")

vif(model4a24)

tab_model(model4a24)

#remove inf. outliers

plot(model4a24, which = 5)

cooks4d024 <- cooks.distance(model4a24)

plot(cooks4d024, pch = "*", cex = 2, main = "Influential observations by Cooks distance")

abline(h = (4/1651), col = "red")

inf4y <- as.numeric(names(cooks4d024)[(cooks4d024 > (4/1651))])

head(inf4y)

infdf4y <- a4[-inf4y,]

model4a24. <- glm(exp5. ~ rc. + hr. + access_4c. + sex_rpdb. + fin_sit. + edu. + lang. +

rh_1. + ADG. + progtype. + fdy. + rurality. +

dependency. + deprivation. + ethnic. + instability., data = infdf4y, subset =

num_encounters_1yr. == "<= 3 encounters", family = "binomial")

tab_model(model4a24.)

vif(model4a24.)

#ROC

ro18 <- subset(infdf4y, num_encounters_1yr. == "<= 3 encounters")

roc(ro18$exp5., model4a24.$fitted.values, plot = T,

legacy.axes = T, percent = T, xlab = "False Positive Percentage",

ylab = "True Positive Percentage", col = "blue", lwd = 3, print.auc = T)

#> 3 encounters

model4a25 <- glm(exp5. ~ rc. + hr. + access_4c. + sex_rpdb. + fin_sit. + edu. + lang. +

rh_1. + ADG. + progtype. + fdy. + rurality. +

dependency. + deprivation. + ethnic. + instability., data = a4,

subset = num_encounters_1yr. == "> 3 encounters", family = "binomial")

vif(model4a25)

tab_model(model4a25)

#remove inf. outliers

plot(model4a25, which = 5)

cooks4d025 <- cooks.distance(model4a25)

plot(cooks4d025, pch = "*", cex = 2, main = "Influential observations by Cooks distance")

abline(h = (4/1041), col = "red")

inf4z. <- as.numeric(names(cooks4d025)[(cooks4d025 > (4/1041))])

head(inf4z.)

infdf4z <- a4[-inf4z.,]

model4a25. <- glm(exp5. ~ rc. + hr. + access_4c. + sex_rpdb. + fin_sit. + edu. + lang. +

rh_1. + ADG. + progtype. + fdy. + rurality. +

dependency. + deprivation. + ethnic. + instability., data = infdf4z, subset =

num_encounters_1yr. == "> 3 encounters", family = "binomial")

tab_model(model4a25.)

vif(model4a25.)

#ROC

ro19 <- subset(infdf4z, num_encounters_1yr. == "> 3 encounters")

roc(ro19$exp5., model4a25.$fitted.values, plot = T,

legacy.axes = T, percent = T, xlab = "False Positive Percentage",

ylab = "True Positive Percentage", col = "blue", lwd = 3, print.auc = T)
