# Supplementary material for: The relationship between patient experience and real-world digital health access in primary care: A population-based cross-sectional study
Source: PLoS One. 2024 May 7;19(5):e0299005. doi: 10.1371/journal.pone.0299005 (PMC11075820; doi:10.1371/journal.pone.0299005)
Supplement: S6 Appendix — (DOCX) [file pone.0299005.s006.docx]

| Project InitiationThis Section must be Completed Prior to Project Dataset(s) Creation | | | | | |
| --- | --- | --- | --- | --- | --- |
| **Project Title:** | Understanding Associations between Patient Adoption of Digital Health Tools and Perceived Satisfaction of Care | | | | |
| **Project TRIM number:** | 2022 0908 020 020 | | | | |
| **Research Program:** | Life | | | | |
| **Site:** | ICES McMaster | | | | |
| **Project Objectives:** | *Insert Project Objectives as listed in the approved ICES Project PIA* | | | | |
|  | - Exploring the associations of digital health interventions on patient satisfaction towards primary care for Ontario adults. - Examine whether digital health interventions are associated with lower wait times for office visits due to health concerns. - Examine whether digital health interventions are associated with lower wait times for routine office visits. | | | | |
| **ICES Project PIA Initial Approval Date:** | *The ICES Employee or agent who is responsible for creating the Project Dataset(s) is responsible for ensuring there is an approved ICES Project PIA and verifying the date of approval prior to creating the Project Dataset(s)* | | | | |
|  | 2021-08-18 | | | | |
| **Principal Investigator (PI):** | Zain Pasat | | | | |
| **Check the applicable box if the PI is an ICES Student/Trainee** | ICES Student  ICES Fellow  ICES Post-Doctoral Trainee  Visiting Scholar | | | | |
| **Responsible ICES Scientist:** | *Name the Responsible ICES Scientist if the PI is not a Full Status ICES Scientist* | | | | |
|  | Dr. Andrew Costa | | | | |
| **Project Team Member(s) Responsible for Project Dataset Creation and/or Statistical Analysis and date joined (list all):** | *All person(s) (ICES Analyst, Appointed Analyst, Analytic Epidemiologist, PI, and/or Student) responsible for creating the Project Dataset(s) and/or statistical analysis on the Research Analytics Environment (RAE) and the date they joined the project must be recorded* | | | | |
|  | Richard Perez, Anastasia Gayowsky | | | 2021-11-24 | |
| **Other ICES Project Team Members and date joined (list all):** | *All other Research Project Team Members (e.g., Research Administrative Assistants, Research Assistants, Project Managers, Epidemiologists) and the date they joined the project must be recorded* | | | | |
|  | Dr. Cynthia Lokker, Dr. Jean-Eric Tarride | | | 2021-06-04 | |
| **Confirmation that DCP is consistent with Project Objectives:** | *The following individuals must confirm that the ICES Data provided for in this DCP is relevant (e.g., with respect to cohort, timeframe, and variables) and required to achieve the Project Objectives stated in the ICES Project PIA prior to initial Project Dataset creation: 1) PI; 2) Responsible ICES Scientist if the PI is not a Full Status ICES Scientist, or a second ICES Scientist or the Scientific Program Lead if the PI is creating both the DCP and the Project Dataset[s]; 3) ICES Research and Analysis Staff creating the DCP; and 4) ICES Analytic Staff (ICES Employee or agent responsible for creating the Project Dataset[s]). This may be delegated either verbally or via e-mail.* | | | | |
|  | ***Principal Investigator*** | |  | | 2021-11-30 |
|  | ***Responsible ICES Scientist or Second ICES Scientist/Lead*** | |  | 2021-10-25 | |
|  | ***ICES Research and Analysis Staff Creating the DCP*** | |  | yyyy-mon-dd | |
|  | ***ICES Analytic Staff*** | |  | yyyy-mon-dd | |
| **Designated ICES Research and Analysis Staff accountable for Project Documentation:** | *The person named (ICES staff) is accountable for ensuring that the approved ICES Project PIA, ICES Project PIA Amendments, and DCP are saved on the T Drive, ensuring ICES Project PIA Amendments are submitted as required, ensuring DCP Amendments are documented, and sharing the final DCP with the PI/Responsible ICES Scientist at project completion* | | | | |
|  | Erin O’Leary | | | | |
| **DCP Creation Date and Author:** | *Date DCP was finalized prior to Project Dataset(s) creation* | *Name of person who created the DCP* | | | |
|  | ***Date*** | ***Name*** | | | |
|  | 2021-11-30 | Zain Pasat | | | |

| ICES DataThis Section must be Completed Prior to Project Dataset(s) Creation | |
| --- | --- |
| *The ICES Employee or agent who is responsible for creating the Project Dataset(s) must ensure that this list includes only data listed in the ICES Project PIA*  *Changes to this list after initial ICES Project PIA approval require an ICES Project PIA Amendment* | *Mandatory for all datasets that are available by individual year* |
| ***General Use Datasets – Health Services*** | ***Years (where applicable)*** |
| OHIP | 2010 – 2020 |
| ***General Use Datasets – Care Providers*** |  |
| CPDB | 2010 – 2020 |
| IPDB | 2010 – 2020 |
| ***General Use Datasets – Population*** |  |
| CENSUS | 2010 – 2020 |
| RPDB | 2010 – 2020 |
| CONTACT | 2010 – 2020 |
| ***General Use Datasets - Other*** |  |
| ONMARG | 2010 – 2020 |
| CAPE | 2010 – 2020 |
| ***Other Datasets*** |  |
| HCES | 2012 – 2020 |
| ASTHMA | 2010 – 2020 |
| CHF | 2010 – 2020 |
| COPD | 2010 – 2020 |
| DEMENTIA | 2010 – 2020 |
| HIV | 2010 – 2020 |
| HYPER | 2010 – 2020 |
| OCCC | 2010 – 2020 |
| ODD | 2010 – 2020 |
| OMID | 2010 – 2020 |
| ORAD | 2010 – 2020 |

| Project Amendments and Reconciliation | | | |
| --- | --- | --- | --- |
| **ICES Project PIA Amendment History (add additional rows as needed):** | *Privacy approval date* | *Person who submitted amendment* | *Note that any changes to the list of ICES Data or Project Objectives require an ICES Project PIA Amendment* |
|  | ***Date*** | ***Name*** | ***Amendment*** |
|  | 2021-11-25 | Zain Pasat | Addition of study objectives and ICES data holding |
| **DCP Amendment History (add additional rows as needed):** | *Date DCP amended* | *Person who made the DCP amendment* | *Note that any DCP amendments involving changes to the list of ICES Data or Project Objectives require an ICES Project PIA Amendment* |
|  | ***Date*** | ***Name*** | ***Amendment*** |
|  | yyyy-mon-dd |  |  |
| **Date Programs/DCP reconciled** | *The person(s) creating the dataset and/or analyzing the data are responsible for ensuring that the final DCP reflects the final program(s) when the project is completed* | | |
|  | yyyy-mon-dd | | |

| Project Cohort | | |
| --- | --- | --- |
| **Study Design** | Cohort study  Matched cohort study  Case-control study  Cross-sectional study  Other (specify): | |
| **Index Event / Inclusion Criteria** | Participants who completed the HCES from 2012 until 2020. | |
| **Estimated Size of Cohort**  **(if known)** | 7527 respondents | |
| **Exclusions (in order)** | *Step* | Description |
|  | 1 | No exclusions |
|  | 2 |  |
|  | 3 |  |

| Project Time Frame Definitions | | |
| --- | --- | --- |
| Look-back Window  Observation Window  (in which to look for outcomes)  **Index Event Date**  Accrual Window  Max Follow-up Date | |  |
| **Accrual Start/End Dates** | 2012 - 2020 |  |
| **Max Follow-up Date** | 2020 |  |
| **When does observation window terminate?** | 2020 |  |
| **Lookback Window(s)** | 2010 |  |

| Variable Definitions (add additional rows as needed) | | |
| --- | --- | --- |
| **HCES** | **Please include ALL HCES variables from ALL waves in addition to those already specified below** |  |
| **Main Exposure or Risk Factor**  HCES | Patient used online appointment booking through website/portal  Variable: access_4c |  |
| HCES | Patient communicated with provider through video call  Variable: access_11a |  |
| HCES | Patient communicated with provider through messaging  Variable: access_12a |  |
| HCES | Patient communicated with provider through other digital means  Variable: access_13 |  |
| HCES | Patient viewed medical records online  Variable: dh_1A |  |
| HCES | Patient viewed medical records specific to health condition digitally  Variable: dh_2 |  |
| HCES | Patient accessed comprehensive health records digitally  Variable: dh_4 |  |
| HCES | Patient used virtual visit with provider other than their own  Variable: dh_5a |  |
| **Primary Outcome Definition**  HCES | Overall patient experience (summed scale)  Variables: Provider knows medical history [EXP_1] + Provider gives opportunity to ask questions [EXP_2] + Provider spends enough time with the patient [EXP_3] + Patient involved in decision making [EXP_4] + Provider explanations are easy to understand [EXP_5]  Note: Recode variables prior to addition as follows: Always 🡪 5, Often 🡪 4, Sometimes 🡪 3, Rarely 🡪 2, Never 🡪 1 |  |
| HCES | Provider knows medical history  Variable: EXP_1 |  |
| HCES | Provider gives opportunity to ask questions  Variable: EXP_2 |  |
| HCES | Provider spends enough time with the patient  Variable: EXP_3 |  |
| HCES | Patient involved in decision making  Variable: EXP_4 |  |
| HCES | Provider explanations are easy to understand  Variable: EXP_5 |  |
| **Secondary Outcome Definition(s)**  HCES | Time to an appointment in office for routine check-ups  Variable: sick_7b |  |
| HCES | Time to an appointment in office due to illness or health concern  Variable: sick_3 |  |
| **Baseline Characteristics**  HCES | Patient gender  Variable: SEX |  |
| HCES | Local Health Integration Network  Variable: LHIN |  |
| HCES | Patient chronic conditions (high blood pressure, hypertension)  Variable: RH_2A |  |
| HCES | Patient chronic conditions (diabetes)  Variable: RH_2B |  |
| HCES | Patient chronic conditions (arthritis)  Variable: RH_2C |  |
| HCES | Patient chronic conditions (heart disease, heart attack)  Variable: RH_2D |  |
| HCES | Patient chronic conditions (cancer)  Variable: RH_2E |  |
| HCES | Patient chronic conditions (asthma)  Variables: RH_2F, RH_2G, RH_2J |  |
| HCES | Patient chronic conditions (depression, mental health problems)  Variable: RH_2H |  |
| HCES | Patient chronic conditions (other long-term disease)  Variable: RH_2I |  |
| HCES | Patient chronic conditions (high cholesterol)  Variable: RH_2K |  |
| HCES | Number of chronic diseases  Variable: CHRONIC |  |
| ASTHMA | Patient chronic conditions (asthma)  Variable: ADMDATE  Notes: If admission date is available, indicate patient has asthma |  |
| CHF | Patient chronic conditions (congestive heart failure)  Variable: DIAGDATE  Notes: If diagnosis date is available, indicate patient has congestive heart failure |  |
| COPD | Patient chronic conditions (chronic obstructive pulmonary disease)  Variable: DIAGDATE  Notes: If diagnosis date is available, indicate patient has chronic obstructive pulmonary disease |  |
| DEMENTIA | Patient chronic conditions (dementia)  Variable: DIAGSRC  Notes: If diagnosis available, indicate patient has dementia |  |
| HIV | Patient chronic conditions (HIV)  Variable: DIAGDATE  Notes: If diagnosis date is available, indicate patient has HIV |  |
| HYPER | Patient chronic conditions (hypertension)  Variable: DIAGDATE  Notes: If diagnosis date is available, indicate patient has hypertension |  |
| OCCC | Patient chronic condition (Crohn’s disease)  Variable: DX_INCIDENT  Notes: if response is Crohn’s, indicate patient has Crohn’s disease |  |
| OCCC | Patient chronic condition (UC)  Variable: DX_INCIDENT  Notes: if response is UC, indicate patient has UC |  |
| ODD | Patient chronic conditions (diabetes)  Variable: DIAGDATE  Notes: If diagnosis date is available, indicate patient has diabetes |  |
| OMID | Patient had heart attack  Variable: ADMCAT |  |
| ORAD | Patient chronic conditions (rheumatoid arthritis)  Variable: DIAGDATE  Notes: If diagnosis date is available, indicate patient has rheumatoid arthritis |  |
| HCES | Patient overall perceived health  Variable: RH_1 |  |
| HCES | Patient level of education  Variable: EDU |  |
| HCES | Patient income  Variable: INC_CAT |  |
| CAPE | Identify which physician the patient is rostered to from survey completion  Variable: PROGTYPE, PHYSNUM, GRPNUM |  |
| CPDB | Identify which physicians use family health teams  Variable: FHT, FHTNUM_ENC, GRPNUM, GRPTYPE, CURRENT_MODEL_DESCRIPTION  Note: get OHIP visits and see which physicians they used the most within 1-2 years |  |
| IPDB | Identify which physicians use family health teams  Variable: PRACTYPE, PHYSNUM, OHSPEC |  |
| RPDB | Patient Rurality Index of Ontario (RIO)  Variable: PSTLCODE  Notes: Match postal code with RIO score |  |
| OHIP | If patient is not rostered under physician under CAPE, use OHIP billings during 2-year lookback from survey date to identify physician with most frequent billings. After identifying physician, link to IPDB and CPDB and get requested variables mentioned above. |  |
| RPDB | Forward sortation area (FSA)  Variable: PSTLCODE  Notes: Use first 3 characters |  |
| RPDB | Patient age  Variable: BDATE  Notes: Use date of birth to find the patient age |  |
| RPBD | Patient sex  Variable: SEX |  |
| HCES | Patient Rurality Index of Ontario (RIO)  Variable: PSTLCODE  Notes: If prior RPDB postal code is unavailable, derive RIO from postal code provided by HCES |  |
| ONMARG | Deprivation Factor Score  Variable: DEPRIVATION_DA  Notes: Assign patients to deprivation quintiles based on DA individual resides |  |
| ONMARG | Dependency Factor Score  Variable: DEPENDENCY_DA  Notes: Assign patients to dependency quintiles based on DA individual resides |  |
| ONMARG | Deprivation Quintile  Variable: DEPRIVATION_Q_DA  Notes: Used for imputation of missing HCES income data |  |
| ONMARG | Ethnic Concentration Factor Score  Variable: ETHNICCON_DA  Notes: Assign patients to ethnicity quintiles based on DA individual resides |  |
| ONMARG | Residential Instability Factor Score  Variable: INSTABILITY_DA  Notes: Assign patients to instability quintiles based on DA individual resides |  |
| **Other Variables**  HCES | Patient age (10-year categories)  Variable: AGECAT |  |
| HCES | Patient age (5 categories)  Variable: AGE3CAT |  |
| HCES | Patient age  Variable: AGE |  |
| OHIP | Patient rate of primary care visits in the last 12 months  Note: within 12 month look-back, calculate the number of visits to a primary care physician |  |
| OHIP | Patient rate of primary care visits in the last 24 months  Note: within 12 month look-back, calculate the number of visits to a primary care physician |  |
| HCES | Patient satisfaction with health care in the community  Variable: W1 |  |

| Analysis Plan and Dummy Tables (expand/modify as needed) | | |
| --- | --- | --- |
| **Descriptive Tables (insert or append dummy tables), e.g.:** | | |
| **Table 1. Baseline characteristics according to primary/secondary exposure** | | |
| **Baseline characteristics variables:**   - Gender: proportion - Age: mean - Level of education: proportion - Chronic conditions (0, 1-2, 3 or more): proportion - Overall perceived health: proportion - Income: proportion - Deprivation Factor Score: proportion - Rurality (urban, mid-urban, rural, and remote): proportion - Patient enrollment type: proportion - Rate of primary care visits over past 12 months: mean - Patient satisfaction with health care in the community: proportion | | |
| **Table 2. Adoption of Digital Health Interventions** | | |
| **Ontario adults using digital health tools:**   - Online appointment booking through website/portal: proportion - Video communication with provider: proportion - Text-based communication with provider: proportion - Other digital means of communication with provider: proportion - Used virtual care with provider other than their own: proportion - Online access to medical records: proportion - Online access to medical records specific to health condition: proportion - Online access to comprehensive medical records: proportion | | |
| **Table 2. Outcomes according to primary/secondary exposure** | | |
| **Association between patient experience and digital health tools**   - Online appointment booking through website/portal - Video communication with provider - Text-based communication with provider - Other digital means of communication with provider - Used virtual care with provider other than their own - Online access to medical records - Online access to medical records specific to health condition - Online access to comprehensive medical records   Note: potentially stratify by age, rate of primary care visits in past 12 months, and overall patient satisfaction with health care in the community | | |
| **Table 3. Covariates (baseline characteristics) according to outcomes** | | |
| **Statistical Model(s)** | | |
| **Type of model** | Linear regression | |
| **Primary independent variable** | - Online appointment booking through website/portal - Video communication with provider - Text-based communication with provider - Other digital means of communication with provider - Used virtual care with provider other than their own - Online access to medical records - Online access to medical records specific to health condition - Online access to comprehensive medical records | |
| **Dependent variable** | Patient experience | |
| **Covariates** | - Gender - Chronic conditions - Overall perceived health - Level of education - Income - Enrollment type - Rurality Index of Ontario - Deprivation Factor Score | |
| **Sensitivity Analyses** |  | |
| **Type of model** | Multilevel regression | |
| **Primary independent variable** | - Online appointment booking through website/portal - Video communication with provider - Text-based communication with provider - Other digital means of communication with provider - Used virtual care with provider other than their own - Online access to medical records - Online access to medical records specific to health condition - Online access to comprehensive medical records | |
| **Dependent variable** | Patient experience | |
| **Covariates** | - Gender - Chronic conditions - Overall perceived health - Level of education - Income - Enrollment type - Rurality Index of Ontario - Deprivation Factor Score | |
|  | |  |

| Quality Assurance Activities | | | |
| --- | --- | --- | --- |
| **RAE Directory of SAS Programs** |  | | |
| **RAE Directory of Final Dataset(s)** | *The* *final analytic dataset for each cohort includes all the data required to create the baseline tables and run all the models. It should include all covariates for all models such as patient risk factors, hospital characteristics, physician characteristics, exposure measures (continuous, categorical) and outcomes. It should include covariates that were considered but didn’t make the final cut. This would permit an analyst to easily re-run the models in the future.* | | |
|  |  | | |
| **RAE README file available:** Yes No | | | |
| **Date results of quality assurance tools for final dataset shared with project team (where applicable):** | | |  |
|  | | **%assign** | yyyy-mon-dd |
|  | | **%evolution** | yyyy-mon-dd |
|  | | **%dinexplore** | yyyy-mon-dd |
|  | | **%track / %exclude** | yyyy-mon-dd |
|  | | **%codebook** | yyyy-mon-dd |
| **Additional comments:** | |  | |
